# Ecological Momentary Assessment of Self-Reported Kratom Use, Effects, and Motivations Among US Adults

**DOI:** 10.1001/jamanetworkopen.2023.53401

**Published:** 2024-01-26

**Authors:** Kirsten E. Smith, Leigh V. Panlilio, Jeffrey D. Feldman, Oliver Grundmann, Kelly E. Dunn, Christopher R. McCurdy, Albert Garcia-Romeu, David H. Epstein

**Affiliations:** 1 Real-World Assessment, Prediction, and Treatment Unit, National Institute on Drug Abuse Intramural Research Program, Baltimore, Maryland; 2 Department of Psychiatry and Behavioral Sciences, Johns Hopkins University School of Medicine, Baltimore, Maryland; 3 Department of Medicinal Chemistry, College of Pharmacy, University of Florida, Gainesville

## Abstract

**Importance:**

Kratom products, which are sold legally in most of the US, contain alkaloids with opioidergic, adrenergic, and serotonergic activity. Millions of people use kratom to relieve pain, improve mood, or self-manage substance use disorders (SUDs). Kratom use has primarily been examined via surveys, in which recall biases among satisfied users may lead to minimization of transient negative outcomes. Further prospective study of kratom use, such as with ecological momentary assessment (EMA), is needed.

**Objective:**

To characterize proximal motivators, effects, and patterns of kratom use and to assess whether use frequency is associated with motivations, effects, past-year criteria for SUD for kratom (KUD), or other substance use.

**Design, Setting, and Participants:**

For this prospective cross-sectional study, an intensive longitudinal smartphone-based EMA in which participants’ current behaviors and experiences were repeatedly sampled in real time was conducted between July 1 and October 31, 2022. Participants comprised a convenience sample of US adults who used kratom at least 3 days per week for at least 4 weeks at the time of online screening. Criteria for past-year KUD were based on the *Diagnostic and Statistical Manual of Mental Disorders, Fifth Edition*. Data analysis was performed between November 2022 and November 2023.

**Exposure:**

The exposure was 13 401 kratom-use events across 15 days.

**Main Outcomes and Measures:**

A baseline survey covering demographics, health, kratom attitudes and behaviors, use motivations, other substance use, and KUD was administered before EMA. Data for the following EMA entries were then collected: event-contingent entries for kratom use (product, dose, and proximal motivations), follow-up entries (short-term effects and consequences of use events), random-prompt entries (mood), beginning-of-day entries (effects of kratom on sleep), and end-of-day entries (daily subjective descriptions of kratom effects). Bayesian regression was used to estimate means and credible intervals.

**Results:**

A total of 357 participants completed the EMA. Their mean (SD) age was 38.0 (11.1) years; more than half were men (198 [55.5%]). Participants reported overall motivators of use on the baseline survey that involved managing psychiatric and SUD problems, but proximal motivators evaluated during the EMA involved situation-specific needs such as increasing energy and productivity and decreasing pain. Acute effects were considered congruent with daily obligations. Use patterns, despite having some distinguishing features, were generally similar in their motivators and effects; participants used kratom predominantly during the daytime and seemed to find use frequencies that suited their needs. Higher use patterns were associated with symptoms of physical dependence (eg, withdrawal or tolerance). Co-used substances included caffeine, nicotine, vitamins, and cannabis.

**Conclusions and Relevance:**

Most participants in this study reported using kratom in a seemingly nonproblematic way. When such use appeared problematic, the key element was usually that withdrawal avoidance became a proximal motivator. Longitudinal studies examining changes in kratom use patterns and effects over time are needed.

## Key Points

**Question:**

When people use kratom regularly, how and why do they do so and with what effects?

**Findings:**

Among the 357 kratom consumers surveyed using ecological momentary assessment in this cross-sectional study, most reported using kratom daily to relieve pain, improve mood, or increase productivity, and some used it as an opioid substitute. Most participants reported improvements in daily living and productivity; more frequent use was associated with tolerance, withdrawal, and craving but not with social or functional impairment.

**Meaning:**

These findings suggest that some adults use kratom instrumentally (ie, to achieve specific goals) rather than recreationally, but not without indicators of physical dependence for some that require clinical study.

## Teaser

This cross-sectional study uses ecological momentary assessment to examine self-reported patterns, proximal motivators, and effects of kratom use among US adults.

## Introduction

Preparations derived from the leaves of kratom (*Mitragyna speciosa* Korth.), a tree in the coffee family, are classified by the US Food and Drug Administration as an unapproved new dietary ingredient but sold as natural wellness products.^1^ Kratom alkaloids act at the adenosine, sympathetic, opioid, and serotonin receptors.^2,3,4,5,6,7^

Millions of US adults use kratom^6,8,9,10^ to self-manage mood, fatigue, pain, and substance use disorders (SUDs) and for recreation.^10,11,12^ Kratom use has been examined through retrospective surveys, in which respondents who see their use as moderate and beneficial may minimize instances that disconfirm those beliefs (eg, they used more than intended or with transient negative outcomes).

In this study, we examined kratom use prospectively with smartphone-based ecological momentary assessment (EMA), in which participants described their moods and behaviors throughout the day. We investigated dosing routines, co-use of other substances, and the degree to which kratom use reliably led to desired effects and were compatible with responsibilities. To explore discrepancies and distinguish broad vs momentary motivations, we compared the EMA to a baseline survey of the same participants. A final aim was to differentiate subgroups or clusters of consumers based on kratom use patterns assessed via EMA and to determine whether more frequent users showed greater signs of problematic use.

## Methods

We have published our methods separately,^13^ so we provide only a summary here. This cross-sectional study using EMA was approved by the National Institutes of Health Institutional Review Board. Participants provided informed consent. The study followed the Strengthening the Reporting of Observational Studies in Epidemiology (STROBE) reporting guideline.

### Study Sample, Recruitment, and Screening

We recruited participants nationwide between July 1 and October 31, 2022. Inclusion criteria were as follows: kratom use (≥3 times weekly for ≥4 weeks), age 18 years or older, US residency, smartphone ownership, willingness to submit a kratom sample, a passing score on an online informed consent quiz, and English-language proficiency. Exclusion criteria were as follows: screening via a virtual private network or incarceration. Eligible candidates were emailed a link to our informed consent form, consent quiz, and survey, which we treated as a pre-EMA baseline measure. The survey covered demographics (self-reported age, sex, race and ethnicity, sexual orientation, education, employment status, and income), health, kratom attitudes and behaviors, use motivations, and past-year criteria for SUD for kratom (KUD). These criteria were based on the *Diagnostic and Statistical Manual of Mental Disorders, Fifth Edition* (*DSM-5*). We included race and ethnicity to better characterize the study sample. Participants self-identified as Asian, Black or African American (hereinafter Black), Hispanic or Latino (hereinafter Hispanic), Indian, Middle Eastern, Native American or Pacific Islander, White or European (hereinafter White), or multiple races or ethnicities.

Participants were emailed an invitation to download the study app (created with MetricWire) and, within 2 business days, to begin the 15-day EMA phase. Participants set time windows for random EMA prompts for each day of the week.

### EMA Sampling Strategy and Questionnaires

Participants were instructed to log each kratom-use event in an event-contingent entry, wherein they documented the product, dose, and motivations. The first 2 event-contingent entries of each day generated prompts for follow-up entries (randomly delivered 15-180 minutes later) assessing short-term kratom use effects and additional use. To avoid disincentivizing reporting, additional event-contingent entries did not generate follow-ups. Participants made 2 random-prompt entries during waking hours, reporting mood and any additional kratom use.

Participants also made beginning-of-day entries (assessing sleep) and end-of-day entries (assessing overall kratom effects that day and reporting [within 5 time bins] any unreported kratom use). These entries were scheduled to accord with sleep and wake times. To remain compliant, participants could miss no more than 1 prompt per day. The survey and EMA questions are presented in eAppendix 1 in Supplement 1.

### Statistical Analysis

Because the study design was not experimental, sample-size determination was premised not on null-hypothesis testing but on precision of estimation,^14^ which maximizes power for tests of associations or differences. Data are presented for the full sample and within use-pattern clusters that we defined by their within-day patterns of use (eAppendix 2 in Supplement 1). To identify within-day patterns of use at a moderately granular time scale, all use times from EMA were sorted into 24 bins of 1 hour each (aligned by each participant’s self-scheduled beginning-of-day time) for each participant. Data were decomposed into 2 independent metrics: (1) relative to the participant’s own average and (2) relative to the average of all participants. These were then categorized using finite mixture models with the FlexMix package, version 2.3.19^15^ in R software, version 4.3.2 (R Project for Statistical Computing). The optimal solution (based on information criteria) revealed 5 distinct clusters (clusters A-E); use frequency was highest in cluster A and successively lower through cluster E. Each participant was in only 1 cluster.

We treated use-pattern cluster as a between-groups factor for analysis of EMA-derived outcomes, using bayesian regression models with 2 R packages: brms, version 2.20.1 and ordbetareg, version 0.7.2.^16,17^ Each model used the most appropriate distributional family (linear, robust linear, ordinal, categorical, or ordered β) for the outcome; this provided a framework for interpretation, asking whether use frequency was associated with motivations or consequences (eg, using more than intended). Regression results are shown as conditional-effects plots with 90% credible intervals; nonoverlapping intervals provide conservative evidence that values differ. Data analysis was performed between November 2022 and November 2023.

## Results

Of the 2815 people screened, 1972 were eligible and 1152 were invited to enroll in this study. A total of 395 people enrolled, and 357 (90.4%) completed all EMA days (Table). The mean (SD) age of EMA completers was 38.0 (11.1) years; 198 (55.5%) were men, 149 (41.7%) were women, and 10 (2.8%) were nonbinary. Completers identified as Asian (9 [2.5%]), Black (10 [2.8%]), Hispanic (22 [6.2%]), Indian (1 [0.3%]), Middle Eastern (5 [1.4%]), Native American or Pacific Islander (14 [3.9%]), White (325 [91.0%]), or multiple races or ethnicities (17 [4.8%]).

**Table.** Demographic Characteristics of Participants^a^

Characteristic	Participant group		
	EMA completers (n = 357)	Noncompleters (n = 38)	Total enrolled (N = 395)
Age, mean (SD), y	38.0 (11.1)	38.3 (11.9)	38.1 (11.2)
Sex or gender			
Male	198 (55.5)	19 (50.0)	217 (54.9)
Female	149 (41.7)	18 (47.4)	167 (42.3)
Nonbinary	10 (2.8)	1 (2.6)	11 (2.8)
Race and ethnicity			
Asian	9 (2.5)	3 (7.9)	12 (3.0)
Black or African American	10 (2.8)	1 (2.6)	11 (2.8)
Hispanic or Latino	22 (6.2)	3 (7.9)	25 (6.3)
Indian	1 (0.3)	0	1 (0.3)
Middle Eastern	5 (1.4)	0	5 (1.3)
Native American or Pacific Islander	14 (3.9)	3 (7.9)	17 (4.3)
White or European	325 (91.0)	34 (89.5)	359 (90.9)
Multiple	17 (4.8)	1 (2.6)	18 (4.5)
Sexual orientation			
Heterosexual	282 (79.0)	29 (76.3)	311 (78.7)
Gay or lesbian	11 (3.1)	2 (5.3)	13 (3.3)
Bisexual	35 (9.8)	6 (15.8)	41 (10.4)
Asexual	5 (1.4)	0	5 (1.3)
Queer	8 (2.2)	0	8 (2.0)
Do not know	2 (0.6)	0	2 (0.5)
Prefer not to say	8 (2.2)	0	8 (2.0)
Other	6 (1.7)	1 (2.6)	7 (1.8)
Education			
Some college	118 (33.1)	12 (31.6)	130 (32.9)
Associate or vocational degree	71 (19.9)	10 (26.3)	81 (20.5)
Bachelor’s degree	67 (18.8)	8 (21.1)	75 (19.0)
High school or GED	57 (16.0)	4 (10.5)	61 (15.4)
Master’s degree	28 (7.8)	3 (7.9)	31 (7.9)
PhD, MD, or JD	9 (2.6)	0	9 (2.6)
Did not finish high school	7 (2.0)	1 (2.6)	8 (2.0)
Primary employment status			
Full-time	201 (56.3)	22 (57.9)	223 (56.5)
Part-time	48 (13.5)	5 (13.2)	53 (13.4)
Disabled	43 (12.0)	1 (2.6)	44 (11.1)
Unemployed	41 (11.5)	5 (13.1)	46 (11.7)
Student	15 (4.2)	4 (10.5)	19 (4.8)
Retired	9 (2.5)	1 (2.6)	10 (2.5)
Annual income, $			
0-10 000	57 (16.0)	4 (10.5)	61 (15.4)
10 001-40 000	151 (42.3)	16 (42.1)	167 (42.3)
40 001-70 000	74 (20.7)	9 (23.7)	83 (21.0)
70 001-100 000	37 (10.4)	7 (18.4)	44 (11.1)
100 001-150 000	25 (7.0)	2 (5.3)	27 (6.8)
150 001-200 000	13 (3.6)	0	13 (3.3)
Longest period of uninterrupted kratom use since initiation^b^			
1-3 mo	12 (3.5)	2 (6.5)	14 (3.8)
3-6 mo	12 (3.5)	2 (6.5)	14 (3.8)
6-12 mo	50 (14.7)	9 (29.0)	59 (15.9)
1-2 y	64 (18.8)	5 (16.1)	69 (18.5)
2-5 y	137 (40.2)	7 (22.6)	144 (38.7)
>5 y	66 (19.4)	6 (19.4)	72 (19.4)
Currently in alcohol or drug recovery	135 (37.8)	10 (26.3)	145 (36.7)
How did you hear about the study?^c^			
Reddit	131 (36.7)	14 (36.8)	145 (36.7)
Facebook	77 (21.6)	11 (28.9)	88 (22.3)
American Kratom Association	62 (17.4)	4 (10.5)	66 (16.7)
Friend or family	39 (10.9)	4 (10.5)	43 (10.9)
Online retailer or vendor that sells kratom	20 (5.6)	2 (5.3)	22 (5.6)
Podcast	14 (3.9)	1 (2.6)	15 (3.8)
Online forum or private group	18 (5.0)	1 (2.6)	19 (4.8)
Twitter	9 (2.5)	1 (2.6)	10 (2.5)
Local kratom advocacy group	7 (2.0)	0	7 (1.8)
Instagram	6 (1.7)	2 (5.3)	8 (2.0)
Store that sells kratom	5 (1.4)	1 (2.6)	6 (1.6)
NIDA research team member	3 (0.8)	1 (2.6)	4 (1.0)
Customers or coworkers at a kratom shop	2 (0.6)	0	2 (0.5)

Abbreviations: EMA, ecological momentary assessment; GED, General Educational Development; NIDA, National Institute on Drug Abuse.

^a^ Unless stated otherwise, values are No. (%) of participants.

^b^ Excluding 23 responses, which are unquantifiable.

^c^ Choices are not mutually exclusive.

### Use-Pattern Clusters

There were 13 401 kratom-use events reported, with a mean (SEM) of 2.52 (0.06) uses per participant per day. Products comprising whole-leaf kratom were most common (eTable 1 in Supplement 1). The mean number of uses per hour was about 5 times higher in cluster A than cluster E, decreasing stepwise from clusters A through E. The modal within-day pattern (ie, more uses in the morning) was more pronounced in higher-use clusters A and B. Most participants used kratom daily, but the probability of daily use decreased from clusters A through E (eFigure 1 in Supplement 1). There were no discernible day-of-the-week effects (eg, more use on weekends). The typical use location was usually home and sometimes work; activities associated with use were primarily related to waking up, working, or daily living (eg, chores or hygiene; eFigure 2 in Supplement 1). All clusters tended to use more in the first half of their waking hours than at other times of day, as shown in eAppendix 2 in Supplement 1.

eTable 2 in Supplement 1 presents demographic differences across clusters. Men comprised more than half of participants in clusters A, C, D, and E (29 of 56 [51.8%], 42 of 71 [59.2%], 47 of 83 [56.6%], and 50 of 80 [62.5%]), respectively, and women comprised slightly more than half of participants in cluster B (33 of 65 [50.8%]). Participants in clusters A through E had a mean (SD) age of 42.4 (12.1), 37.3 (9.6), 39.7 (11.3), 36.6 (10.5), and 36.5 (11.2) years, respectively. Race and ethnicity distribution was similar across clusters. Cluster A was emblematic in that these participants were motivated to use kratom as a long-term opioid substitute, yet the number in SUD recovery was 25 (44.6%), 26 (40.0%), 34 (47.9%), 28 (33.3%), and 22 (27.2%) across clusters A through E and was highest in cluster C, meaning that many participants were in recovery but not all were necessarily motivated to use kratom as an opioid substitute.

Mean compliance (ie, the percentage of random prompts answered) was 90.4% among participants who completed the EMA phase (n = 357). As described previously in our methods article,^13^ compliance did not differ significantly by any characteristic.

**Figure 1.**
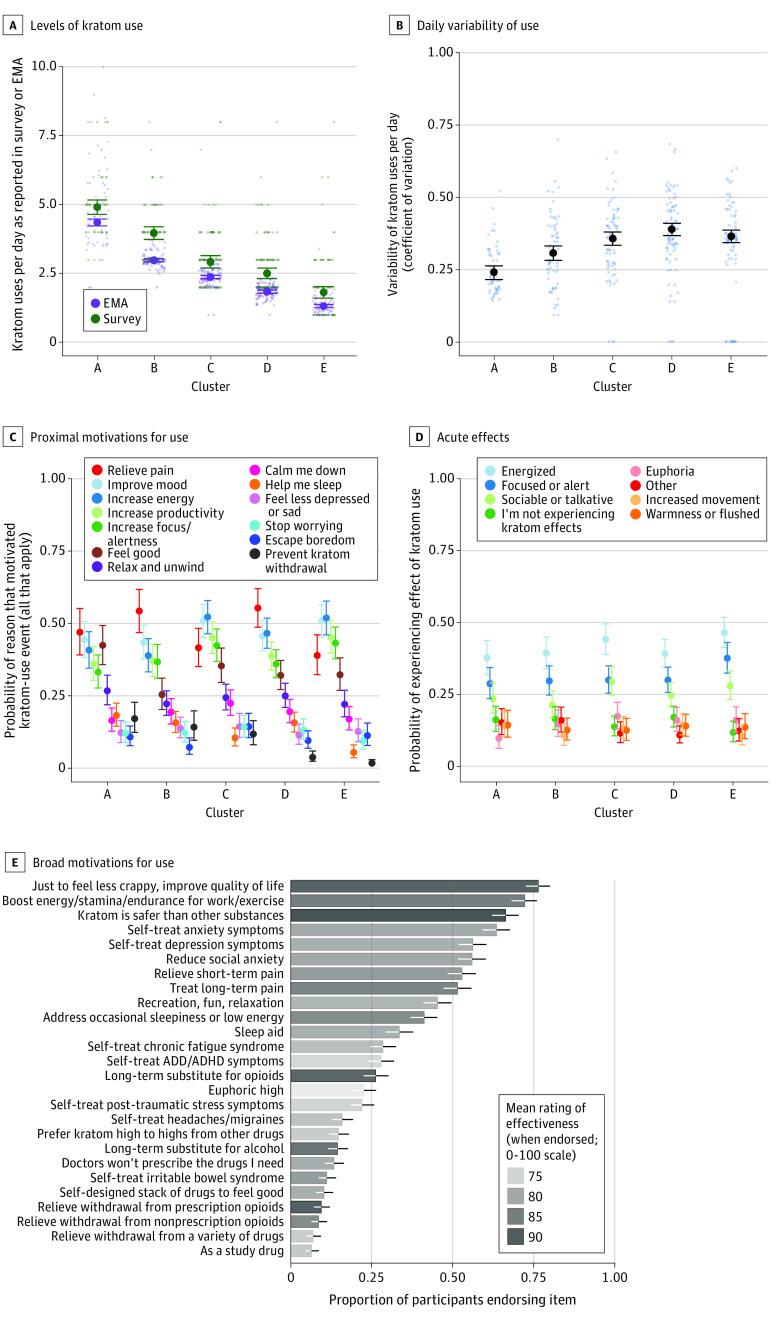
Characteristics of Kratom Use Obtained From Baseline Survey and Ecological Momentary Assessment (EMA) A, Mean number of uses per day as reported in the survey (referring to the 30 days before the survey, which was conducted before EMA) and in EMA reports that indicated time of each use event. Clusters represent subgroups of participants with similar patterns of use (most frequent in cluster A, and least frequent in cluster E). B, Daily variability in the number of uses per day in each cluster (expressed as the coefficient of variation), with the frequent-use clusters A and B showing less variation. C, Proximal motivations endorsed in event-contingent reports of use. Motivations were generally similar across clusters, but variability was lowest in frequent-use clusters A and B. D, Acute effects of kratom, as described in follow-up reports obtained at random times within 2 hours after a use-event report. Effects were generally similar across clusters. E, Broad motivations for kratom use across all participants (ie, regardless of cluster), with bar length indicating the proportion of participants endorsing the item and with shading indicating the average rating of kratom's effectiveness for each purpose, as reported in the survey of important factors that influence or motivate the participant’s kratom use. In A to E, large points and error bars represent the bayesian 90% credible interval for the measure. Small points (when present) represent data (randomly jittered horizontally) for individual participants. Nonoverlapping error bars provide a conservative indication of a reliable difference between conditions. In C to E, data were obtained by giving the participant a list from which all items that applied could be endorsed. Clustering was based in part on the EMA data summarized in the first panel, but also on within-day patterns of use (not shown). The legend (or order of bars in E) shows the items in order of decreasing overall prevalence (ie, in all participants, regardless of cluster); items that were not endorsed by at least 10% of at least 1 cluster were excluded from analysis. ADD indicates attention-deficit disorder; ADHD, attention-deficit/hyperactivity disorder.

Rates of kratom use in same-day EMA reports were slightly lower than in retrospective estimates according to the baseline survey (Figure 1A). Variability of daily use was greater in lower-use clusters D and E (Figure 1B).

### Motivations for Use

Broad motivations for use according to the baseline survey mostly involved general well-being or management of health conditions. In addition, participants rated kratom as being safer than other substances and highly effective at improving quality of life, boosting energy, replacing opioids or alcohol, and relieving opioid withdrawal (Figure 1E). Many participants used kratom as a long-term or short-term substitution for opioids (100 [28.0%] or 28 [7.8%], respectively) or alcohol (56 [15.7%] or 24 [6.7%], respectively); 19 (5.3%) reported using kratom as a long-term replacement for buprenorphine or methadone, and 39 (10.9%) reported using it to relieve withdrawal symptoms from these and other substances. Kratom use for long-term opioid substitution was more likely among the highest-use cluster A, and the lower-use clusters D and E were less likely to use kratom for prevention of kratom craving or withdrawal (eFigure 3 in Supplement 1).

Proximal motivations for a kratom-use event often involved situation-specific needs, such as increasing energy and focus or decreasing pain. Using kratom to feel good was common, but using it to feel high was not (Figure 1C). Middle-use cluster C had the greatest probability of using to feel high. The lower-use clusters were less likely to be motivated by withdrawal and craving, and the lowest-use cluster was less likely to use kratom for sleep (eFigure 4 in Supplement 1).

### Acute Effects

Acute effects according to follow-up entries largely aligned with proximal use motivations (Figure 1D). Participants typically reported feeling energized, focused or alert, sociable or talkative, and, to a lesser extent, euphoria, increased movement, and warmness or flushing. All clusters reported “not experiencing kratom effects” in approximately 10% of follow-up entries, on average, suggesting tolerance, delayed onset, or transience. Ratings of anxiety, pain, and sadness were approximately 0.25 (0-1 scale), and mood was usually positive (>0.50) (eFigure 5 in Supplement 1).

For 2 EMA items regarding craving (“Do you crave kratom right now?” and “Do you crave another drug right now?”), craving was greater for kratom than for other substances (eFigure 6 in Supplement 1). Clusters A and C tended to have higher kratom craving; participants in all clusters rarely reported having used more kratom than intended (eFigure 6 in Supplement 1).

### Kratom Co-Use Patterns

**Figure 2.**
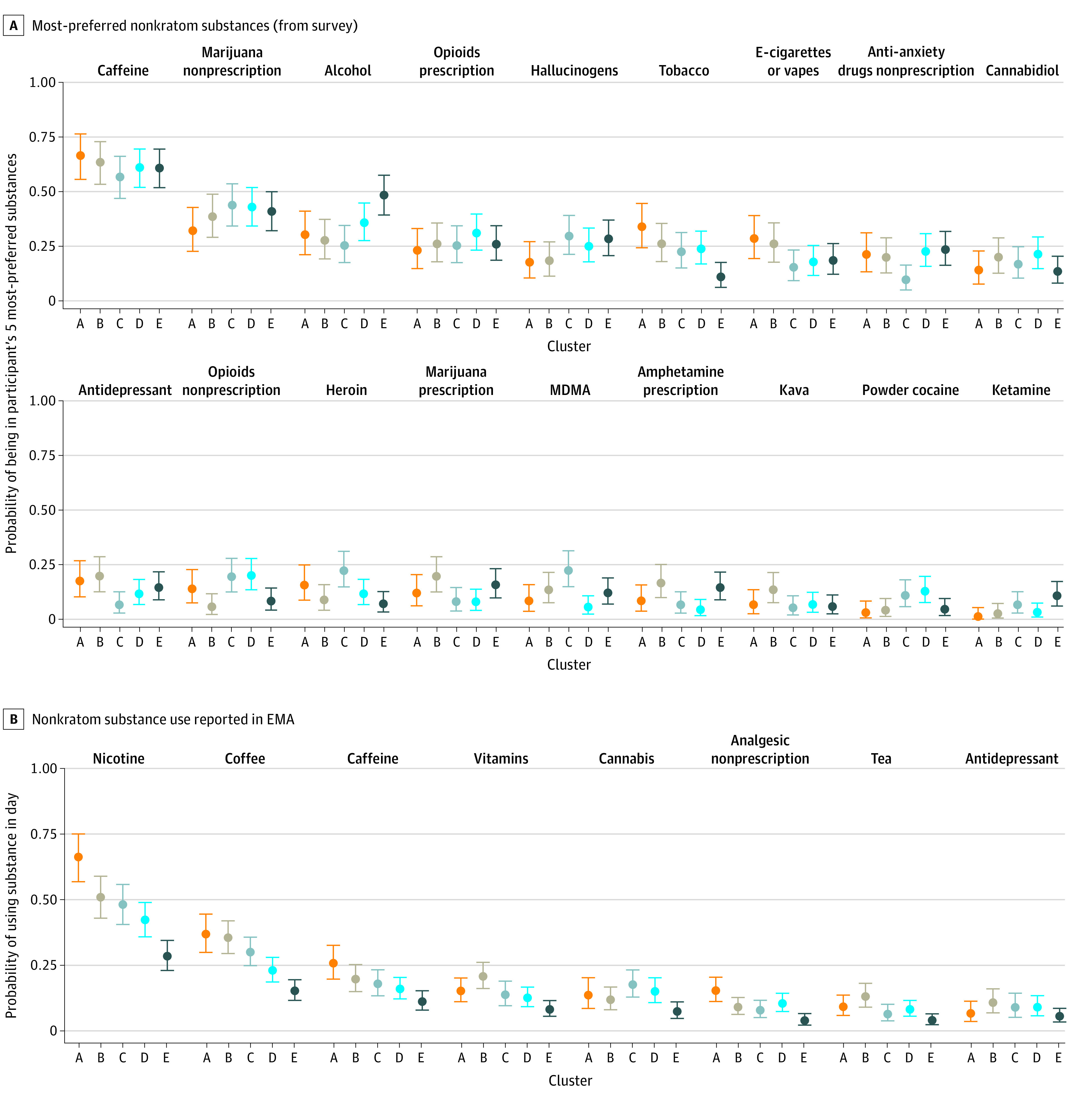
Nonkratom Substance Preferences and Use A, Most-preferred nonkratom substances, in response to the survey item (“Thinking about all of the drugs you have ever tried, please select your five most preferred substances, meaning the substances you received the most all around satisfaction from”). B, Nonkratom substances used during the study, as reported in the ecological momentary assessment (EMA). The mean and the 90% credible interval are reported for each cluster. Items are shown in order of decreasing overall prevalence (ie, across all participants); items that were not endorsed by at least 10% of at least 1 cluster were excluded from analysis. For many measures, the kratom-use clusters were similar to each other, with some clear exceptions (mostly in cluster E, who had the least frequent use) as discussed in the Results. MDMA indicates methylenedioxymethamphetamine.

The most-preferred nonkratom substances according to the baseline survey (Figure 2A) were caffeine and cannabis. These preferences were similar across clusters, although lowest-use cluster E was slightly more likely to prefer alcohol and less likely to prefer tobacco.

Based on the EMA item regarding other substances (“Which other substances have you taken during or since your last use of kratom?”), the most co-used drugs were nicotine and coffee or caffeine (Figure 2B), concordant with survey-stated preferences. Cluster A had the most nicotine users (and, among nicotine users, the highest probability of nicotine use on a given day); cluster E had the lowest. Other co-used substances were cannabis, antidepressant medications, and over-the-counter analgesics; co-use was generally lowest in cluster E. Clusters differed in probability of having ever received medications for opioid use disorder, with highest-use cluster A most likely and cluster E least likely (eFigure 7 in Supplement 1).

### Kratom and Daily Activities

**Figure 3.**
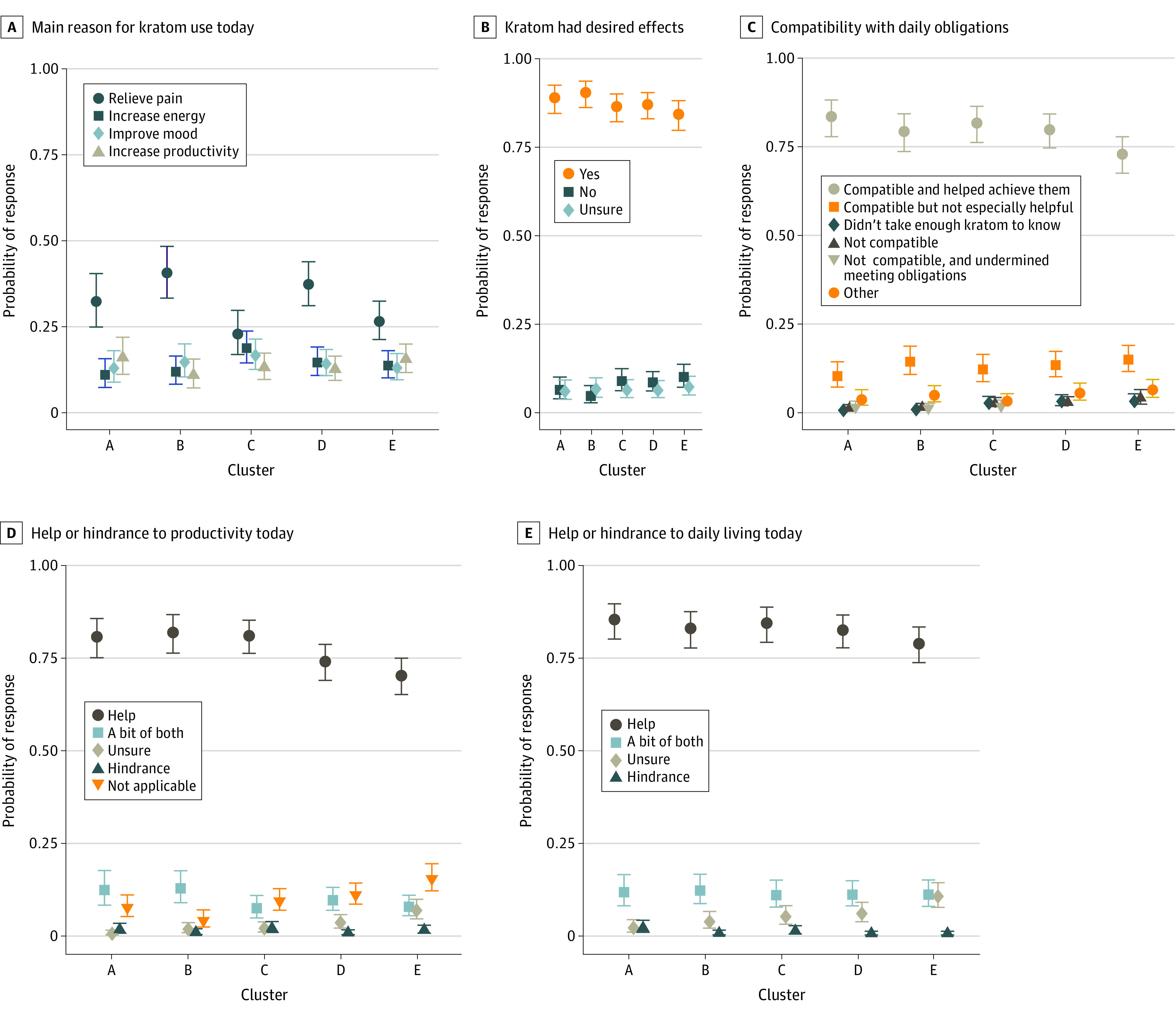
Motivations for and Effects of Kratom Use A, Main reason for kratom use that day. B, Judgement of whether kratom had the desired results and effects the participant wanted that day. C, Overall characterization of kratom effects experienced that day. D, Assessment of whether kratom was a help or hindrance to productivity that day. E, Assessment of whether kratom was a help or hindrance to participants being able to live out their day and do what they wanted or needed to do. Data in all panels are from end-of-day reports.

Primary motivations for using kratom on a given day according to end-of-day entries (Figure 3A) were consistent with proximal motivations reported at the time of use. All clusters used kratom for energy or productivity and mood improvement, and all rated their kratom doses as effective (Figure 3B). Kratom effects experienced each day were typically judged to be compatible and helpful with daily roles and obligations, and not a hindrance. Likewise, by a wide margin, kratom was judged to be helpful for daily productivity and daily living (Figure 3C, 3D, and 3E).

The first dose of the day occurred sooner in highest-use cluster A than in lower-use clusters D and E. Only Cluster E had a plurality taking their first dose more than 60 minutes after waking (eFigure 8 in Supplement 1).

Sleep quality and quantity according to beginning-of-day entries (eFigure 9 in Supplement 1) were mostly unaffected by kratom use the previous day or night in moderate- to low-use clusters C to E. Participants in cluster E were less likely than all other participants to report that kratom increased their sleep quality and quantity. The highest-use clusters A and B were about equally likely to report that kratom either improved sleep or did not affect sleep quality. There were few reports of increased or decreased sleep from not using kratom.

### Substance Use Disorder for Kratom and Physical Dependence

**Figure 4.**
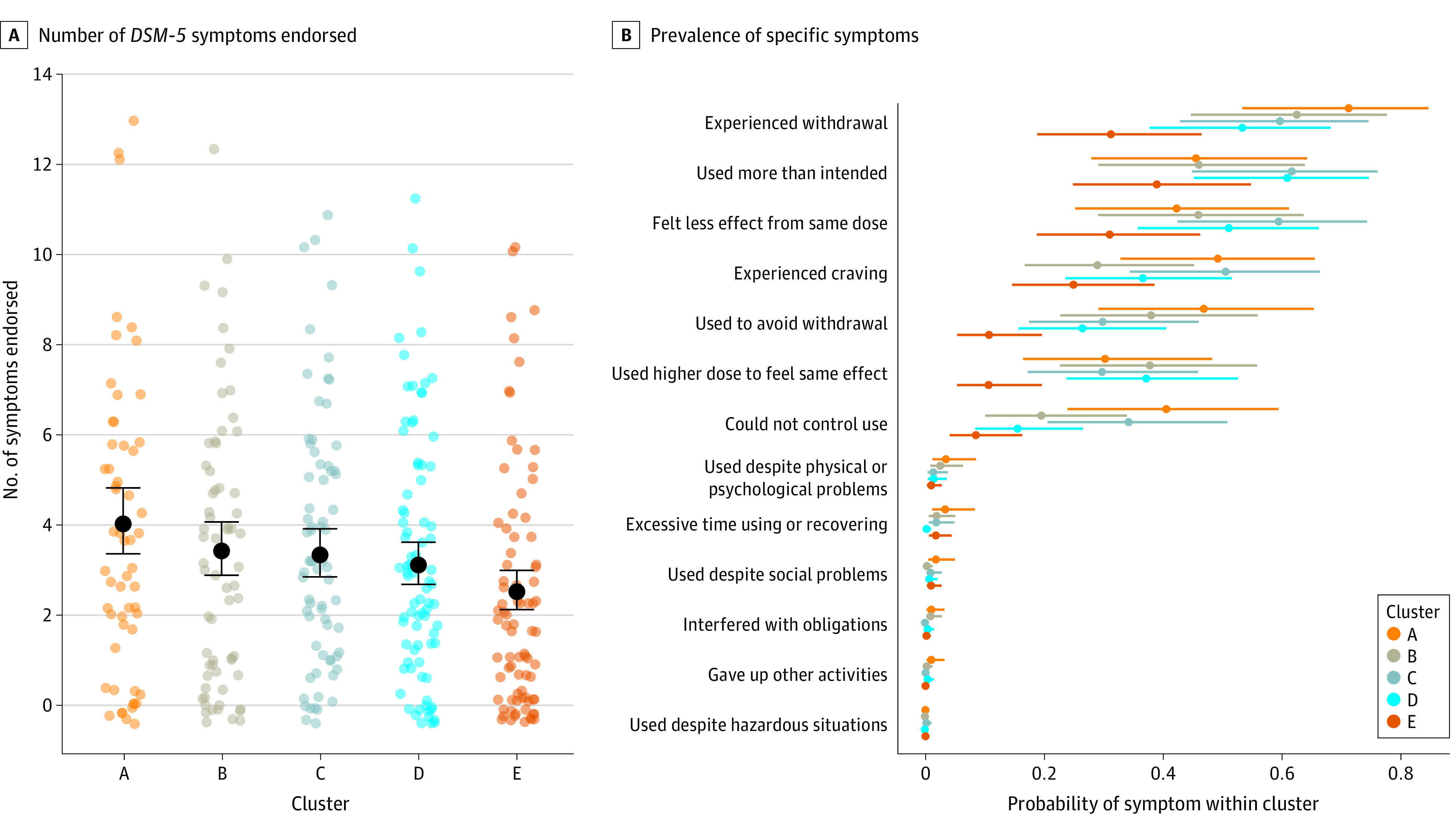
Symptoms Associated With Kratom Use A, Number of kratom-related *Diagnostic and Statistical Manual of Mental Disorders, Fifth Edition* (*DSM-5*) symptoms endorsed within the participant’s lifetime for each cluster (means in black) and for each participant (points, colored by cluster). B, Probability of endorsement of each *DSM*-*5* symptom within each cluster. Points and error bars represent means and 90% credible intervals.

In the survey, participants across clusters averaged 3 to 4 symptoms of *DSM-5*–based KUD, but each cluster also had a few participants with 10 or more symptoms (Figure 4A). Symptom counts were higher in cluster A compared with cluster E, and they were intermediate in the other clusters. The numbers of participants meeting criteria for KUD (≥2 symptoms) were 238 (66.7%) overall and 42 (75.0%), 41 (63.1%), 52 (73.2%), 59 (70.2%), and 44 (54.3%), respectively, across clusters A through E. Three participants (1 each in clusters A, B, and E) met criteria due only to tolerance and withdrawal. The most common symptoms (Figure 4B) were related to withdrawal, tolerance, using more than intended, and craving. In clusters A and C, respectively, 25 (44.6%) and 28 (39.4%) participants reported at least 1 unsuccessful attempt to reduce or quit kratom use. Symptoms reflecting psychosocial impairments or hazardous use were rare and reported by a few participants (in cluster A). In a baseline survey item regarding conceptualizations of kratom, approximately half of the participants endorsed “habit-forming” and nearly three-quarters endorsed “life-saving” (eFigure 10 in Supplement 1).

## Discussion

To our knowledge, this study is the first EMA of people who use kratom. The 15-day EMA period permitted a snapshot into daily patterns and consequences of use. Our findings were largely consistent with prior US survey data—never a foregone conclusion with EMA.^18^ Demographic characteristics were similar among clusters based on dosing frequency, although lowest-use cluster E tended to be younger and disproportionally male. Clusters were most differentiated by SUD recovery, with the lower-use clusters D and E having fewer members in recovery. Frequency of use appeared slightly greater in our baseline survey than in EMA; this could reflect some underreporting due to EMA response burden, along with rounding biases to which cross-sectional surveys are prone.^19^ In this regard, prospective momentary data add nuance to cross-sectional data.

### Reasons for Kratom Use at Different Temporal Resolutions

Many of the broad, non–mutually exclusive motivations reported at baseline were in accord with prior survey findings, including increasing energy, addressing psychiatric or physical problems, and reducing or avoiding opioid or alcohol use.^10,20,21,22^ However, in this EMA, the main proximal motivators of use (analgesia, mood improvement, and increased energy, focus, or productivity) did not typically include psychiatric or physical problems and very rarely included avoidance of other substance use. This discrepancy may reflect tonic dampening of broader perceived needs, such that each discrete use of kratom was seen as addressing a proximal goal. In interviews with a subset of participants,^23^ we found that kratom use was sometimes initiated for a perceived specific need (eg, to stop drinking, manage anxiety, relieve pain, or reduce opioid cravings), then continued for additional (and more general) daily-life purposes such as improving mood, productivity, or energy. Cluster A was emblematic in that these participants were motivated to use kratom as a long-term opioid substitute, yet the number in SUD recovery was 25 (44.6%), 26 (40.0%), 34 (47.9%), 28 (33.3%), and 22 (27.2%) across clusters A through E and was highest in cluster C, meaning that many participants were in recovery but not all were necessarily motivated to use kratom as a drug substitute. It is also unclear whether broad use motivations were associated with discrete use events, particularly as most people in the sample had been using kratom for years and most did not endorse decreasing other drug craving as the proximal motivator of kratom use. One possibility is that participants had been in recovery long enough that cravings were less frequent or simply attenuated by kratom.

### A Day in the Life With Kratom: Order and Some Elements of Disorder

Dosing was proximally motivated by wanting to feel good across clusters (especially in high-use cluster A), but rarely by wanting to feel high. Nonetheless, a frequent proximal motivation (especially in cluster A) was to avoid kratom withdrawal or stop kratom craving. Cluster A also disproportionately reported that they typically used kratom within 5 minutes of waking and conceptualized kratom as habit-forming and life-saving (Figure 1E and breakdown by cluster in eFigure 9 in Supplement 1). Yet middle-use cluster C had the greatest probability of using kratom to feel high. This finding suggests that despite the greater frequency of use that defined cluster A, the proximal motivator was not euphoria but possibly physical dependence that developed from long-term use of kratom for opioid substitution. Even in cluster A, which showed less variance in dosing routines than other clusters, kratom was seen as compatible with daily responsibilities, and social or functional impairment was rare. Participants in all clusters, often with years of regular use, usually reported that they still felt acute effects from each dose and that the kratom they took on a given day had the desired effects, which were perceived as compatible with or helpful for daily obligations.

That type of relationship with a substance can be described as successful instrumental use.^24^ Our prior survey and interview data support this conceptualization for at least some regular kratom consumers.^23,25^ With EMA follow-up entries, we probed for momentary acknowledgments that kratom was sometimes used in greater amounts than intended or that it briefly had net negative effects, but such occurrences were rare (eFigure 6 in Supplement 1). This is an important finding, underscoring that when instrumental use of kratom becomes problematic or disordered, the key element is that avoidance of withdrawal becomes a proximal motivator. Participants rarely reported social or occupational impairments or detriments to sleep that might obliquely impair functioning. This pattern of limited life disruption was also found in adults in Asia who used fresh kratom preparations.^26,27^

Kratom craving has been documented in Asia and was found in our EMA data. In this study, kratom craving was higher than for other substances across all clusters. The extent to which kratom craving can be unmanageable or bothersome requires further study. Participants who underwent in-depth interviews did not describe kratom craving or tolerance as intense and unmanageable, nor did they describe life impairments from kratom.^23^ However, participants acknowledged unknowns of long-term use, and 2 long-term kratom consumers professed to have experienced something like addiction or strong physical dependence; both planned to quit or reduce use.^23^ Longitudinal investigations are needed to determine whether instrumental use with minimal net negative effects endures. In this sample, 280 participants (78.4%) used kratom without interruption for a year or longer. The balance of benefit to burden could shift. For participants like those in cluster A, who were most likely to use kratom for long-term opioid substitution, the calculus is challenging to gauge: this group was least varied in use patterns and most likely to consider kratom life-saving but also experienced more KUD symptoms. Is this emblematic of a self-adopted maintenance therapy or harm-reduction practice, or of something more problematic? We leave that question unanswered but note that kratom use for opioid substitution has been documented since 2007 but has not been systematically studied.^6,10,20,21,25^

### Limitations

This study has some limitations. Our enrollment strategy was intended to minimize bias with respect to participant attitudes. We did not seek to exclude people with unfavorable attitudes about kratom. On an open-ended screening item, many candidates wrote that their motivation for enrolling was curiosity or to help clarify pros and cons of kratom; many, but not most, reported favorable attitudes toward kratom. Nonetheless, there may have been bias by not having data from people unwilling to participate. Among participants, some may not have been fully aware of adverse effects, such as diminutions in productivity or the emergence of negatively reinforced use. We view these findings as a picture of what kratom use can look like when it is not profoundly disruptive—not as evidence that it can never be profoundly disruptive. Indeed, of the 10 EMA participants interviewed, 1 conveyed an intent to quit^23^ and, at baseline, the higher-use clusters reported unsuccessful quit attempts (Figure 4B). Thus, a fundamental limitation is that our intensive longitudinal design did not assess within-person change on the timescale of a traditional longitudinal design. A test for within-person change would be a burst design, in which a cohort briefly undergoes EMA every few years.^28,29^ We hypothesize that in such a design, some participants’ instrumental use of kratom would remain stable, but some could deteriorate to moderate or severe KUD and others may quit kratom. Given that each participant chose products and doses that worked as desired, our results reflect how kratom is typically used, within a nonexperimental design. These findings should be interpreted in light of the fact that the sample was predominantly White and had at least some exposure to college. Finally, these findings generalize only to US adults using whole-plant kratom products regularly, not daily kratom extract users or infrequent consumers.

## Conclusions

This cross-sectional study using EMA was suited to detect signals of excessive or problematic kratom use that might be minimized by recall biases in surveys, but the findings largely strengthened what surveys had suggested: for some regular users, kratom can produce what are perceived as daily-life benefits such as increased energy, productivity, and mood enhancement. These findings were consistent across participant clusters defined by their differing frequencies of daily use. As noted, our sample does not represent everyone who uses kratom, particularly those who regularly use extracts rather than whole-leaf products. These data are one piece of what must be a multidisciplinary effort to inform US clinical practice, research, and policy.^30,31^ One complication is that some kratom products are not labeled with recommended serving amounts, warnings, or alkaloid content.^32,33,34,35^ In future studies, we will present further results in the context of laboratory assays of the products our participants used. Our present results can inform and constrain the design and interpretation of future studies. In the meantime, clinicians and researchers should be aware that among US adults who use whole-plant kratom products regularly, use may be best conceptualized as part of a routine geared toward daytime productivity more than recreation, but that physical dependence is possible. Future studies should purposefully sample daily kratom extract product consumers.

## Notes

Supplement 1. **eAppendix 1.** Cross-Sectional Survey and Ecological Momentary Assessment Questions **eAppendix 2.** Clustering Procedure
eReference
**eTable 1.** Kratom Dosage Forms and Units **eFigure 1.** Proportion of Days With Kratom Use **eFigure 2.** Locations and Activities When Using Kratom **eTable 2.** Demographic Characteristics by Kratom-Use Cluster (Clusters A-E) **eFigure 3. **Broad Motivations for Use **eFigure 4.** Proximal Motivations for Kratom Use **eFigure 5.** Anxiety, Pain, Mood, and Sadness **eFigure 6.** Craving and More Kratom Use Than Intended **eFigure 7.** Lifetime Medication for Opioid Use Disorder (Buprenorphine or Methadone) **eFigure 8.** Kratom Use After Waking **eFigure 9.** Kratom Effects on Sleep **eFigure 10.** Conceptualizations of Kratom

Supplement 2.
Data Sharing Statement
