# Supplementary material for: Ecological Momentary Assessment of Self-Reported Kratom Use, Effects, and Motivations Among US Adults
Source: JAMA Netw Open. 2024 Jan 26;7(1):e2353401. doi: 10.1001/jamanetworkopen.2023.53401 (PMC10818224; doi:10.1001/jamanetworkopen.2023.53401)
Supplement: Supplement 1. — eAppendix 1. Cross-Sectional Survey and Ecological Momentary Assessment Questions eAppendix 2. Clustering Procedure eReference eTable 1. Kratom Dosage Forms and Units eFigure 1. Proportion of Days With Kratom Use eFigure 2. Locations and Activities When Using Kratom eTable 2. Demographic Characteristics by Kratom-Use Cluster (Clusters A-E) eFigure 3. Broad Motivations for Use eFigure 4. Proximal Motivations for Kratom Use eFigure 5. Anxiety, Pain, Mood, and Sadness eFigure 6. Craving and More Kratom Use Than Intended eFigure 7. Lifetime Medication for Opioid Use Disorder (Buprenorphine or Methadone) eFigure 8. Kratom Use After Waking eFigure 9. Kratom Effects on Sleep eFigure 10. Conceptualizations of Kratom [file jamanetwopen-e2353401-s001.pdf]

Supplementary Online Content

Smith KE, Panlilio LV, Feldman JD, et al. Self-reported kratom use, effects, and motivations among US adults. *JAMA Netw Open*. 2024;7(1):e2353401. doi:10.1001/jamanetworkopen.2023.53401

eAppendix 1. Cross-Sectional Survey and Ecological Momentary Assessment Questions

eAppendix 2. Clustering Procedure

eReference

eTable 1. Kratom Dosage Forms and Units

eFigure 1. Proportion of Days With Kratom Use

eFigure 2. Locations and Activities When Using Kratom

eTable 2. Demographic Characteristics by Kratom-Use Cluster (Clusters A-E)

eFigure 3. Broad Motivations for Use

eFigure 4. Proximal Motivations for Kratom Use

eFigure 5. Anxiety, Pain, Mood, and Sadness

eFigure 6. Craving and More Kratom Use Than Intended

eFigure 7. Lifetime Medication for Opioid Use Disorder (Buprenorphine or Methadone)

eFigure 8. Kratom Use After Waking

eFigure 9. Kratom Effects on Sleep

eFigure 10. Conceptualizations of Kratom

This supplementary material has been provided by the authors to give readers additional information about their work.

© 2024 Smith KE et al. *JAMA Network Open.*

eAppendix 1. Cross-Sectional Survey and Ecological Momentary Assessment Questions

Cross-sectional survey and Ecological Momentary Assessment questions

(BEGINING OF CROSS-SECTIONAL SURVEY QUESTIONS)

Start of Block: Kratom and Other Drug Use History Questions

Q150 How did you learn about this study? ***Select all that apply.***

- ☐ From a friend or family (1)
- ☐ From an in-person shop that sell kratom (2)
- ☐ From a NIDA research team member (3)
- ☐ From an online retailer or vendor that sells kratom (4)
- ☐ A podcast (5)
- ☐ From a flyer (6)
- ☐ The American Kratom Association (7)
- ☐ A local kratom advocacy group (8)
- ☐ Reddit (9)
- ☐ Facebook (10)
- ☐ Twitter (11)
- ☐ Instagram (12)
- ☐ An online forum or private group (13)
- ☐ A university (14)
- ☐ I work in a kratom shop and I heard through customers or co-workers (15)
- ☐ I import, sell or distribute kratom and heard through my colleagues (16)
- ☐ Other (17) ____

© 2024 Smith KE et al. *JAMA Network Open.*

Q151 How old were you when you first used kratom?

____

Q156 Please tell us about how many days have you used kratom per week for each week ***during the past month***?

Q152 Days of kratom use per week this last week (past 7 days)
▼ 0 (0) ... 7 (7)

Q153 Days of kratom use per week ***1 week ago***
▼ 0 (0) ... 7 (7)

Q154 Days of kratom use per week ***2 weeks ago***
▼ 0 (0) ... 7 (7)

Q155 Days of kratom use per week ***3 weeks ago***
▼ 0 (0) ... 7 (7)

Page Break

© 2024 Smith KE et al. *JAMA Network Open.*

Q322 Please select from the list below any substance(s) you have ever used ***during your lifetime***. **Select all that apply.**

- ☐ Kratom (1)
- ☐ Alcohol (2)
- ☐ Caffeine (3)
- ☐ smoked tobacco: cigarettes, cigars, hookah (4)
- ☐ e-cigarettes/vapes (5)
- ☐ marijuana (pot, weed), not prescribed (6)
- ☐ medicinal marijuana, prescribed (7)
- ☐ CBD (cannabidiol) (8)
- ☐ synthetic marijuana (Serenity, Spice, K2) (9)
- ☐ prescription opioids not prescribed to you (10)
- ☐ opioids prescribed to you (11)
- ☐ methadone not prescribed to you (12)
- ☐ methadone prescribed to you (13)
- ☐ Suboxone/Subutex (buprenorphine) not prescribed to you (14)
- ☐ Suboxone/Subutex (buprenorphine) prescribed to you (15)
- ☐ tianeptine not prescribed to you (16)
- ☐ heroin (17)
- ☐ fentanyl (18)
- ☐ powder cocaine (19)
- ☐ crack/rock/freebase cocaine (20)

© 2024 Smith KE et al. *JAMA Network Open.*

- [ ] street meth (crystal meth, crank) (21)
- [ ] amphetamine pills (Ritalin, Adderall) not prescribed to you (22)
- [ ] amphetamine pills (Ritalin, Adderall) prescribed to you (23)
- [ ] Ecstasy/MDMA (24)
- [ ] Modafinil/Provigil not prescribed to you (25)
- [ ] DMT (26)
- [ ] bath salts (synthetic cathinones) (27)
- [ ] anti-anxiety drugs (Xanax, Valium, Ativan), not prescribed to you (28)
- [ ] anti-anxiety drugs (Xanax, Valium, Ativan), prescribed to you (29)
- [ ] hallucinogens (LSD, mushrooms) (30)
- [ ] phenibut (31)
- [ ] racetams (32)
- [ ] DXM/dextromethorphan (33)
- [ ] ketamine (34)
- [ ] kava (35)
- [ ] antidepressant medication prescribed to you (36)
- [ ] antidepressant medication not prescribed to you (37)
- [ ] antipsychotic medication prescribed to you (38)
- [ ] antipsychotic medication not prescribed to you (39)

Carry Forward Selected Choices from "Please select from the list below any substance(s) you have ever used during your lifetime. Select all that apply."

Q157 For the following substances that you have ever used during your lifetime, please fill in the age (in years) when you first tried it. If you can't remember exactly, just give your best guess.

© 2024 Smith KE et al. *JAMA Network Open.*

	**Age you first used** (1)
Kratom (x1)	
Alcohol (x39)	
Caffeine (x2)	
smoked tobacco: cigarettes, cigars, hookah (x3)	
e-cigarettes/vapes (x4)	
marijuana (pot, weed), not prescribed (x5)	
medicinal marijuana, prescribed (x6)	
CBD (cannabidiol) (x7)	
synthetic marijuana (Serenity, Spice, K2) (x8)	
prescription opioids not prescribed to you (x9)	

© 2024 Smith KE et al. *JAMA Network Open.*

opioids prescribed to you (x10)	
methadone not prescribed to you (x11)	
methadone prescribed to you (x12)	
Suboxone/Subutex (buprenorphine) not prescribed to you (x13)	
Suboxone/Subutex (buprenorphine) prescribed to you (x14)	
tianeptine not prescribed to you (x15)	
heroin (x16)	
fentanyl (x17)	
powder cocaine (x18)	
crack/rock/freebase cocaine (x19)	
street meth (crystal meth, crank) (x20)	

© 2024 Smith KE et al. *JAMA Network Open.*

amphetamine pills (Ritalin, Adderall) not prescribed to you (x21)	
amphetamine pills (Ritalin, Adderall) prescribed to you (x22)	
Ecstasy/MDMA (x23)	
Modafinil/Provigil not prescribed to you (x24)	
DMT (x25)	
bath salts (synthetic cathinones) (x26)	
anti-anxiety drugs (Xanax, Valium, Ativan), not prescribed to you (x27)	
anti-anxiety drugs (Xanax, Valium, Ativan), prescribed to you (x28)	
hallucinogens (LSD, mushrooms) (x29)	
phenibut (x30)	

© 2024 Smith KE et al. *JAMA Network Open.*

racetams (x31)	
DXM/dextromethorphan (x32)	
ketamine (x33)	
kava (x34)	
antidepressant medication prescribed to you (x35)	
antidepressant medication not prescribed to you (x36)	
antipsychotic medication prescribed to you (x37)	
antipsychotic medication not prescribed to you (x38)	

Page Break

Carry Forward Selected Choices from "Please select from the list below any substance(s) you have ever used during your lifetime. Select all that apply."

Q158 **Past 12-month Drug Use**
Please indicate the <u>total number of months</u> ***in the past 12 months*** that you used—*even if you only used one day during a given month (that still counts).*

Put (0) for any substances you did not use ***in the past 12 months***.

	**Total Number of Months Used**
Kratom (x1)	▼ 0 (1 ... 12 (12)
Alcohol (x39)	▼ 0 (1 ... 12 (12)
Caffeine (x2)	▼ 0 (1 ... 12 (12)
smoked tobacco: cigarettes, cigars, hookah (x3)	▼ 0 (1 ... 12 (12)
e-cigarettes/vapes (x4)	▼ 0 (1 ... 12 (12)
marijuana (pot, weed), not prescribed (x5)	▼ 0 (1 ... 12 (12)
medicinal marijuana, prescribed (x6)	▼ 0 (1 ... 12 (12)
CBD (cannabidiol) (x7)	▼ 0 (1 ... 12 (12)
synthetic marijuana (Serenity, Spice, K2) (x8)	▼ 0 (1 ... 12 (12)
prescription opioids not prescribed to you (x9)	▼ 0 (1 ... 12 (12)
opioids prescribed to you (x10)	▼ 0 (1 ... 12 (12)
methadone not prescribed to you (x11)	▼ 0 (1 ... 12 (12)
methadone prescribed to you (x12)	▼ 0 (1 ... 12 (12)
Suboxone/Subutex (buprenorphine) not prescribed to you (x13)	▼ 0 (1 ... 12 (12)
Suboxone/Subutex (buprenorphine) prescribed to you (x14)	▼ 0 (1 ... 12 (12)
tianeptine not prescribed to you (x15)	▼ 0 (1 ... 12 (12)
heroin (x16)	▼ 0 (1 ... 12 (12)
fentanyl (x17)	▼ 0 (1 ... 12 (12)
powder cocaine (x18)	▼ 0 (1 ... 12 (12)
crack/rock/freebase cocaine (x19)	▼ 0 (1 ... 12 (12)
street meth (crystal meth, crank) (x20)	▼ 0 (1 ... 12 (12)

© 2024 Smith KE et al. *JAMA Network Open.*

amphetamine pills (Ritalin, Adderall) not prescribed to you (x21)	▼ 0 (1 ... 12 (12)
amphetamine pills (Ritalin, Adderall) prescribed to you (x22)	▼ 0 (1 ... 12 (12)
Ecstasy/MDMA (x23)	▼ 0 (1 ... 12 (12)
Modafinil/Provigil not prescribed to you (x24)	▼ 0 (1 ... 12 (12)
DMT (x25)	▼ 0 (1 ... 12 (12)
bath salts (synthetic cathinones) (x26)	▼ 0 (1 ... 12 (12)
anti-anxiety drugs (Xanax, Valium, Ativan), not prescribed to you (x27)	▼ 0 (1 ... 12 (12)
anti-anxiety drugs (Xanax, Valium, Ativan), prescribed to you (x28)	▼ 0 (1 ... 12 (12)
hallucinogens (LSD, mushrooms) (x29)	▼ 0 (1 ... 12 (12)
phenibut (x30)	▼ 0 (1 ... 12 (12)
racetams (x31)	▼ 0 (1 ... 12 (12)
DXM/dextromethorphan (x32)	▼ 0 (1 ... 12 (12)
ketamine (x33)	▼ 0 (1 ... 12 (12)
kava (x34)	▼ 0 (1 ... 12 (12)
antidepressant medication prescribed to you (x35)	▼ 0 (1 ... 12 (12)
antidepressant medication not prescribed to you (x36)	▼ 0 (1 ... 12 (12)
antipsychotic medication prescribed to you (x37)	▼ 0 (1 ... 12 (12)
antipsychotic medication not prescribed to you (x38)	▼ 0 (1 ... 12 (12)

Page Break

© 2024 Smith KE et al. *JAMA Network Open.*

Carry Forward Unselected Choices from "Total Number of Months Used"

X→

Q159 **Past 30-day Drug Use**
Please indicate the ***total number of days*** you used ***in the past 30 days—****even if you only used a little bit on a given day (that still counts).*

Put (0) for any substances you did not use ***in the past 30 days***.

	Total Number of Days Used
Kratom (xx1)	▼ 0 (1 ... 30 (30)
Alcohol (xx39)	▼ 0 (1 ... 30 (30)
Caffeine (xx2)	▼ 0 (1 ... 30 (30)
smoked tobacco: cigarettes, cigars, hookah (xx3)	▼ 0 (1 ... 30 (30)
e-cigarettes/vapes (xx4)	▼ 0 (1 ... 30 (30)
marijuana (pot, weed), not prescribed (xx5)	▼ 0 (1 ... 30 (30)
medicinal marijuana, prescribed (xx6)	▼ 0 (1 ... 30 (30)
CBD (cannabidiol) (xx7)	▼ 0 (1 ... 30 (30)
synthetic marijuana (Serenity, Spice, K2) (xx8)	▼ 0 (1 ... 30 (30)
prescription opioids not prescribed to you (xx9)	▼ 0 (1 ... 30 (30)
opioids prescribed to you (xx10)	▼ 0 (1 ... 30 (30)
methadone not prescribed to you (xx11)	▼ 0 (1 ... 30 (30)
methadone prescribed to you (xx12)	▼ 0 (1 ... 30 (30)
Suboxone/Subutex (buprenorphine) not prescribed to you (xx13)	▼ 0 (1 ... 30 (30)
Suboxone/Subutex (buprenorphine) prescribed to you (xx14)	▼ 0 (1 ... 30 (30)
tianeptine not prescribed to you (xx15)	▼ 0 (1 ... 30 (30)
heroin (xx16)	▼ 0 (1 ... 30 (30)
fentanyl (xx17)	▼ 0 (1 ... 30 (30)
powder cocaine (xx18)	▼ 0 (1 ... 30 (30)
crack/rock/freebase cocaine (xx19)	▼ 0 (1 ... 30 (30)

© 2024 Smith KE et al. *JAMA Network Open.*

street meth (crystal meth, crank) (xx20)	▼ 0 (1 ... 30 (30)
amphetamine pills (Ritalin, Adderall) not prescribed to you (xx21)	▼ 0 (1 ... 30 (30)
amphetamine pills (Ritalin, Adderall) prescribed to you (xx22)	▼ 0 (1 ... 30 (30)
Ecstasy/MDMA (xx23)	▼ 0 (1 ... 30 (30)
Modafinil/Provigil not prescribed to you (xx24)	▼ 0 (1 ... 30 (30)
DMT (xx25)	▼ 0 (1 ... 30 (30)
bath salts (synthetic cathinones) (xx26)	▼ 0 (1 ... 30 (30)
anti-anxiety drugs (Xanax, Valium, Ativan), not prescribed to you (xx27)	▼ 0 (1 ... 30 (30)
anti-anxiety drugs (Xanax, Valium, Ativan), prescribed to you (xx28)	▼ 0 (1 ... 30 (30)
hallucinogens (LSD, mushrooms) (xx29)	▼ 0 (1 ... 30 (30)
phenibut (xx30)	▼ 0 (1 ... 30 (30)
racetams (xx31)	▼ 0 (1 ... 30 (30)
DXM/dextromethorphan (xx32)	▼ 0 (1 ... 30 (30)
ketamine (xx33)	▼ 0 (1 ... 30 (30)
kava (xx34)	▼ 0 (1 ... 30 (30)
antidepressant medication prescribed to you (xx35)	▼ 0 (1 ... 30 (30)
antidepressant medication not prescribed to you (xx36)	▼ 0 (1 ... 30 (30)
antipsychotic medication prescribed to you (xx37)	▼ 0 (1 ... 30 (30)
antipsychotic medication not prescribed to you (xx38)	▼ 0 (1 ... 30 (30)

Page Break

Carry Forward Selected Choices from "Please select from the list below any substance(s) you have ever used during your lifetime. Select all that apply."

Q160 **Kratom Co-Use**

Please select from the list below any substance you have ever used ***at the same time*** as kratom or ***within 1-2 hours*** of using kratom. **Select all that apply.**

- ☐ None (-2)
- ☐ I don't remember (-1)
- ☐ Grapefruit juice (0)
- ☐ Kratom (1)
- ☐ Alcohol (2)
- ☐ Caffeine (3)
- ☐ smoked tobacco: cigarettes, cigars, hookah (4)
- ☐ e-cigarettes/vapes (5)
- ☐ marijuana (pot, weed), not prescribed (6)
- ☐ medicinal marijuana, prescribed (7)
- ☐ CBD (cannabidiol) (8)
- ☐ synthetic marijuana (Serenity, Spice, K2) (9)
- ☐ prescription opioids not prescribed to you (10)
- ☐ opioids prescribed to you (11)
- ☐ methadone not prescribed to you (12)
- ☐ methadone prescribed to you (13)
- ☐ Suboxone/Subutex (buprenorphine) not prescribed to you (14)
- ☐ Suboxone/Subutex (buprenorphine) prescribed to you (15)
- ☐ tianeptine not prescribed to you (16)

© 2024 Smith KE et al. *JAMA Network Open.*

- heroin (17)
- fentanyl (18)
- powder cocaine (19)
- crack/rock/freebase cocaine (20)
- street meth (crystal meth, crank) (21)
- amphetamine pills (Ritalin, Adderall) not prescribed to you (22)
- amphetamine pills (Ritalin, Adderall) prescribed to you (23)
- Ecstasy/MDMA (24)
- Modafinil/Provigil not prescribed to you (25)
- DMT (26)
- bath salts (synthetic cathinones) (27)
- anti-anxiety drugs (Xanax, Valium, Ativan), not prescribed to you (28)
- anti-anxiety drugs (Xanax, Valium, Ativan), prescribed to you (29)
- hallucinogens (LSD, mushrooms) (30)
- phenibut (31)
- racetams (32)
- DXM/dextromethorphan (33)
- ketamine (34)
- kava (35)
- antidepressant medication prescribed to you (36)
- antidepressant medication not prescribed to you (37)
- antipsychotic medication prescribed to you (38)
- antipsychotic medication not prescribed to you (39)

© 2024 Smith KE et al. *JAMA Network Open.*

Page Break

© 2024 Smith KE et al. *JAMA Network Open.*

Carry Forward Selected Choices from "Please select from the list below any substance(s) you have ever used during your lifetime. Select all that apply."

Q161 Thinking about all of the drugs you have ever tried, **please select your five most preferred substances**, meaning the substances you received the most all around satisfaction from, by ranking them in the order of most satisfaction.

Here, a rank of 1 would be your most preferred substance whereas a rank of 5 would be your fifth most preferred substance.

______ Kratom (1)
______ Alcohol (2)
______ Caffeine (3)
______ smoked tobacco: cigarettes, cigars, hookah (4)
______ e-cigarettes/vapes (5)
______ marijuana (pot, weed), not prescribed (6)
______ medicinal marijuana, prescribed (7)
______ CBD (cannabidiol) (8)
______ synthetic marijuana (Serenity, Spice, K2) (9)
______ prescription opioids not prescribed to you (10)
______ opioids prescribed to you (11)
______ methadone not prescribed to you (12)
______ methadone prescribed to you (13)
______ Suboxone/Subutex (buprenorphine) not prescribed to you (14)
______ Suboxone/Subutex (buprenorphine) prescribed to you (15)
______ tianeptine not prescribed to you (16)
______ heroin (17)
______ fentanyl (18)
______ powder cocaine (19)
______ crack/rock/freebase cocaine (20)
______ street meth (crystal meth, crank) (21)
______ amphetamine pills (Ritalin, Adderall) not prescribed to you (22)
______ amphetamine pills (Ritalin, Adderall) prescribed to you (23)
______ Ecstasy/MDMA (24)
______ Modafinil/Provigil not prescribed to you (25)
______ DMT (26)
______ bath salts (synthetic cathinones) (27)
______ anti-anxiety drugs (Xanax, Valium, Ativan), not prescribed to you (28)
______ anti-anxiety drugs (Xanax, Valium, Ativan), prescribed to you (29)
______ hallucinogens (LSD, mushrooms) (30)
______ phenibut (31)
______ racetams (32)
______ DXM/dextromethorphan (33)
______ ketamine (34)
______ kava (35)
______ antidepressant medication prescribed to you (36)
______ antidepressant medication not prescribed to you (37)

© 2024 Smith KE et al. *JAMA Network Open.*

______ antipsychotic medication prescribed to you (38)
______ antipsychotic medication not prescribed to you (39)

Page Break

© 2024 Smith KE et al. *JAMA Network Open.*

X→

Q163 About how long has it been since ***your first time*** using kratom and **your *most recent*** time using kratom?

	**Year**	**Month**
**First Use:** (1)	▼ 1965 (1 ... 2022 (2022)	▼ January (1 ... Unsure (13)
**Most Recent Use:** (2)	▼ 1965 (1 ... 2022 (2022)	▼ January (1 ... Unsure (13)

Q164 Have you used kratom ***in the past month***?

○ Yes (1)

○ No (2)

Display This Question:
If used_k_last_month = No

Q165 Please describe the reasons you have not used kratom recently.

____
____
____
____
____

End of Block: Kratom and Other Drug Use History Questions

Start of Block: Motivations Subsection

Q172 Please select from the below list the ***most important factors*** that influence or motivate your kratom use. **Select all that apply.**

▢ Relieve short-term pain (acute pain management). (1)

▢ Self-treat long-term pain issues and symptoms (chronic pain management). (2)

▢ Relieve withdrawal symptoms from medically prescribed opioids. (3)

▢ Relieve withdrawal symptoms from nonprescribed opioids or heroin. (4)

▢ Relieve withdrawal symptoms from buprenorphine (Suboxone, Subutex). (5)

© 2024 Smith KE et al. *JAMA Network Open.*

- [ ] Relieve withdrawal symptoms from nonprescribed buprenorphine (Suboxone, Subutex). (6)
- [ ] Relieve withdrawal symptoms from nonprescribed methadone. (7)
- [ ] Relieve withdrawal symptoms from prescribed methadone. (8)
- [ ] Relieve alcohol withdrawal symptoms. (9)
- [ ] Relieve withdrawal symptoms from “nootropics”/ “noots”, “cognitive enhancing” supplements. (10)
- [ ] Relieve withdrawal symptoms from a variety of different drugs. (11)
- [ ] As a short-term substitute/replacement for opioids (e.g., prescription opioids, heroin). (12)
- [ ] As a long-term substitute/replacement for opioids (e.g., prescription opioids, heroin). (13)
- [ ] A short-term substitute/replacement for alcohol. (14)
- [ ] A long-term substitute/replacement for alcohol. (15)
- [ ] A short-term substitute/replacement for stimulants (meth, cocaine). (16)
- [ ] A long-term substitute/replacement for stimulants (meth, cocaine). (17)
- [ ] A short-term replacement for buprenorphine (Suboxone, Subutex). (18)
- [ ] A long-term replacement for buprenorphine (Suboxone, Subutex). (19)
- [ ] A short-term replacement for non-prescribed methadone. (20)
- [ ] A long-term replacement for prescribed methadone. (21)
- [ ] Address occasional feelings of sleepiness or low energy. (22)
- [ ] Self-treat chronic fatigue syndrome (23)
- [ ] Self-treat ADD/ADHD symptoms (24)
- [ ] As a study drug (25)
- [ ] Self-treat anxiety symptoms (26)

© 2024 Smith KE et al. *JAMA Network Open.*

- Reduce social anxiety (27)
- Self-treat bipolar symptoms (28)
- Self-treat depression symptoms (29)
- Self-treat post-traumatic stress symptoms (30)
- Self-treat headaches/migraines (31)
- Self-treat irritable bowel syndrome (32)
- As a sleep aid (33)
- Boost energy, stamina and/or endurance (for work, exercise) (34)
- Just to feel less crappy in general and improve your quality of life. (35)
- To take as part of a self-designed “stack” of other drugs that help you feel good. (36)
- For recreation, fun, or to relax. (37)
- To achieve a euphoric high. (38)
- Because you prefer the kratom “high” to “highs” you get from other drugs. (39)
- Couldn’t get a hold of other, more preferred drugs. (40)
- Kratom is safer than other substances. (41)
- Doctors won’t prescribe the drugs you need. (42)
- Difficulties obtaining buprenorphine (Suboxone, Subutex) or methadone. (43)
- Other (44) ____

Page Break

Carry Forward Selected Choices - Entered Text from "Please select from the below list the most important factors that influence or motivate your kratom use. Select all that apply."

Q173 Please select from the below list the ***number one*** ***reason motivating*** your kratom use during times when you were using it. **Select only one item.**

○ Relieve short-term pain (acute pain management). (1)

○ Self-treat long-term pain issues and symptoms (chronic pain management). (2)

○ Relieve withdrawal symptoms from medically prescribed opioids. (3)

○ Relieve withdrawal symptoms from nonprescribed opioids or heroin. (4)

○ Relieve withdrawal symptoms from buprenorphine (Suboxone, Subutex). (5)

○ Relieve withdrawal symptoms from nonprescribed buprenorphine (Suboxone, Subutex). (6)

○ Relieve withdrawal symptoms from nonprescribed methadone. (7)

○ Relieve withdrawal symptoms from prescribed methadone. (8)

○ Relieve alcohol withdrawal symptoms. (9)

○ Relieve withdrawal symptoms from “nootropics”/ “noots”, “cognitive enhancing” supplements. (10)

○ Relieve withdrawal symptoms from a variety of different drugs. (11)

○ As a short-term substitute/replacement for opioids (e.g., prescription opioids, heroin). (12)

○ As a long-term substitute/replacement for opioids (e.g., prescription opioids, heroin). (13)

○ A short-term substitute/replacement for alcohol. (14)

○ A long-term substitute/replacement for alcohol. (15)

○ A short-term substitute/replacement for stimulants (meth, cocaine). (16)

○ A long-term substitute/replacement for stimulants (meth, cocaine). (17)

○ A short-term replacement for buprenorphine (Suboxone, Subutex). (18)

○ A long-term replacement for buprenorphine (Suboxone, Subutex). (19)

○ A short-term replacement for non-prescribed methadone. (20)

○ A long-term replacement for prescribed methadone. (21)

© 2024 Smith KE et al. *JAMA Network Open.*

- ◯ Address occasional feelings of sleepiness or low energy. (22)
- ◯ Self-treat chronic fatigue syndrome (23)
- ◯ Self-treat ADD/ADHD symptoms (24)
- ◯ As a study drug (25)
- ◯ Self-treat anxiety symptoms (26)
- ◯ Reduce social anxiety (27)
- ◯ Self-treat bipolar symptoms (28)
- ◯ Self-treat depression symptoms (29)
- ◯ Self-treat post-traumatic stress symptoms (30)
- ◯ Self-treat headaches/migraines (31)
- ◯ Self-treat irritable bowel syndrome (32)
- ◯ As a sleep aid (33)
- ◯ Boost energy, stamina and/or endurance (for work, exercise) (34)
- ◯ Just to feel less crappy in general and improve your quality of life. (35)
- ◯ To take as part of a self-designed "stack" of other drugs that help you feel good. (36)
- ◯ For recreation, fun, or to relax. (37)
- ◯ To achieve a euphoric high. (38)
- ◯ Because you prefer the kratom "high" to "highs" you get from other drugs. (39)
- ◯ Couldn't get a hold of other, more preferred drugs. (40)
- ◯ Kratom is safer than other substances. (41)
- ◯ Doctors won't prescribe the drugs you need. (42)
- ◯ Difficulties obtaining buprenorphine (Suboxone, Subutex) or methadone. (43)
- ◯ Other (44)

Page Break

Carry Forward Selected Choices - Entered Text from "Please select from the below list the most important factors that influence or motivate your kratom use. Select all that apply."

X→

Q222 Typically, how effective was kratom for...

	**Not effective at all**	**Extremely effective**
	0	100
Relieve short-term pain (acute pain management). ()		
Self-treat long-term pain issues and symptoms (chronic pain management). ()		
Relieve withdrawal symptoms from medically prescribed opioids. ()		
Relieve withdrawal symptoms from nonprescribed opioids or heroin. ()		
Relieve withdrawal symptoms from buprenorphine (Suboxone, Subutex). ()		
Relieve withdrawal symptoms from nonprescribed buprenorphine (Suboxone, Subutex). ()		
Relieve withdrawal symptoms from nonprescribed methadone. ()		
Relieve withdrawal symptoms from prescribed methadone. ()		
Relieve alcohol withdrawal symptoms. ()		
Relieve withdrawal symptoms from “nootropics”/ “noots”, “cognitive enhancing” supplements. ()		
Relieve withdrawal symptoms from a variety of different drugs. ()		
As a short-term substitute/replacement for opioids (e.g., prescription opioids, heroin). ()		
As a long-term substitute/replacement for opioids (e.g., prescription opioids, heroin). ()		
A short-term substitute/replacement for alcohol. ()		
A long-term substitute/replacement for alcohol. ()		
A short-term substitute/replacement for stimulants (meth, cocaine). ()		

© 2024 Smith KE et al. *JAMA Network Open.*

A long-term substitute/replacement for stimulants (meth, cocaine). ()	
A short-term replacement for buprenorphine (Suboxone, Subutex). ()	
A long-term replacement for buprenorphine (Suboxone, Subutex). ()	
A short-term replacement for non-prescribed methadone. ()	
A long-term replacement for prescribed methadone. ()	
Address occasional feelings of sleepiness or low energy. ()	
Self-treat chronic fatigue syndrome ()	
Self-treat ADD/ADHD symptoms ()	
As a study drug ()	
Self-treat anxiety symptoms ()	
Reduce social anxiety ()	
Self-treat bipolar symptoms ()	
Self-treat depression symptoms ()	
Self-treat post-traumatic stress symptoms ()	
Self-treat headaches/migraines ()	
Self-treat irritable bowel syndrome ()	
As a sleep aid ()	
Boost energy, stamina and/or endurance (for work, exercise) ()	
Just to feel less crappy in general and improve your quality of life. ()	
To take as part of a self-designed “stack” of other drugs that help you feel good. ()	
For recreation, fun, or to relax. ()	
To achieve a euphoric high. ()	

© 2024 Smith KE et al. *JAMA Network Open.*

Because you prefer the kratom “high” to “highs” you get from other drugs. ()	
Couldn’t get a hold of other, more preferred drugs. ()	
Kratom is safer than other substances. ()	
Doctors won’t prescribe the drugs you need. ()	
Difficulties obtaining buprenorphine (Suboxone, Subutex) or methadone. ()	
Other ()	

Page Break

© 2024 Smith KE et al. *JAMA Network Open.*

Q223 Now thinking of how you generally take kratom, please indicate how true each item is:

	**Not at all** (1)	**A little** (2)	**Somewhat** (3)	**Quite a bit** (4)	**Extremely** (5)
I use kratom because other people do it. (1)	○	○	○	○	○
I use kratom because it feels nice. (2)	○	○	○	○	○
I use kratom to be creative. (3)	○	○	○	○	○
I use kratom to forget my problems. (4)	○	○	○	○	○
I use kratom to have fun at parties. (5)	○	○	○	○	○
I use kratom to relieve physical pain. (6)	○	○	○	○	○
I use kratom to be social. (7)	○	○	○	○	○
I use kratom so that others will accept me. (8)	○	○	○	○	○
I use kratom to be happy. (9)	○	○	○	○	○
I use kratom to cheer up when I feel sad. (10)	○	○	○	○	○
I use kratom so I don’t	○	○	○	○	○

© 2024 Smith KE et al. *JAMA Network Open.*

worry so much. (11)					
I use kratom so I can function with physical pain. (12)	○	○	○	○	○
I use kratom to expand my awareness. (13)	○	○	○	○	○
I use kratom so I won't be left out. (14)	○	○	○	○	○
I use kratom for energy. (15)	○	○	○	○	○
I use kratom to communicate with others better. (16)	○	○	○	○	○
I use kratom to get high. (17)	○	○	○	○	○
I use kratom to get off of heroin or opioids. (18)	○	○	○	○	○
I use kratom to see the world differently. (19)	○	○	○	○	○
I use kratom to feel better when I am upset. (20)	○	○	○	○	○
I use kratom to understand myself. (21)	○	○	○	○	○

© 2024 Smith KE et al. *JAMA Network Open.*

I use kratom to stop using other substances. (22)	○	○	○	○	○
I use kratom to lose weight. (23)	○	○	○	○	○
I use kratom to lift my mood. (24)	○	○	○	○	○
I use kratom to reduce social anxiety. (25)	○	○	○	○	○
I use kratom to make music more fun. (26)	○	○	○	○	○
I use kratom to focus my mind. (27)	○	○	○	○	○
I use kratom to stop withdrawal symptoms from other drugs. (28)	○	○	○	○	○

End of Block: Motivations Subsection

Start of Block: Do You Drive Block

X→

Q372 Do you drive a car?

○ Yes (1)

○ No (2)

End of Block: Do You Drive Block

Start of Block: Dose

Q218 When you take kratom, do you feel an effect ***pretty much every time***? These could be energy-boosting effects, like those of a cup of coffee, or intoxicating effects, like those of an alcoholic beverage—or anything else you feel from ***each dose***.

- ◯ Yes, feel an effect every time (or almost every time) I take kratom. (1)
- ◯ No, I never (or rarely) feel an effect when I take kratom. (2)
- ◯ Neither of those is quite true for me (please specify below) (3)

____

Display This Question:
If acute_effects = Yes, feel an effect every time (or almost every time) I take kratom.
Or acute_effects = Neither of those is quite true for me (please specify below)

Q219 If you do feel effects with ***each dose*** of kratom, are they primarily helpful in letting you go about your daily obligations?

- ◯ Yes, the effects are compatible with my daily obligations and help me achieve them (1)
- ◯ Yes, the effects are compatible with my daily obligations, though not especially helpful for them (2)
- ◯ No, the effects are not compatible with my daily obligations (3)
- ◯ No, the effects are not compatible with my daily obligations and they sometimes undermine my ability to meet my daily obligations (4)
- ◯ I don't take kratom enough to know (5)
- ◯ None of those are quite true for me (please specify below) (6)

____

Display This Question:
If acute_effects = Yes, feel an effect every time (or almost every time) I take kratom.
Or acute_effects = Neither of those is quite true for me (please specify below)

Q220 If you don't usually feel effects with ***each dose*** of kratom, which of these best describes why you use it?

- ◯ I don't want effects with each dose: I use kratom just for its long-term effects, the way some people use antidepressants or other medications. (1)
- ◯ I feel withdrawal symptoms if I stop using it. (2)
- ◯ Neither of those is quite true for me (please specify below) (3)

____

© 2024 Smith KE et al. *JAMA Network Open.*

Q221 What is the longest period (in weeks, months or years) during which you used kratom ***at least 3 times per week***? Please respond as accurately as possible.

____

Page Break

© 2024 Smith KE et al. *JAMA Network Open.*

Q256 Please select the types of kratom dosing units you typically use. **Select all that apply.**

- ▢ Grams (1)
- ▢ Spoonfuls (2)
- ▢ Teaspoons (3)
- ▢ Tablespoons (4)
- ▢ Capsules (standard size) (5)
- ▢ Capsules (jumbo size) (6)
- ▢ Cups of tea/juice (7)
- ▢ Extract (8)
- ▢ Edibles (9)
- ▢ Vape (measured in puffs) (10)
- ▢ Other (11) ____

Carry Forward Selected Choices - Entered Text from "Please select the types of kratom dosing units you typically use. Select all that apply."

Q258 For each of the kratom dosing units you selected above, please choose the typical amount that use during a given use event (e.g. each time you take it).

Grams (x1)	▼ 0.25 (1) ... more than 100 (128)
Spoonfuls (x2)	▼ 0.25 (1) ... more than 100 (128)
Teaspoons (x3)	▼ 0.25 (1) ... more than 100 (128)
Tablespoons (x4)	▼ 0.25 (1) ... more than 100 (128)
Capsules (standard size) (x5)	▼ 0.25 (1) ... more than 100 (128)
Capsules (jumbo size) (x6)	▼ 0.25 (1) ... more than 100 (128)
Cups of tea/juice (x7)	▼ 0.25 (1) ... more than 100 (128)
Extract (x8)	▼ 0.25 (1) ... more than 100 (128)

© 2024 Smith KE et al. *JAMA Network Open.*

Edibles (x9)	▼ 0.25 (1) ... more than 100 (128)
Vape (measured in puffs) (x10)	▼ 0.25 (1) ... more than 100 (128)
Other (x11)	▼ 0.25 (1) ... more than 100 (128)

Carry Forward Selected Choices - Entered Text from "Please select the types of kratom dosing units you typically use. Select all that apply."

X→

Q330 Please indicate which kratom dosing unit you use ***the most often***.

- ◯ Grams (1)
- ◯ Spoonfuls (2)
- ◯ Teaspoons (3)
- ◯ Tablespoons (4)
- ◯ Capsules (standard size) (5)
- ◯ Capsules (jumbo size) (6)
- ◯ Cups of tea/juice (7)
- ◯ Extract (8)
- ◯ Edibles (9)
- ◯ Vape (measured in puffs) (10)
- ◯ Other (11)

Q260 If your typical dose was not best described by one of the above options, please describe your typical dose:

____
____
____
____
____

Page Break

Q331 Please answer the following questions using the kratom product type you use the most often: ***${Q330/ChoiceGroup/SelectedChoices}***

Q280 What is the **lowest** kratom dose that you found to be **ineffective** or did not produce the desired results?
▼ 0.25 (0.25) ... more than 100 (101)

Q338 What is the **lowest** kratom dose that produced a **desired** effect for you?
▼ 0.25 (0.25) ... more than 100 (101)

Q339 What is the **highest** kratom dose that produced a **desired** effect for you (without being too much)?
▼ 0.25 (0.25) ... more than 100 (101)

Q340 What dose has been a bit **too much**, or produced results that weren't wanted or intended?
▼ 0.25 (0.25) ... more than 100 (101)

Page Break

Q261 Typically, how often would it take for you to ***begin*** feeling the effects of your usual dose of kratom?

- ◯ Seconds (1)
- ◯ Minutes (2)
- ◯ Hours (3)
- ◯ I don't know (4)

Q262 Typically, how often would it take for you to ***stop*** feeling the effects of your usual dose of kratom?

- ◯ Minutes (1)
- ◯ Hours (2)
- ◯ I'm unsure because I would take more kratom before the effects wear off (3)

Q263 Have you used kratom more than 100 times in your ***lifetime***?

- ◯ Yes (1)
- ◯ No (2)

Q264 How soon ***after you wake up*** do you use your first dose of kratom?

- ◯ Within 5 minutes (1)
- ◯ 6-30 minutes (2)
- ◯ 31-60 minutes (3)
- ◯ After 60 minutes (4)

Q265 Which kratom dose would you hate most to give up?

- ◯ The first one in the morning (1)
- ◯ All other times of day (2)

© 2024 Smith KE et al. *JAMA Network Open.*

Page Break

© 2024 Smith KE et al. *JAMA Network Open.*

Q333 Typical number of doses ***per day***:
▼ 1 (1) ... more than 50 (51)

Q269 How long have you been taking this many kratom doses ***per day***?

Please be as detailed as you like.

____
____
____
____
____

Q267 Do you take kratom more ***during the first hours after waking*** than during the rest of the day?

○ Yes (1)

○ No (2)

Q335 Typical number of doses ***per week***:
▼ 1 (1) ... more than 100 (101)

Q644 How long have you been taking this many kratom doses ***per week***?

Please be as detailed as you like.

____
____
____
____
____

Page Break

© 2024 Smith KE et al. *JAMA Network Open.*

Q278 Since you first began using kratom (***during times of active use***), how often did you change your dosing routine:

- Very often (1)
- Often (2)
- Occasionally (3)
- Not often (4)
- Never (5)
- It depended on other circumstances (6)

Q279 Since you first used kratom, your dosing/intake has:

- Significantly increased (1)
- Moderately increased (2)
- Increased a bit (3)
- Held steady and not changed (4)
- Decreased a bit (5)
- Moderately decreased (6)
- Significantly decreased (7)
- Never took it regularly enough for there to be much change (8)
- Not sure (9)
- I have completely stopped using kratom (10)
- Other (11) ____

Page Break

© 2024 Smith KE et al. *JAMA Network Open.*

Q285 Please select all of the effects that you have ever experienced within the ***minutes*** and hours after taking kratom:

- ☐ Pain relief (1)
- ☐ Sedation (2)
- ☐ Reduced craving for another drug (3)
- ☐ Reduced kratom craving (4)
- ☐ Reduced general anxiety (5)
- ☐ Reduced social anxiety (6)
- ☐ Euphoria (7)
- ☐ Improved mood (8)
- ☐ Increased energy (9)
- ☐ Increased alertness (10)
- ☐ Slower heart rate (11)
- ☐ Slower breathing/respiration (12)
- ☐ Sleepiness (13)
- ☐ Foggy memory (14)
- ☐ Increased focus (15)
- ☐ Desire to eat (16)
- ☐ Loss of appetite (17)
- ☐ Increased libido/sex drive (18)
- ☐ Increased productivity (19)

Display This Question:
If If keffects q://QID564/SelectedChoicesCount Is Greater Than 0

© 2024 Smith KE et al. *JAMA Network Open.*

Carry Forward Selected Choices from "Please select all of the effects that you have ever experienced within the minutes and hours after taking kratom:"

Q323 Please rate all of the effects that you have ever experienced within the minutes and hours after taking kratom:

	No Effect					Strong Effect					
	0	10	20	30	40	50	60	70	80	90	100
Pain relief ()											
Sedation ()											
Reduced craving for another drug ()											
Reduced kratom craving ()											
Reduced general anxiety ()											
Reduced social anxiety ()											
Euphoria ()											
Improved mood ()											
Increased energy ()											
Increased alertness ()											
Slower heart rate ()											
Slower breathing/respiration ()											
Sleepiness ()											
Foggy memory ()											
Increased focus ()											
Desire to eat ()											
Loss of appetite ()											
Increased libido/sex drive ()											

© 2024 Smith KE et al. *JAMA Network Open.*

Increased productivity ()	

Display This Question:
If If keffects q://QID564/SelectedChoicesCount Is Greater Than 0
Carry Forward Selected Choices from "Please select all of the effects that you have ever experienced within the minutes and hours after taking kratom:"

X→

Q324 Have you experienced this over weeks or months of regularly taking kratom?

	**Yes** (1)	**No** (2)	**Unsure** (3)
Pain relief (x7)	○	○	○
Sedation (x8)	○	○	○
Reduced craving for another drug (x9)	○	○	○
Reduced kratom craving (x10)	○	○	○
Reduced general anxiety (x11)	○	○	○
Reduced social anxiety (x12)	○	○	○
Euphoria (x13)	○	○	○
Improved mood (x14)	○	○	○
Increased energy (x15)	○	○	○
Increased alertness (x16)	○	○	○
Slower heart rate (x17)	○	○	○
Slower breathing/respiration (x18)	○	○	○
Sleepiness (x19)	○	○	○

Foggy memory (x20)	○	○	○
Increased focus (x21)	○	○	○
Desire to eat (x22)	○	○	○
Loss of appetite (x23)	○	○	○
Increased libido/sex drive (x24)	○	○	○
Increased productivity (x25)	○	○	○

Page Break

© 2024 Smith KE et al. *JAMA Network Open.*

Q286 Please score each of the following items according to how you felt when you stopped or couldn't use kratom for **a period of one day or longer**?

	**Not at all** (1)	**A little** (2)	**Moderately** (3)	**Quite a bit** (4)	**Extremely** (5)
I felt anxious. (1)	○	○	○	○	○
I felt like yawning. (2)	○	○	○	○	○
I was perspiring. (3)	○	○	○	○	○
My eyes were teary. (4)	○	○	○	○	○
My nose was running. (5)	○	○	○	○	○
I had goosebumps (6)	○	○	○	○	○
I was shaking. (7)	○	○	○	○	○
I had hot flushes. (8)	○	○	○	○	○
I had cold flushes. (9)	○	○	○	○	○
My bones and muscles ached. (10)	○	○	○	○	○
I felt restless. (11)	○	○	○	○	○
I felt nauseated. (12)	○	○	○	○	○
I felt like vomiting. (13)	○	○	○	○	○
My muscles twitched. (14)	○	○	○	○	○

© 2024 Smith KE et al. *JAMA Network Open.*

I had stomach cramps. (15)	○	○	○	○	○
I felt like using kratom. (16)	○	○	○	○	○
I felt like using another substance. (17)	○	○	○	○	○
I had restless legs. (18)	○	○	○	○	○
I had low energy and felt lethargic. (19)	○	○	○	○	○
I had fatigue. (20)	○	○	○	○	○
I felt irritable. (21)	○	○	○	○	○
I had difficulty sleeping. (22)	○	○	○	○	○
I felt depressed or sad. (23)	○	○	○	○	○

Page Break

© 2024 Smith KE et al. *JAMA Network Open.*

Q287 Please score each of the following items according to how you felt when you have missed ***a single regular dose*** of kratom?

	**Not at all** (1)	**A little** (2)	**Moderately** (3)	**Quite a bit** (4)	**Extremely** (5)
I felt anxious. (1)	○	○	○	○	○
I felt like yawning. (2)	○	○	○	○	○
I was perspiring. (3)	○	○	○	○	○
My eyes were teary. (4)	○	○	○	○	○
My nose was running. (5)	○	○	○	○	○
I had goosebumps (6)	○	○	○	○	○
I was shaking. (7)	○	○	○	○	○
I had hot flushes. (8)	○	○	○	○	○
I had cold flushes. (9)	○	○	○	○	○
My bones and muscles ached. (10)	○	○	○	○	○
I felt restless. (11)	○	○	○	○	○
I felt nauseated. (12)	○	○	○	○	○
I felt like vomiting. (13)	○	○	○	○	○
My muscles twitched. (14)	○	○	○	○	○

© 2024 Smith KE et al. *JAMA Network Open.*

I had stomach cramps. (15)	○	○	○	○	○
I felt like using kratom. (16)	○	○	○	○	○
I felt like using another substance. (17)	○	○	○	○	○
I had restless legs. (18)	○	○	○	○	○
I had low energy and felt lethargic. (19)	○	○	○	○	○
I had fatigue. (20)	○	○	○	○	○
I felt irritable. (21)	○	○	○	○	○
I had difficulty sleeping. (22)	○	○	○	○	○
I felt depressed or sad. (23)	○	○	○	○	○

Page Break

Q288 During times when you have been using kratom, where did you purchase kratom? **Select all that apply.**

- ☐ Gas station/convenience store (1)
- ☐ Head shop (2)
- ☐ Smoke shop (3)
- ☐ Online US-based vendor that I used previously and regularly (4)
- ☐ Online US-based vendor that I used previously but irregularly (5)
- ☐ Online vendor directly based in Malaysia that I used previously and regularly (6)
- ☐ Online vendor directly based in Malaysia that I used previously but irregularly (7)
- ☐ Online vendor directly based in Thailand that I used previously and regularly (8)
- ☐ Online vendor directly based in Thailand that I used previously but irregularly (9)
- ☐ Online vendor from unknown location (10)
- ☐ Herbal/vitamin shop (11)
- ☐ From a friend (12)
- ☐ High-end natural products store (13)
- ☐ From a store selling multiple products (e.g., CBD, vape) not otherwise classified. (14)
- ☐ Other: (15) ____

Q289 How often have you typically changed ***where*** you purchased kratom:

- ○ Very often (1)
- ○ Often (2)
- ○ Occasionally (3)
- ○ Not often (4)
- ○ Never (5)

© 2024 Smith KE et al. *JAMA Network Open.*

○ It depended on other circumstances (6)

Q290 During times when you have been using kratom, what brands or vendor did you find to be the ***most*** reliable and consistent in the quality of their products (e.g., consistency in potency/strength and the effects produced):

____

Q291 During times when you have been using kratom, what brands or vendor did you find to be the ***least*** reliable and consistent in the quality of their products (e.g., consistency in potency/strength and the effects produced):

____

Q292 What would you do if kratom became illegal to purchase or use?:

____

____

____

____

____

Page Break

© 2024 Smith KE et al. *JAMA Network Open.*

Q293 In what form did you typically purchase kratom? **Select all that apply.**

- ☐ Powder form (pulverized plant matter) (1)
- ☐ Leaf (loose, lightly crushed) form (2)
- ☐ Packaged capsules (3)
- ☐ Beverage freshly made (4)
- ☐ Prepackaged beverage (e.g., resembling energy drink, energy shots) (5)
- ☐ Kratom extracts (6)
- ☐ Edibles (7)
- ☐ Other: (8) ____

Q294 During times when you have been using kratom, what strains did you typically use? **Select all that apply.**

- ☐ Green (1)
- ☐ Red (2)
- ☐ White (3)
- ☐ Other (4)
- ☐ Not sure (5)
- ☐ I don't believe there are different strains (6)
- ☐ I've used based on vein leaf type, not strain (7)

Q341 Please describe the positive or negative feelings you received from the strains that you have used. Please be as detailed as you can.

____

____

____

© 2024 Smith KE et al. *JAMA Network Open.*

Q295 Have you ever participated in kratom advocacy or promotional activities? **Select all that apply.**

- ☐ I am a member of the American Kratom Association. (1)
- ☐ I subscribe to the American Kratom Association Newsletter. (2)
- ☐ I follow American Kratom Association associates or members on social media. (3)
- ☐ I have spoken at state or county town halls or legislative hearings on kratom scheduling/prohibitions. (4)
- ☐ I have written letters/emails to congressmen or other legislators on the legal status of kratom. (5)
- ☐ I have worked for shops that sell kratom. (6)
- ☐ I have worked for vendors, distributors, importers, or wholesalers of kratom. (7)
- ☐ I have made public comments on government websites (FDA docket). (8)
- ☐ I have signed a petition about kratom. (9)
- ☐ I have attended a meeting, protest, or gathering of kratom advocates. (10)
- ☐ Not sure. (11)
- ☐ Do not remember. (12)
- ☐ None of the above. (13)

Page Break

© 2024 Smith KE et al. *JAMA Network Open.*

Q296 These are the substances you said you've ever used ***within two hours*** of using kratom. Here we're asking which, if any, you found were especially good to use with kratom.

You could think of this like kratom being a complement to the other substance or vice versa, just like the way certain foods complement each other as part of a bigger meal. **Select all that apply.**

Carry Forward Selected Choices from "Kratom Co-Use Please select from the list below any substance you have ever used at the same time as kratom or within 1-2 hours of using kratom. Select all that apply."

Q297 Kratom went really well with and produced **better effects** with….

- ▢ None (-2)
- ▢ Other (40) ____
- ▢ None (-2)
- ▢ I don't remember (-1)
- ▢ Grapefruit juice (0)
- ▢ Kratom (1)
- ▢ Alcohol (2)
- ▢ Caffeine (3)
- ▢ smoked tobacco: cigarettes, cigars, hookah (4)
- ▢ e-cigarettes/vapes (5)
- ▢ marijuana (pot, weed), not prescribed (6)
- ▢ medicinal marijuana, prescribed (7)
- ▢ CBD (cannabidiol) (8)
- ▢ synthetic marijuana (Serenity, Spice, K2) (9)
- ▢ prescription opioids not prescribed to you (10)

© 2024 Smith KE et al. *JAMA Network Open.*

- opioids prescribed to you (11)
- methadone not prescribed to you (12)
- methadone prescribed to you (13)
- Suboxone/Subutex (buprenorphine) not prescribed to you (14)
- Suboxone/Subutex (buprenorphine) prescribed to you (15)
- tianeptine not prescribed to you (16)
- heroin (17)
- fentanyl (18)
- powder cocaine (19)
- crack/rock/freebase cocaine (20)
- street meth (crystal meth, crank) (21)
- amphetamine pills (Ritalin, Adderall) not prescribed to you (22)
- amphetamine pills (Ritalin, Adderall) prescribed to you (23)
- Ecstasy/MDMA (24)
- Modafinil/Provigil not prescribed to you (25)
- DMT (26)
- bath salts (synthetic cathinones) (27)
- anti-anxiety drugs (Xanax, Valium, Ativan), not prescribed to you (28)
- anti-anxiety drugs (Xanax, Valium, Ativan), prescribed to you (29)
- hallucinogens (LSD, mushrooms) (30)
- phenibut (31)
- racetams (32)
- DXM/dextromethorphan (33)

© 2024 Smith KE et al. *JAMA Network Open.*

- ☐ ketamine (34)
- ☐ kava (35)
- ☐ antidepressant medication prescribed to you (36)
- ☐ antidepressant medication not prescribed to you (37)
- ☐ antipsychotic medication prescribed to you (38)
- ☐ antipsychotic medication not prescribed to you (39)

Page Break

© 2024 Smith KE et al. *JAMA Network Open.*

Q298 How do you think of kratom? The list below has terms people could use to describe kratom or many other substances. Please select the terms that you would use to describe or conceptualize kratom. These can reflect both positive and negative aspects of kratom. There is no correct answer. **Select all that apply.**

- ▢ Habit-forming (1)
- ▢ Addictive (2)
- ▢ Medicinal (3)
- ▢ Therapeutic (4)
- ▢ Targeted by government agencies such as the FDA (5)
- ▢ Targeted by DEA for criminalization (6)
- ▢ Problematic (7)
- ▢ Helpful (8)
- ▢ Stigmatized (9)
- ▢ Energy shot (10)
- ▢ Should be legal (11)
- ▢ A real benefit to my daily life (12)
- ▢ Overhyped (13)
- ▢ Relaxing (14)
- ▢ Pre-workout (15)
- ▢ Study drug (16)
- ▢ Is an opioid (17)
- ▢ Life-saving (18)
- ▢ Boring (19)
- ▢ Potent (20)

© 2024 Smith KE et al. *JAMA Network Open.*

- ☐ Sometimes adulterated with other products (21)
- ☐ Not very potent (22)
- ☐ Isn’t an opioid (23)
- ☐ Stimulating (24)
- ☐ Media panic (25)
- ☐ Homeopathic (26)
- ☐ Inconsistent in its effects (27)
- ☐ Sedating (28)
- ☐ A combination of stimulating and sedating (29)
- ☐ Isn’t regulated across vendors who sell kratom (30)
- ☐ An increasing problem or burden for me (31)
- ☐ Too expensive (32)
- ☐ Naturopathic (33)
- ☐ Probably should be made illegal (34)
- ☐ Is not nearly as strong as opioids (35)
- ☐ Other (36) ____

Q299 Have these conceptualizations of kratom changed since you ***first*** heard about kratom?

- ○ Yes (1)
- ○ No (2)
- ○ I don't know (3)

Display This Question:
If have_thoughts_changed = Yes

© 2024 Smith KE et al. *JAMA Network Open.*

Q300 Overall, have your conceptualizations of kratom become more or less favorable?

- ◯ More favorable (1)
- ◯ Less favorable (2)
- ◯ It’s too complicated to say (3)
- ◯ I don’t know (4)
- ◯ Other: (5) ____

End of Block: Dose

Start of Block: Kratom Driving Section

Q224 Have you ever driven ***within 1-2 hours*** after taking kratom?

- ◯ Yes (1)
- ◯ No (2)
- ◯ I don't remember (3)

Display This Question:
If driven_within_hrs_k = Yes

Q225 How confident did you feel in your driving ability after using kratom?

	**Not at all confident**	**Extremely confident**
	0	100
()		

Q226 Have you ever driven ***within 2 hours*** after drinking alcohol?

- ◯ Yes (1)
- ◯ No (2)
- ◯ I don't remember (3)

Q227 Do you routinely or frequently drive on days you use kratom?

© 2024 Smith KE et al. *JAMA Network Open.*

○ Yes (1)

○ No (2)

○ Unsure (3)

Display This Question:
If routinely_drive_k = Yes

Q228 Do you believe that your driving abilities are about as good on days that you take kratom compared to days that you do not take kratom?

○ Yes (1)

○ No (2)

○ Unsure (3)

Q229 Please tell us why you responded this way about kratom and driving. There is no wrong answer.

____

____

____

____

____

End of Block: Kratom Driving Section

Start of Block: Driving Practice Questionnaire

Q230 Please select an appropriate answer for the following scenarios:

	**Never** (1)	**Rarely** (2)	**Sometimes** (3)	**Most of the time** (4)	**Always** (5)
Speed up when approaching a yellow light (1)	○	○	○	○	○
Drive 10 MPH or more over speed limit (2)	○	○	○	○	○

Take risks when driving (3)	○	○	○	○	○
Do things against the law if it’s safe (4)	○	○	○	○	○
Drive over speed limit in clear weather during the day (5)	○	○	○	○	○
Play music very loud while driving (6)	○	○	○	○	○
Drive over speed limit at night (7)	○	○	○	○	○
Eat or drink beverages while driving (8)	○	○	○	○	○
Drive on people’s bumpers (9)	○	○	○	○	○
Get back at other drivers with my car if they do something wrong (10)	○	○	○	○	○

Q231 Do you currently have a driver’s license (that is not suspended)?

○ Yes (1)

○ No (2)

© 2024 Smith KE et al. *JAMA Network Open.*

Q232 How long have you been driving ***(in years)***?
▼ 1 (1) ... 99 (99)

Q233 In an average week, how many ***days per week*** do you normally drive?
▼ 0 (8) ... 7 (15)

*

Q234 In an average week, how many ***total miles*** would you estimate you normally drive?
____

Q235 How would you rate your driving overall?

- ◯ Poor (1)
- ◯ Fair (2)
- ◯ Average (3)
- ◯ Good (4)
- ◯ Excellent (5)

Q236 How often do you wear a seatbelt when you drive?

- ◯ Never (1)
- ◯ Rarely (2)
- ◯ Most of the time (3)
- ◯ Always (4)

Q237 How often do you use your phone while driving to text message?

- ◯ Never (1)
- ◯ Rarely (2)
- ◯ Sometimes (6)
- ◯ Most of the time (3)

© 2024 Smith KE et al. *JAMA Network Open.*

○ Always (4)

*

Q238 How many accidents have you been involved in when you were the driver?

____

*

Q239 How many times have you been pulled over by the police, regardless of whether you received a ticket?

____

*

Q240 How many times have you been pulled over by the police and received a ticket?

____

Q241 Have you ever driven under the influence of alcohol (meaning you felt like you were drunk)?

○ Yes (1)

○ No (2)

Display This Question:
If dpq_ever_dui_alc = Yes

Q242 How many times ***in your lifetime*** have you driven under the influence of alcohol?

____

Display This Question:
If dpq_ever_dui_alc = Yes

*

Q243 How many times ***in the past year*** have you driven under the influence of alcohol?

____

© 2024 Smith KE et al. *JAMA Network Open.*

Display This Question:
If dpq_ever_dui_alc = Yes

Q244 Have you ever driven ***within two hours*** of drinking three to four alcoholic beverages? (The definition of a standard drink is 12 oz. of regular beer, 8 oz. of malt liquor, 5 oz. of wine, or 1.5 oz. of distilled spirits as a shot or as a mixed drink

○ Yes (1)

○ No (2)

Display This Question:
If dpq_ever_dui_alc = Yes

*

Q245 When you drive after drinking it is usually **(select all that apply)**:

▢ During the morning (6 am – 12 pm) (1)

▢ During the afternoon (12 pm – 6 pm) (2)

▢ During the evening (6 pm – 12 am) (3)

▢ During the early morning (12 am – 6 am) (4)

Q246 Have you ever driven under the influence of cannabis (meaning you felt like you were high)?

○ Yes (1)

○ No (2)

Display This Question:
If dpq_ever_dui_cannabis = Yes

Q247 How many times ***in your lifetime*** have you driven under the influence of cannabis?

____

Display This Question:
If dpq_ever_dui_cannabis = Yes

*

Q248 How many times ***in the past year*** have you driven under the influence of cannabis?

© 2024 Smith KE et al. *JAMA Network Open.*

Display This Question:
If dpq_ever_dui_cannabis = Yes

Q249 Have you ever driven ***within 1-2 hours*** of inhaling cannabis (smoking or vaping) or ***within 4-5 hours*** of orally ingesting cannabis?

- Yes (1)
- No (2)

Display This Question:
If dpq_ever_dui_cannabis = Yes

Q250 When you drive while feeling high, it is usually **(select all that apply)**:

- During the morning (6 am – 12 pm) (1)
- During the afternoon (12 pm – 6 pm) (2)
- During the evening (6 pm – 12 am) (3)
- During the early morning (12 am – 6 am) (4)

Q251 Have you ever driven under the influence of both cannabis and alcohol (meaning you were high and drunk, aka cross-faded)?

- Yes (1)
- No (2)

Display This Question:
If dpq_ever_dui_cross = Yes

Q252 How many times ***in your lifetime*** have you driven under the influence of both cannabis and alcohol?

© 2024 Smith KE et al. *JAMA Network Open.*

Display This Question:
If dpq_ever_dui_cross = Yes

Q253 How many times ***in the past year*** have you driven under the influence of both cannabis and alcohol?

____

Display This Question:
If dpq_ever_dui_cross = Yes

Q254 Have you ever driven ***within 1-4 hours*** of using cannabis products **AND** drinking 3-4 standard drinks? (The definition of a standard drink is 12 oz. of regular beer, 8 oz. of malt liquor, 5 oz. of wine, or 1.5 oz. of distilled spirits as a shot or as a mixed drink.)

- Yes (1)
- No (2)

Display This Question:
If dpq_ever_dui_cross = Yes

Q255 When you drive while cross-faded, it is usually **(select all that apply)**:

- During the morning (6 am – 12 pm) (1)
- During the afternoon (12 pm – 6 pm) (2)
- During the evening (6 pm – 12 am) (3)
- During the early morning (12 am – 6 am) (4)

End of Block: Driving Practice Questionnaire

Start of Block: SUBSTANCE USE DISORDER FOR KRATOM (KUD)

Q301 For the time period ***since you first ever*** used kratom, respond to each item below

	**Yes** (1)	**Yes, but not in the past year** (2)	**No** (0)
I used kratom in larger amounts and/or over a longer	○	○	○

© 2024 Smith KE et al. *JAMA Network Open.*

period than I had intended to. (1)			
I made at least one unsuccessful attempt to cut down or control my kratom use. (2)	○	○	○
I spent a great deal of time on activities necessary to get kratom, use the kratom, or recover from kratom's effects. (3)	○	○	○
I experienced cravings, strong desires, or urges for the kratom. (4)	○	○	○
My kratom use repeatedly interfered with my major role obligations (at work, school, or home). (5)	○	○	○
I kept using kratom despite knowing it was causing or worsening social or interpersonal problems for me. (6)	○	○	○
I gave up or reduced some important social, occupational or recreational activities because of my kratom use. (7)	○	○	○
I repeatedly used kratom in situations where it was physically hazardous. (8)	○	○	○
I kept using kratom despite knowing it was causing or worsening physical or	○	○	○

© 2024 Smith KE et al. *JAMA Network Open.*

psychological problems for me. (9)			
I needed to use larger amounts of kratom just to feel the same effect. (10)	○	○	○
I kept using the same amount of kratom, but didn't feel it as much. (11)	○	○	○
I had physical or psychological withdrawal symptoms during times I stopped using kratom. (12)	○	○	○
I kept using kratom in order to avoid withdrawal symptoms. (13)	○	○	○

Page Break

© 2024 Smith KE et al. *JAMA Network Open.*

Q302 How often have you attempted to quit using kratom ***after a regular period of use***?

- ○ Never (1)
- ○ At least once (2)
- ○ 2-5 times (3)
- ○ 5-10 times (4)
- ○ 10-15 times (5)
- ○ 20-25 times (6)
- ○ More than 25 times (7)
- ○ So many I can't recall (8)

Display This Question:
If quit_attempts != Never

Q303 Were you successful in your quit attempt?

- ○ Yes (1)
- ○ No (2)

Display This Question:
If quit_successful = No

Q304 Why do you believe you were not successful in your attempt to quit using kratom?

____
____
____
____
____

Page Break

© 2024 Smith KE et al. *JAMA Network Open.*

Q305 When using kratom, how open are you about your use? **Select all that apply.**

- ☐ I am completely out and open about it and won’t hide use from anyone (1)
- ☐ I completely hide my use to everyone I know (2)
- ☐ My significant other/romantic partner knows (3)
- ☐ Some, but not all, of my family knows (4)
- ☐ All of my family knows (5)
- ☐ Some, but not all, of my close friends know (6)
- ☐ All of my close friends know (7)
- ☐ Some of the people I work with or for know (8)
- ☐ All of the people I work with or for know (9)
- ☐ It is very mixed and I only talk about my use on a case by case basis (10)

Display This Question:
If open_about_k_use != I am completely out and open about it and won’t hide use from anyone

Q306 Are you this secretive or hidden about the use of any other substances?

- ○ Yes (1)
- ○ No (2)

Display This Question:
If secretive_abt_other = No

Q307 Please explain why you are more secretive, at least some of the time, with kratom use compared to the use of other substances:

____
____
____
____
____

© 2024 Smith KE et al. *JAMA Network Open.*

Q308 How comfortable would you feel disclosing current or prior kratom use to a medical practitioner?

- ○ Not at all comfortable (1)
- ○ A little uncomfortable (2)
- ○ Neither comfortable nor uncomfortable (3)
- ○ Pretty comfortable (4)
- ○ Very comfortable (5)
- ○ I have no reason to think about it one way or another because my use isn't a big deal (6)

Q309 Have you ***ever*** experienced what you perceive to be discrimination, stigma, or unfair treatment when seeking medical help in any of the following settings? While some of these places can be very similar, really try to think about which ones best apply to your own experiences, if any. **Select all that apply.**

- ▢ Emergency department (1)
- ▢ Urgent care clinic (2)
- ▢ Walk-in clinic (3)
- ▢ Health department (4)
- ▢ Community-based medical treatment center for physical health or pain (5)
- ▢ Community-based medical treatment center for mental health issues (6)
- ▢ Your regular family doctor or general practitioner (7)
- ▢ Private practice for physical health (8)
- ▢ Private practice for pain (9)
- ▢ Private practice for mental health (10)
- ▢ 12-Step meetings (11)
- ▢ Recovery-based, 12-step centers for substance use disorder treatment (inpatient or outpatient) (12)

- [ ] Inpatient treatment for substance use disorders (13)
- [ ] Outpatient treatment for substance use disorders (14)
- [ ] Methadone clinics (15)
- [ ] Medical doctors/clinics prescribing Suboxone/Subutex (16)
- [ ] Other: (17) ____

Page Break

Q310 During your ***lifetime***, how difficult has it generally been for you to get what you believe is adequate treatment for any ***mental health issues***?

- Extremely difficult (1)
- Difficult (2)
- Neither difficult nor easy (3)
- Easy (4)
- Extremely easy (5)
- Not applicable (6)

Display This Question:
If mental_health_treat_diff = Extremely difficult
Or mental_health_treat_diff = Difficult

Q311 Did this at all influence your decision to use kratom?

- Yes (1)
- No (2)
- Unsure (3)

Q312 During your ***lifetime***, how difficult has it generally been for you to get what you believe is adequate treatment for any ***acute or chronic pain issues***?

- Extremely difficult (1)
- Difficult (2)
- Neither difficult nor easy (3)
- Easy (4)
- Extremely easy (5)
- Not applicable (6)

Display This Question:
If pain_treat_diff = Extremely difficult
Or pain_treat_diff = Difficult

Q313 Did this at all influence your decision to use kratom?

© 2024 Smith KE et al. *JAMA Network Open.*

- Yes (1)
- No (2)
- Unsure (3)

Q314 During your ***lifetime***, how difficult has it generally been for you to get what you believe is adequate treatment for ***general physical health issues, not just those directly related to pain***?

- Extremely difficult (1)
- Difficult (2)
- Neither difficult nor easy (3)
- Easy (4)
- Extremely easy (5)
- Not applicable (6)

Display This Question:
If phys_health_treat_diff = Extremely difficult
Or phys_health_treat_diff = Difficult

Q315 Did this at all influence your decision to use kratom?

- Yes (1)
- No (2)
- Unsure (3)

Q316 During your ***lifetime***, how difficult has it generally been for you to get what you believe is adequate treatment for any ***issues with alcohol or other drug misuse (e.g., addiction, substance use disorder)***?

- Extremely difficult (1)
- Difficult (2)
- Neither difficult nor easy (3)
- Easy (4)

© 2024 Smith KE et al. *JAMA Network Open.*

○ Extremely easy (5)

○ Not applicable (6)

Display This Question:
If sud_treat_diff = Extremely difficult
Or sud_treat_diff = Difficult

Q317 Did this at all influence your decision to use kratom?

○ Yes (1)

○ No (2)

○ Unsure (3)

Page Break

© 2024 Smith KE et al. *JAMA Network Open.*

Q318 When you do use kratom, where do you typically use? **Select all that apply.**

- ☐ Home (1)
- ☐ Work (2)
- ☐ Vehicle (car, bus, train) (3)
- ☐ Waiting for ride, bus, etc. (4)
- ☐ Another's home (5)
- ☐ Outside in public places (6)
- ☐ Restaurant (7)
- ☐ Store (8)
- ☐ Church (9)
- ☐ Shelter (10)
- ☐ Bar / club (11)
- ☐ Clinic / doctor (12)
- ☐ Other: (13) ____

Q319 When you do use kratom, who is typically around you? **Select all that apply.**

- ☐ Spouse/Partner (1)
- ☐ Child(ren) (2)
- ☐ Other family (3)
- ☐ Coworkers (4)
- ☐ Friends (5)
- ☐ Acquaintances (6)
- ☐ Strangers (7)

© 2024 Smith KE et al. *JAMA Network Open.*

- [ ] Other: (8) ____

Q320 What activities are you engaged in when you typically use? **Select all that apply.**

- [ ] Resting/Sleeping (1)
- [ ] Working (2)
- [ ] Walking/riding/travelling (3)
- [ ] Eating or preparing food (4)
- [ ] Watching TV/movies/online videos (5)
- [ ] Listening to music (6)
- [ ] Talking on phone (7)
- [ ] Talking/Socializing (8)
- [ ] Arguing (9)
- [ ] Household chores or personal hygiene (10)
- [ ] Shopping/Errands (11)
- [ ] Child care/Elder care (12)
- [ ] Sports/Games (13)
- [ ] General recreation (14)
- [ ] Working out/exercising (15)
- [ ] Reading (16)
- [ ] Thinking/planning (17)
- [ ] Other: (18) ____

Q321 I typically want to use kratom when I am feeling…. **(select all that apply)**

© 2024 Smith KE et al. *JAMA Network Open.*

- ☐ Afraid (1)
- ☐ Angry (2)
- ☐ Annoyed (3)
- ☐ Exhausted (4)
- ☐ On Edge (5)
- ☐ Vigorous (6)
- ☐ Discouraged (7)
- ☐ Uneasy (8)
- ☐ Fatigued (9)
- ☐ Sad (10)
- ☐ Cheerful (11)
- ☐ Contented (12)
- ☐ Hopeless (13)
- ☐ Angry (14)
- ☐ Lively (15)
- ☐ Anxious (16)
- ☐ Resentful (17)
- ☐ Worn out (18)
- ☐ Relaxed (19)
- ☐ Bored (20)
- ☐ Lonely (21)

End of Block: SUBSTANCE USE DISORDER FOR KRATOM (KUD)

Start of Block: Demographics

© 2024 Smith KE et al. *JAMA Network Open.*

Q70 Which option best describes your employment ***for the majority of months out of the past 12 months***?

- ◯ Working full-time (1)
- ◯ Working part-time (2)
- ◯ Unemployed and looking for work (3)
- ◯ Unemployed and not looking for work (4)
- ◯ Disabled (5)
- ◯ Dealing drugs (6)
- ◯ Street hustling (7)
- ◯ Traded sex for work (8)
- ◯ Student (9)
- ◯ Retired (10)
- ◯ Incarcerated (11)

Q645 What is your profession?

____

Q71 How old are you **(in years)**?

____

JS

Q643 What is your date of birth?

Month (1)	▼ January (1) ... (123)
Day (2)	▼ January (1) ... (123)
Year (3)	▼ January (1) ... (123)

© 2024 Smith KE et al. *JAMA Network Open.*

Q72 What gender do you identify as?

- ◯ Male (1)
- ◯ Female (2)
- ◯ Nonbinary (3)
- ◯ Prefer not to say (4)

Q73 What zip code did you reside in for the majority of the time ***during the past year***? (We use this information to analyze how responses differ by geographical region, between urban/ suburban/rural areas. etc. We respect your privacy and we will not use this information for any kind of personal tracking.)

____

Q74 Education (check the ***highest*** schooling you have completed):

- ◯ 8th grade or less (1)
- ◯ 9-12th grade, but didn't finish high school (2)
- ◯ finished high school or got GED (3)
- ◯ some college (4)
- ◯ Associates/Vocational Degree (5)
- ◯ Bachelor's Degree (6)
- ◯ Master's Degree (7)
- ◯ Ph.D. (8)
- ◯ J.D. (9)
- ◯ M.D. (10)

Q77 What is your sexual orientation?

- ◯ Heterosexual/Straight (1)

© 2024 Smith KE et al. *JAMA Network Open.*

- Gay (2)
- Lesbian (3)
- Bisexual (4)
- Asexual (5)
- Queer (6)
- Don’t Know (7)
- Prefer not to say (8)
- Other (9) ____

X→

Q78 Race/Ethnicity **(select all that apply)**:

- ▢ Asian (1)
- ▢ Black/African American (2)
- ▢ Hispanic (3)
- ▢ Indian (4)
- ▢ Middle-Eastern (5)
- ▢ Native American, including native Alaskan or Pacific Islander (6)
- ▢ White/European American (7)
- ▢ Biracial (8)
- ▢ Multiracial (9)
- ▢ Other: (10) ____

Page Break

Q79 How tall are you?

Q641 Feet?
▼ 4 (4) ... 7 (7)

Q642 Inches?
▼ 0 (0) ... 12 (12)

Q80 Relationship status (select all that apply):

- ☐ Never married (1)
- ☐ Currently Married (2)
- ☐ Widowed (3)
- ☐ Divorced (4)
- ☐ In committed partner relationship, monogamous (5)
- ☐ In committed partner relationship, polyamorous (6)
- ☐ Single and looking for a long-term partner (7)
- ☐ Single and looking only for short-term hook-ups and sex (8)
- ☐ In a committed relationship or marriage, but also cheating on the side (9)

Q81 Do you have any children?

- ○ Yes (1)
- ○ No (2)

Display This Question:

© 2024 Smith KE et al. *JAMA Network Open.*

If demo_children = Yes

*

Q84 How many still living ***over*** the age of 18?

____

Display This Question:
If demo_children = Yes

*

Q85 How many still living ***under*** the age of 18?

____

*

Q87 Counting all sources of income, what was your income for the past month (in dollars)?

____

*

Q88 Counting all sources of income, what was your average annual income for past year (in dollars)?

____

Q89 Have you ***ever*** been incarcerated in jail or prison?

- ○ Yes (1)
- ○ No (2)

Q90 Are you ***currently*** on probation, parole, or house arrest?

- ○ Yes (1)
- ○ No (2)

End of Block: Demographics

Start of Block: History of problematic drug use, SUDs, and treatment

Q91 Below are some additional questions related to drug use. Remember, all responses are completely anonymous and confidential.

Carry Forward Unselected Choices from "Total Number of Months Used"

Q93 Of all of the substances (including alcohol and tobacco) that you've used in ***the past 12 months <u>other than kratom</u>***, which one would you have used the ***most often***, more than other substances?
▼ Didn't use any other substances in the past 12 months (1) ... antipsychotic medication not prescribed to you (40)

Carry Forward Displayed Choices from "Of all of the substances (including alcohol and tobacco) that you've used in the past 12 months other than kratom, which one would you have used the most often, more than other substances?"

Q94 Of all of the drugs (including alcohol and tobacco) that you've used in ***the past 12 months***, which one would you say you have the ***biggest problem*** with?
▼ No problems with any of the substances I used in the past 12 months (0) ... antipsychotic medication not prescribed to you (39)

Display This Question:
If past12drugs_biggest_prob = No problems with any of the substances I used in the past 12 months

Q143 For the drug that you used ***most frequently in the past 12 months***, **${Q93/ChoiceGroup/SelectedChoices}**, please respond to the following statements about it.

Display This Question:
If past12drugs_biggest_prob = No problems with any of the substances I used in the past 12 months

Q98 **In the past 12 months....**

	**Yes** (1)	**No** (0)
I used in larger amounts and/or over a longer period than I had intended to. (1)	○	○

© 2024 Smith KE et al. *JAMA Network Open.*

I made at least one unsuccessful attempt to cut down or control my use. (2)	○	○
I spent a great deal of time on activities necessary to get the drug, use the drug, or recover from its effects. (3)	○	○
I experienced cravings, strong desires, or urges for the drug. (4)	○	○
My use repeatedly interfered with my major role obligations (at work, school, or home). (5)	○	○
I kept using despite knowing it was causing or worsening social or interpersonal problems for me. (6)	○	○
I gave up or reduced some important social, occupational or recreational activities because of my use. (7)	○	○
I repeatedly used in situations where it was physically hazardous. (8)	○	○
I kept using despite knowing it was causing or worsening physical or psychological problems for me. (9)	○	○
I needed to use larger amounts just to feel the same effect. (10)	○	○
I kept using the same amount, but didn't feel it as much. (11)	○	○
I had physical or psychological withdrawal symptoms during times I stopped using. (12)	○	○
I kept using in order to avoid withdrawal symptoms. (13)	○	○

© 2024 Smith KE et al. *JAMA Network Open.*

Page Break

© 2024 Smith KE et al. *JAMA Network Open.*

Q99 Have you ***ever*** been diagnosed with (or told by a medical professional that you have) a substance use disorder or addiction to any drug, including alcohol?

○ Yes (1)

○ No (2)

○ I don’t know (3)

Q100 Do *you* believe you ***ever*** had a substance use disorder or addiction (including alcoholism)?

○ Never (1)

○ A long time ago, but not now (2)

○ Recently, but not now (3)

○ Currently (4)

Q101 ***In the past 12 months***, how many ***months*** were you actively enrolled in any substance use treatment program, including any program you may be in now? This could include inpatient, outpatient, residential recovery center, methadone or buprenorphine treatment, etc., but it does ***not*** include peer-led groups like Alcoholics Anonymous unless they’re part of your formal treatment.
▼ I haven't been in any treatment program in the past 12 months (-2) ... 12 months (12)

Q102 Have you ***ever*** been enrolled in a methadone program or clinic?

○ Yes, previously (1)

○ Yes, currently (2)

○ Never (3)

Q103 Have you ***ever*** been enrolled in a program that prescribes buprenorphine (Suboxone, Subutex)?

○ Yes, previously (1)

○ Yes, currently (2)

© 2024 Smith KE et al. *JAMA Network Open.*

○ Never (3)

Q104 Do you ***currently*** consider yourself to be in recovery from substance use?

○ Yes (1)

○ No (2)

Display This Question:
If consider_in_recovery = No

Q105 How much interest do you have ***right now*** in pursuing recovery from substance use?

○ I have no interest whatsoever (0)

○ I'm a little interested (1)

○ I'm somewhat interested (2)

○ I'm really interested (3)

○ I'm extremely interested (4)

End of Block: History of problematic drug use, SUDs, and treatment

Start of Block: Perceived Stigma of Substance Use

Q106 Please rate how strongly you agree with each of the following statements.

	**Strongly Disagree** (1)	**Disagree** (2)	**Agree** (3)	**Strongly Agree** (4)
Most people would willingly accept someone who has had substance use problems or who has been treated for their substance use as a close friend. (1)	○	○	○	○

Most people believe that someone who has had substance use problems or who has been treated for substance use is just as trustworthy as the average citizen. (2)	○	○	○	○
Most people would accept someone who has had substance use problems or who has been treated for substance use as a teacher of young children in a public school. (3)	○	○	○	○
Most people would hire someone who has had substance use problems or who has been treated for substance use to take care of their children. (4)	○	○	○	○
Most people think less of a person who has had substance use problems or who has been in treatment for substance use. (5)	○	○	○	○

© 2024 Smith KE et al. *JAMA Network Open.*

Most employers will hire someone who has had substance use problems or who has been treated for substance use if he or she is qualified for the job. (6)	○	○	○	○
Most employers will pass over the application of someone who has had substance use problems or who has been treated for their substance use in favor of another applicant. (7)	○	○	○	○
Most people would be willing to date someone who has had substance use problems, or who has been treated for their substance use. (8)	○	○	○	○

End of Block: Perceived Stigma of Substance Use

Start of Block: Perceived Socioeconomic Status

Q107 Think of this ladder as representing where people stand in our society. At the ***top*** of the ladder are the people who are who are best off, have the most money, most education and best jobs. At the ***bottom*** are the people who are the worst off, those who have the least money, least education, and worst jobs or no job. The higher up you are on this ladder, the closer you are to people at the very top and the lower you are, the closer you are to the bottom.

© 2024 Smith KE et al. *JAMA Network Open.*

Q108

Q109 Please select ***the rung where you think you started out in childhood***. In other words, what rung do you think you were born into, compared to other people?

	Bottom Rung					Top Rung				
	1	2	3	4	5	6	7	8	9	10
3 ()										

Q110 Please select ***the rung where you think you stand now***. In other words, where would you put yourself on the ladder today compared to other people?

	Bottom Rung					Top Rung				
	1	2	3	4	5	6	7	8	9	10
1 ()										

Q111 Now, consider ***the rung where you might be in the future***. In other words, do you expect to go further down or further up on this ladder?

	Bottom Rung					Top Rung				
	1	2	3	4	5	6	7	8	9	10
1 ()										

End of Block: Perceived Socioeconomic Status

Start of Block: ADHD Assessment

Q114 For each statement below, please select the frequency that best describes your behavior when you were a ***child between 5 and 12 years of age***.

© 2024 Smith KE et al. *JAMA Network Open.*

	**Never or rarely** (1)	**Sometimes** (2)	**Often** (3)	**Very Often** (4)
Failed to give close attention to details or made careless mistakes in my work or other activities (1)	○	○	○	○
Had difficulty sustaining my attention in tasks or fun activities (2)	○	○	○	○
Didn’t listen when spoken to directly (3)	○	○	○	○
Didn’t follow through on instructions and failed to finish work or chores. (4)	○	○	○	○
Had difficulty organizing tasks and activities (5)	○	○	○	○
Avoided, disliked, or was reluctant to engage in tasks that required sustained mental effort (6)	○	○	○	○
Lost things necessary for tasks or activities (7)	○	○	○	○
Was easily distracted by extraneous stimuli or irrelevant thoughts (8)	○	○	○	○

© 2024 Smith KE et al. *JAMA Network Open.*

Was forgetful in daily activities (9)	○	○	○	○
Fidgeted with hands or feet or squirmed in seat (10)	○	○	○	○
Left my seat in classrooms or in other situations in which remaining seated was expected (11)	○	○	○	○
Shifted around excessively or felt restless or hemmed in (12)	○	○	○	○
Had difficulty engaging in leisure activities quietly (felt uncomfortable, or was loud or noisy) (13)	○	○	○	○
Was “on the go” or acted as if “driven by a motor” (14)	○	○	○	○
Talked excessively (15)	○	○	○	○
Blurted out answers before questions had been completed, completed others’ sentences, or jumped the gun (16)	○	○	○	○
Had difficulty awaiting my turn (17)	○	○	○	○

© 2024 Smith KE et al. *JAMA Network Open.*

Interrupted or intruded on others (butted into conversations or activities without permission or took over what others were doing) (18)	○	○	○	○

Q115 Did you experience ***any*** of these symptoms at least "Often" or more frequently?

○ Yes (1)

○ No (2)

Display This Question:
If adhd_experienced_symptoms_often = Yes

Q116 In which of these settings did those symptoms impair your functioning? **Select all that apply.**

▢ School (1)

▢ Home (2)

▢ Social Relationships (3)

▢ Don't Remember (4)

X→

Q124 For each statement below, please select the option that best describes your behavior ***for the past 6 months.***

	**Yes** (1)	**No** (0)
I often fail to give close attention to details or make careless mistakes in schoolwork, at work, or during other activities (e.g.,	○	○

© 2024 Smith KE et al. *JAMA Network Open.*

overlooks or misses details; work is inaccurate). (1)		
I often have difficulty sustaining attention in tasks or play activities (e.g., has difficulty remaining focused during lectures, conversations, or lengthy reading). (2)	○	○
I often do not seem to listen when spoken to directly (e.g., mind seems elsewhere, even in the absence of any obvious distraction). (3)	○	○
I often do not follow through on instructions and fail to finish schoolwork, chores, or duties in the workplace (e.g., starts tasks but quickly loses focus and is often sidetracked). (4)	○	○
I often have difficulty organizing tasks and activities (e.g., difficulty managing sequential tasks; difficulty keeping belongings in order; messy, disorganized work; poor time management; fails to meet deadlines). (5)	○	○
I often avoid, dislike or am reluctant to engage in tasks that require sustained mental effort (e.g., schoolwork, preparing reports, completing forms, reviewing lengthy papers). (6)	○	○
I often lose things necessary for tasks or activities (e.g., school materials; tools; wallet; keys; paperwork, glasses; cell phone). (7)	○	○

© 2024 Smith KE et al. *JAMA Network Open.*

I'm often easily distracted by extraneous stimuli or unrelated thoughts. (8)	○	○
I'm often forgetful in daily activities (e.g., doing chores, running errands, returning calls, paying bills, keeping appointments). (9)	○	○
I often fidget or tap hands or feet; often squirm in my seat. (10)	○	○
I often leave my seat in situations when remaining seated is expected (e.g., leaves my place in class, the office, workplace settings, or other situations which require I remain seated in place). (11)	○	○
I often move about in situations where it is inappropriate, feeling restlessness. (12)	○	○
I often am unable to engage in leisure activities quietly. (13)	○	○
I am often "on the go", acting as if I'm "driving by a motor" (e.g., unable to be or uncomfortable being still for extended time such as in restaurants, meetings; "others say they can't keep up with me"). (14)	○	○
I often talk excessively. (15)	○	○
I often blurt out answers or responses before a question has been completed (e.g., complete people's sentences; cannot wait for my turn in conversation). (16)	○	○

© 2024 Smith KE et al. *JAMA Network Open.*

I often have difficulty waiting my turn (e.g., waiting in line). (17)	○	○
I often interrupt or intrude on others (e.g., butt into conversations or activities; I may start to use people's things without asking or permission; take over what others are doing). (18)	○	○

End of Block: ADHD Assessment

Start of Block: Perceived Stress Scale

Q141 These questions ask about your feelings and thoughts during the last month. Please select the item reflecting how often you felt or thought a certain way.

	**Never** (0)	**Almost Never** (1)	**Sometimes** (2)	**Fairly Often** (3)	**Very Often** (4)
In the last month, how often were you upset because of something that happened unexpectedly? (1)	○	○	○	○	○
In the last month, how often did you feel unable to control important things in your life? (2)	○	○	○	○	○
In the last month, how often did you feel nervous or stressed? (3)	○	○	○	○	○

© 2024 Smith KE et al. *JAMA Network Open.*

In the last month, how often did you deal successfully with irritating life hassles? (4)	○	○	○	○	○
In the last month, how often did you feel that you were effectively coping with important changes that are/were occurring in your life? (5)	○	○	○	○	○
In the last month, how often did you feel confident about your ability to handle your personal problems? (6)	○	○	○	○	○
In the last month, how often did you feel things were going your way? (7)	○	○	○	○	○
In the last month, how often did you find that you could not cope with all the things that you had to do? (8)	○	○	○	○	○

© 2024 Smith KE et al. *JAMA Network Open.*

In the last month, how often were you able to control the irritations in your life? (9)	○	○	○	○	○
In the last month, how often did you feel that you were on top of things? (10)	○	○	○	○	○
In the last month, how often were you angered because of things that happened that were outside of your control? (11)	○	○	○	○	○
In the last month, how often did you find yourself thinking about things that you have to accomplish? (12)	○	○	○	○	○
In the last month, how often were you able to control the way you spent your time? (13)	○	○	○	○	○
In the last month, how often did you feel that difficulties	○	○	○	○	○

© 2024 Smith KE et al. *JAMA Network Open.*

were piling up so high that you could not overcome them? (14)	

End of Block: Perceived Stress Scale

Start of Block: Depression Scale

Q144 Please indicate how often you have felt this way during the ***past 30 days*** by checking the appropriate box for each question.

Q142 ***In the last 30 days….***

	**Rarely or none of the time** (0)	**Some or a little of the time** (1)	**Occasionally or a moderate amount of time** (2)	**All of the time** (3)
I was bothered by things that usually don't bother me. (1)	○	○	○	○
I had trouble keeping my mind on what I was doing. (2)	○	○	○	○
I felt depressed. (3)	○	○	○	○
I felt that everything I did was an effort. (4)	○	○	○	○
I felt hopeful about the future. (5)	○	○	○	○
I felt fearful. (6)	○	○	○	○
My sleep was restless. (7)	○	○	○	○

I was happy. (8)	○	○	○	○
I felt lonely. (9)	○	○	○	○

End of Block: Depression Scale

Start of Block: Generalized Anxiety Scale

Q146 Over the ***past 30 days***, how often have you been bothered by the following problems?

X→

Q145 ***In the past 30 days….***

	**Not at all** (0)	**Several days** (1)	**Over half the time (more than 15 days)** (2)	**Nearly every day** (3)
Feeling nervous, anxious, or on edge. (1)	○	○	○	○
Not being able to stop or control worrying. (2)	○	○	○	○
Worrying too much about different things. (3)	○	○	○	○
Trouble relaxing. (4)	○	○	○	○
Being so restless that it's hard to sit still. (5)	○	○	○	○
Becoming easily annoyed or irritable. (6)	○	○	○	○
Feeling afraid as if something awful might happen. (7)	○	○	○	○

© 2024 Smith KE et al. *JAMA Network Open.*

Display This Question:
If gas_last30days [Not at all] (Count) != 7

Q147 If you checked off any problems, how difficult have these made it for you to do your work, take care of things at home, or get along with other people?

- Not all difficult (1)
- Somewhat difficult (2)
- Very difficult (3)
- Extremely difficult (4)

End of Block: Generalized Anxiety Scale

Start of Block: Life Satisfaction

Q148 Below are several questions about general satisfaction. Please reflect on each before selecting your response.

	**Strongly disagree** (1)	**Disagree** (2)	**Slightly disagree** (3)	**Neither agree nor disagree** (4)	**Slightly Agree** (5)	**Agree** (6)	**Strongly agree** (7)
In most ways my life is close to my ideal. (1)	○	○	○	○	○	○	○
The conditions of my life are excellent. (2)	○	○	○	○	○	○	○
I am satisfied with my life. (3)	○	○	○	○	○	○	○

© 2024 Smith KE et al. *JAMA Network Open.*

So far, I have gotten the important things I want in life. (4)	○	○	○	○	○	○	○
If I could live my life over, I would change almost nothing. (5)	○	○	○	○	○	○	○

X→

Q158 Click to write the question text

	**Very dissatisfying** (1)	**Dissatisfying** (2)	**Rather dissatisfying** (3)	**Rather satisfying** (4)	**Satisfying** (5)	**Very satisfying** (6)
Life as a whole is… (1)	○	○	○	○	○	○
My vocational situation is… (2)	○	○	○	○	○	○
My financial situation is... (3)	○	○	○	○	○	○
My leisure situation is… (4)	○	○	○	○	○	○
My contacts with friends and acquaintances are… (5)	○	○	○	○	○	○

© 2024 Smith KE et al. *JAMA Network Open.*

My sexual life is… (6)	○	○	○	○	○	○
My ability to manage my self-care is… (7)	○	○	○	○	○	○
My family life is… (8)	○	○	○	○	○	○
My partnership relation is… (9)	○	○	○	○	○	○
My physical health is… (10)	○	○	○	○	○	○
My psychological health is… (11)	○	○	○	○	○	○

End of Block: Life Satisfaction

Start of Block: Chronic Pain

Q159 Throughout our lives, most of us have had pain from time to time (such as minor headaches, sprains, and toothaches). Have you had pain other than these everyday kinds of pain ***today***?

○ Yes (1)

○ No (2)

Display This Question:
If cp_ever_had = Yes

Q160 Have you felt this pain for ***at least 3 months***?

○ Yes (1)

○ No (2)

Display This Question:
If cp_atleast_3_months = Yes

© 2024 Smith KE et al. *JAMA Network Open.*

Q161 Please rate your pain by indicating the number that best describes your pain, with **0** being **No Pain** and **10** being **Pain as Bad as You Can Imagine**

	**No Pain**	**As Bad as You Can Imagine**
	0 1 2 3 4 5 6 7 8 9 10	
At its **WORST** in the past 24 hours ()		
At its **LEAST** in the past 24 hours ()		
**ON AVERAGE** in the past 24 hours ()		
**RIGHT NOW** ()		

Page Break

© 2024 Smith KE et al. *JAMA Network Open.*

Display This Question:
If cp_atleast_3_months = Yes

Q162 Please indicate any areas where you feel pain. **Select all that apply.**

- ☐ Front of head (1)
- ☐ Front of neck (2)
- ☐ Front of upper torso (chest) (3)
- ☐ Front of right upper arm (4)
- ☐ Front of right elbow (5)
- ☐ Front of right forearm (6)
- ☐ Front of right wrist (7)
- ☐ Front of right hand (8)
- ☐ Front of left upper arm (9)
- ☐ Front of left elbow (10)
- ☐ Front of left forearm (11)
- ☐ Front of left wrist (12)
- ☐ Front of left hand (13)
- ☐ Front of lower torso (stomach) (14)
- ☐ Front of pelvic area/hips (15)
- ☐ Front of right thigh (16)
- ☐ Front of right knee (17)
- ☐ Front of right calf (18)
- ☐ Front of right ankle (19)
- ☐ Front of right foot (20)
- ☐ Front of left thigh (21)

© 2024 Smith KE et al. *JAMA Network Open.*

- [ ] Front of left knee (22)
- [ ] Front of left calf (23)
- [ ] Front of left ankle (24)
- [ ] Front of left foot (25)
- [ ] Back of head (26)
- [ ] Back of neck (27)
- [ ] Back of upper torso (chest) (28)
- [ ] Back of right upper arm (29)
- [ ] Back of right elbow (30)
- [ ] Back of right forearm (31)
- [ ] Back of right wrist (32)
- [ ] Back of right hand (33)
- [ ] Back of left upper arm (34)
- [ ] Back of left elbow (35)
- [ ] Back of left forearm (36)
- [ ] Back of left wrist (37)
- [ ] Back of left hand (38)
- [ ] Back of lower torso (stomach) (39)
- [ ] Back of pelvic area/hips (40)
- [ ] Back of right thigh (41)
- [ ] Back of right knee (42)
- [ ] Back of right calf (43)
- [ ] Back of right ankle (44)

© 2024 Smith KE et al. *JAMA Network Open.*

- Back of right foot (45)
- Back of left thigh (46)
- Back of left knee (47)
- Back of left calf (48)
- Back of left ankle (49)
- Back of left foot (50)

Display This Question:
If If Please indicate any areas where you feel pain. Select all that apply. q://QID463/SelectedChoicesCount Is Greater Than 0
Carry Forward Selected Choices from "Please indicate any areas where you feel pain. Select all that apply."

X→

Q163 Which of these areas hurts the ***most***?

- Front of head (1)
- Front of neck (2)
- Front of upper torso (chest) (3)
- Front of right upper arm (4)
- Front of right elbow (5)
- Front of right forearm (6)
- Front of right wrist (7)
- Front of right hand (8)
- Front of left upper arm (9)
- Front of left elbow (10)
- Front of left forearm (11)
- Front of left wrist (12)
- Front of left hand (13)
- Front of lower torso (stomach) (14)

- Front of pelvic area/hips (15)
- Front of right thigh (16)
- Front of right knee (17)
- Front of right calf (18)
- Front of right ankle (19)
- Front of right foot (20)
- Front of left thigh (21)
- Front of left knee (22)
- Front of left calf (23)
- Front of left ankle (24)
- Front of left foot (25)
- Back of head (26)
- Back of neck (27)
- Back of upper torso (chest) (28)
- Back of right upper arm (29)
- Back of right elbow (30)
- Back of right forearm (31)
- Back of right wrist (32)
- Back of right hand (33)
- Back of left upper arm (34)
- Back of left elbow (35)
- Back of left forearm (36)
- Back of left wrist (37)
- Back of left hand (38)
- Back of lower torso (stomach) (39)
- Back of pelvic area/hips (40)
- Back of right thigh (41)

© 2024 Smith KE et al. *JAMA Network Open.*

- ○ Back of right knee (42)
- ○ Back of right calf (43)
- ○ Back of right ankle (44)
- ○ Back of right foot (45)
- ○ Back of left thigh (46)
- ○ Back of left knee (47)
- ○ Back of left calf (48)
- ○ Back of left ankle (49)
- ○ Back of left foot (50)

Page Break

© 2024 Smith KE et al. *JAMA Network Open.*

Display This Question:
If cp_atleast_3_months = Yes

Q174 What kinds of things make your pain feel better? **Select all that apply.**

- ▢ Heat (1)
- ▢ Medicine (2)
- ▢ Rest (3)
- ▢ Other (4) ____
- ▢ ⊗None of these make it better (5)

Display This Question:
If cp_atleast_3_months = Yes

Q175 What kinds of things make your pain feel ***worse***? **Select all that apply.**

- ▢ Walking (1)
- ▢ Standing (2)
- ▢ Lifting (3)
- ▢ Other (4) ____
- ▢ ⊗None of these make it worse (5)

Display This Question:
If cp_atleast_3_months = Yes

Q176 What treatments or medications are you receiving for pain?
____

Display This Question:
If cp_atleast_3_months = Yes

Q177 ***In the last week***, how much relief have pain treatments or medications provided? Please indicate the percentage that shows how much relief you have received. If you do not receive any treatments or medications for your pain, select **N**ot Applicable.

**No Relief**	**Complete Relief**	Not Applicable

© 2024 Smith KE et al. *JAMA Network Open.*

	0 10 20 30 40 50 60 70 80 90 100
1 ()	

Display This Question:
If cp_atleast_3_months = Yes

Q178 If you take pain medication, how many ***hours*** does it take before the pain returns?

- ◯ Pain medication doesn’t help at all (1)
- ◯ One hour (2)
- ◯ Two hours (3)
- ◯ Three hours (4)
- ◯ Four hours (5)
- ◯ Five to twelve hours (6)
- ◯ More than twelve hours (7)
- ◯ I do not take pain medication (8)

Page Break

Display This Question:
If cp_atleast_3_months = Yes

Q179 I believe my pain is due to:

	**Yes** (1)	**No** (2)
The effects of treatment (for example, medication, surgery, radiation, prosthetic device) (1)	○	○
My primary disease (meaning the disease currently being treated and evaluated) (2)	○	○
A medical condition unrelated to my primary disease (for example, arthritis) (3)	○	○

Display This Question:
If cp_atleast_3_months = Yes

Q180 Please choose the adjective(s) below that apply to your pain. **Select all that apply.**

▢ Aching (1)

▢ Throbbing (2)

▢ Shooting (3)

▢ Stabbing (4)

▢ Gnawing (5)

▢ Sharp (6)

▢ Tender (7)

▢ Burning (8)

▢ Exhausting (9)

▢ Tiring (10)

▢ Penetrating (11)

© 2024 Smith KE et al. *JAMA Network Open.*

☐ Nagging (12)

☐ Numb (13)

☐ Miserable (14)

☐ Unbearable (15)

Display This Question:
If cp_atleast_3_months = Yes

Q181 Please indicate how, ***during the past week***, pain has interfered with the following: (**0 = Does not interfere** and **10 = Completely interferes**)

	Does Not Interfere	Completely Interferes
	0 1 2 3 4 5 6 7 8 9 10	
General activity ()		
Mood ()		
Walking ability ()		
Relations with other people ()		
Sleep ()		
Enjoyment of life ()		

Page Break

© 2024 Smith KE et al. *JAMA Network Open.*

Display This Question:
If cp_atleast_3_months = Yes

Q182 I prefer to take my pain medicine:

- ○ On a regular basis (1)
- ○ Only when necessary (2)
- ○ Do not take pain medicine (3)

Display This Question:
If cp_when_med = On a regular basis
Or cp_when_med = Only when necessary

Q183 I take my pain medicine ***(in a 24 hour period)***:

- ○ Not every day (1)
- ○ 1 to 2 times per day (2)
- ○ 3 to 4 times per day (3)
- ○ 5 to 6 times per day (4)
- ○ More than 6 times per day (5)

Display This Question:
If cp_when_med = On a regular basis
Or cp_when_med = Only when necessary

Q184 Do you feel you need a stronger type of pain medication?

- ○ Yes (1)
- ○ No (2)
- ○ Uncertain (3)

Display This Question:
If cp_when_med = On a regular basis
Or cp_when_med = Only when necessary

Q185 Do you feel you need to take more of the pain medication than your doctor has prescribed?

- ○ Yes (1)

© 2024 Smith KE et al. *JAMA Network Open.*

No (2)

Uncertain (3)

Display This Question:
If cp_when_med = On a regular basis
Or cp_when_med = Only when necessary

Q186 Are you concerned that you take too much pain medication?

Yes (1)

No (2)

Uncertain (3)

Display This Question:
If cp_when_med = On a regular basis
Or cp_when_med = Only when necessary

Q187 Are you having problems with side effects from your pain medication?

Yes (1)

No (2)

Uncertain (3)

Display This Question:
If cp_when_med = On a regular basis
Or cp_when_med = Only when necessary

Q188 Do you feel you need to receive further information about your pain medication?

Yes (1)

No (2)

Uncertain (3)

Display This Question:
If cp_atleast_3_months = Yes

Q189 Other methods I use to relieve my pain include: **(select all that apply)**

Kratom (8)

© 2024 Smith KE et al. *JAMA Network Open.*

- ☐ Warm compresses (1)
- ☐ Cold compresses (2)
- ☐ Relaxation techniques (3)
- ☐ Distraction (4)
- ☐ Biofeedback (5)
- ☐ Hypnosis (6)
- ☐ Other: (7) ____

Display This Question:
If cp_other_relief_methods = Kratom

Q349 Do you feel you need a stronger type of kratom for pain?

- ○ Yes (1)
- ○ No (2)
- ○ Uncertain (3)

Display This Question:
If cp_other_relief_methods = Kratom

Q350 Are you concerned that you take too much kratom for pain specifically?

- ○ Yes (1)
- ○ No (2)
- ○ Uncertain (3)

Display This Question:
If cp_other_relief_methods = Kratom

Q351 Are you having problems with side effects from your kratom that you take for pain specifically?

- ○ Yes (1)
- ○ No (2)

© 2024 Smith KE et al. *JAMA Network Open.*

◯ Uncertain (3)

Display This Question:
If cp_other_relief_methods = Kratom

Q352 Do you feel you need to receive further information about your kratom that you take for pain specifically?

◯ Yes (1)

◯ No (2)

◯ Uncertain (3)

End of Block: Chronic Pain

Start of Block: Chronic Fatigue Syndrome

X→

Q190 We would like to know more about if you have been feeling tired, weak, or lacking in energy in the ***past month***.

If you have been feeling tired for a long time, then compare yourself to how you felt ***when you were last well***.

	**Less than usual** (0)	**No more than usual** (1)	**More than usual** (2)	**Much more than usual** (4)
Do you have problems with tiredness? (1)	◯	◯	◯	◯
Do you need to rest more? (2)	◯	◯	◯	◯
Do you feel sleepy or drowsy? (3)	◯	◯	◯	◯
Do you have problems starting things? (4)	◯	◯	◯	◯
Do you lack energy? (5)	◯	◯	◯	◯
Do you have less strength in	◯	◯	◯	◯

© 2024 Smith KE et al. *JAMA Network Open.*

your muscles? (6)				
Do you feel weak? (7)	○	○	○	○
Do you have difficulties concentrating? (8)	○	○	○	○
Do you make slips of the tongue when speaking? (9)	○	○	○	○
Do you find it more difficult to find the right word? (10)	○	○	○	○

Q191 Click to write the question text

	**Better than usual** (0)	**No worse than usual** (1)	**Worse than usual** (2)	**Much worse than usual** (3)
How is your memory? (1)	○	○	○	○

End of Block: Chronic Fatigue Syndrome

Start of Block: Social Jetlag

Q192 I am a night-shift or rotating-shift worker.

○ Yes (1)

○ No (2)

Q353 What time do you typically ***wake up*** on days when you’re free to sleep in as late as you want ***or to wake up as early as you want***? These are days when you don’t have any particular obligations that you have to be awake for (e.g., work, school, appointments, caregiving duties).

© 2024 Smith KE et al. *JAMA Network Open.*

Q354 Hour?
▼ 1 (1) ... 12 (12)

Q355 Minutes?
▼ 00 (00) ... 59 (59)

Q356 AM or PM?

- AM (1)
- PM (2)

Q357 What time do you typically ***fall asleep*** on nights before those free days?

Q358 Hour?
▼ 1 (1) ... 12 (12)

Q359 Minutes?
▼ 00 (00) ... 59 (59)

Q360 AM or PM?

- AM (1)
- PM (2)

Q364 What time do you typically ***wake up*** on days ***when you do have particular obligations*** that you have to be awake for?

Q365 Hour?
▼ 1 (1) ... 12 (12)

Q366 Minutes?
▼ 00 (00) ... 59 (59)

Q367 AM or PM?

○ AM (1)

○ PM (2)

Q368 What time do you typically ***fall asleep*** on nights before those days with obligations?

Q369 Hour?
▼ 1 (1) ... 12 (12)

Q370 Minutes?
▼ 00 (00) ... 59 (59)

Q371 AM or PM?

○ AM (1)

© 2024 Smith KE et al. *JAMA Network Open.*

○ PM (2)

Page Break

© 2024 Smith KE et al. *JAMA Network Open.*

Q194 Do you have a different sleep-wake schedule on ***weekends*** (Saturday and Sunday), than on ***weekdays*** (Monday-Friday)?

○ Yes (1)

○ No (2)

Q195 Are your obligations different on ***weekends*** compared with ***weekdays***?

○ Yes, I have *more* obligations on weekends than weekdays. (1)

○ Yes, I have *fewer* obligations on weekends than weekdays. (2)

○ No, I have the *same* obligations on weekends and weekdays. (3)

Q196 Overall, do you consider yourself to be a morning person (an "early bird") or evening person (a "night owl")? Early birds prefer to wake up and be active earlier in the day. Night owls prefer to stay up and be active later in the day.

	**I'm Naturally an Early Bird** **I'm Naturally a Night Owl**
	0 10 20 30 40 50 60 70 80 90 100
1 ()	

Q197 Do you have a spouse or romantic partner?

○ Yes (1)

○ No (2)

Display This Question:
If sj_spouse = Yes

Q198 Overall, would you say your spouse or romantic partner is a morning person (an "early bird") or evening person (a "night owl"). Early birds prefer to wake up and be active earlier in the day. Night owls prefer to stay up and be active later in the day.

They're Naturally an "Early Bird" **They're Naturally a "Night Owl"**

© 2024 Smith KE et al. *JAMA Network Open.*

	0 10 20 30 40 50 60 70 80 90 100
1 ()	

Q199 Have you had this same preference ***your entire life***, or did you have a different sleep/wake pattern preference ***at an earlier time in your life***?

- ◯ I've been like this as long as I can remember (1)
- ◯ I wasn't always like this. (2)

Display This Question:
If sj_same_pref_for_life = I wasn't always like this.

Q200 Describe how old you were when your preferred sleep/wake pattern changed and why you believe it changed:

____

Page Break

© 2024 Smith KE et al. *JAMA Network Open.*

Q201 Please rate you strongly you agree with the following statements.

	**Strongly disagree** (0)	**Disagree** (1)	**Neither agree nor disagree** (2)	**Agree** (3)	**Strongly agree** (4)
I generally feel “in sync” with the sleep-wake schedules of most people. (1)	○	○	○	○	○
I feel like people judge me because of the time of day/night that I typically sleep. (2)	○	○	○	○	○
I feel like people judge me because of how much, or how little, I typically sleep. (3)	○	○	○	○	○
I get into conflicts or arguments with other people over my sleep-wake schedule. (4)	○	○	○	○	○
I am unable to participate in important activities because of my sleep-wake schedule. (5)	○	○	○	○	○

© 2024 Smith KE et al. *JAMA Network Open.*

I have a difficult time getting and/or maintaining a romantic relationship due to my typical wake-sleep schedule. (6)	○	○	○	○	○
I intentionally sleep at times when other people are awake to avoid having to do things I don't want to do. (7)	○	○	○	○	○
I intentionally sleep at times when other people are awake to avoid having to interact with them. (8)	○	○	○	○	○
I wish I could change when I sleep to make myself more "in sync" with other people. (9)	○	○	○	○	○

End of Block: Social Jetlag

Start of Block: Quality of Life

X→

Q202 This questionnaire asks how you feel about your quality of life, health, or other areas of your life. Please answer all the questions. If you are unsure about which response to give to a question, please choose the one that appears most appropriate. This can often be your first

© 2024 Smith KE et al. *JAMA Network Open.*

response.

Please keep in mind your standards, hopes, pleasures and concerns.

We ask that you think about your life in the ***last two weeks***.

	**Very poor** (1)	**Poor** (2)	**Neither poor nor good** (3)	**Good** (4)	**Very good** (5)
How would you rate your quality of life? (1)	○	○	○	○	○

Q203 Click to write the question text

	**Very dissatisfied** (1)	**Dissatisfied** (2)	**Neither satisfied nor dissatisfied** (3)	**Satisfied** (4)	**Very satisfied** (5)
How satisfied are you with your health? (1)	○	○	○	○	○

Q204 Click to write the question text

	**Not at all** (1)	**A little** (2)	**A moderate amount** (3)	**Very much** (4)	**An extreme amount** (5)
To what extent do you feel that physical pain prevents you from doing what you need to do? (1)	○	○	○	○	○

© 2024 Smith KE et al. *JAMA Network Open.*

How much do you need any medical treatment to function in your daily life? (2)	○	○	○	○	○
How much do you enjoy life? (3)	○	○	○	○	○
To what extent do you feel your life to be meaningful? (4)	○	○	○	○	○
How well are you able to concentrate? (5)	○	○	○	○	○
How safe do you feel in your daily life? (6)	○	○	○	○	○
How healthy is your physical environment? (7)	○	○	○	○	○

X→

Q375 Click to write the question text

	**Not at all** (1)	**A little** (2)	**Moderately** (3)	**Mostly** (4)	**Completely** (5)
Do you have enough energy for everyday life? (1)	○	○	○	○	○

© 2024 Smith KE et al. *JAMA Network Open.*

Are you able to accept your bodily appearance? (2)	○	○	○	○	○
Have you enough money to meet your needs? (3)	○	○	○	○	○
How available to you is the information that you need in your day-to-day life? (10)	○	○	○	○	○
To what extent do you have the opportunity for leisure activities? (4)	○	○	○	○	○

Q205 Click to write the question text

	**Very poor** (1)	**Poor** (2)	**Neither poor nor well** (3)	**Well** (4)	**Very Well** (5)
How well are you able to get around? (1)	○	○	○	○	○

Q206 Click to write the question text

© 2024 Smith KE et al. *JAMA Network Open.*

	**Very dissatisfied** (1)	**Dissatisfied** (2)	**Neither satisfied nor dissatisfied** (3)	**Satisfied** (4)	**Very satisfied** (5)
How satisfied are you with your sleep? (1)	○	○	○	○	○
How satisfied are you with your ability to perform your daily living activities? (2)	○	○	○	○	○
How satisfied are you with your capacity for work? (3)	○	○	○	○	○
How satisfied are you with yourself? (4)	○	○	○	○	○
How satisfied are you with your personal relationships? (5)	○	○	○	○	○
How satisfied are you with your sex life? (6)	○	○	○	○	○
How satisfied are you with the support you get from your friends? (7)	○	○	○	○	○
How satisfied are you with the conditions of your living place? (8)	○	○	○	○	○
How satisfied are you with	○	○	○	○	○

© 2024 Smith KE et al. *JAMA Network Open.*

your access to health services? (9)					
How satisfied are you with your transport? (10)	○	○	○	○	○

Q207 Click to write the question text

	**Never** (1)	**Seldom** (2)	**Quite Often** (3)	**Very Often** (4)	**Always** (5)
How often do you have negative feelings. such as blue mood, despair, anxiety, depression? (1)	○	○	○	○	○

End of Block: Quality of Life

(END OF CROSS-SECTIONAL SURVEY QUESTIONS)

© 2024 Smith KE et al. *JAMA Network Open.*

(BEGINING OF ECOLOGICAL MOMENTARY ASSESSMENT QUESTIONS)

Beginning of Day (BD) Prompt:

BD1) Did you take kratom within one hour before you went to bed (or lay down for sleep) last night?
Yes
No
Unsure

BD2) Did you take kratom within one hour before you actually *fell* asleep last night (the best that you can remember)?
Yes
No
Unsure

BD3) Approximately how many hours did you just sleep?
(Numerical dropdown in .25 increments from 0-24 hours)
0.00
0.25
0.5
0.75
1.00
1.25
1.50

© 2024 Smith KE et al. *JAMA Network Open.*

1.75
2.00
2.25
…etc.
9.75
10
11
12
…etc.
23
24

BD4) How would you rate the quality of your most recent sleep? (0-100 VAS)
Extremely poor=0 Extremely good=100

BD5) Whether or not you used kratom before bed—did the kratom you took today affect the *amount* of sleep that you just got? (This could be from the effects of kratom or from the effects of not using it.)
My sleep increased from using it
My sleep increased from *not* using it
My sleep decreased from using it
My sleep decreased from *not* using it
No, kratom didn't affect my sleep one way or another
Unsure

BD6) Whether or not you used kratom before bed—did the kratom you took today affect the *quality* of sleep that you just got? (This could be from the effects of kratom or from the effects of not using it.)
My sleep quality increased from using it
My sleep quality increased from *not* using it
My sleep quality decreased from using it
My sleep quality decreased from *not* using it
No, kratom didn't affect my sleep quality one way or another
Unsure

BD7) You can use this space to tell us anything else—for example, to clarify how using versus not using kratom may have affected your sleep, or to explain any "not sure" answers.
(Open text box)

BD Message: Thank you for your responses! Please remember to turn off your "Do Not Disturb" settings today to ensure that you receive notifications for study prompts. We hope you have a great rest of your day.

© 2024 Smith KE et al. *JAMA Network Open.*

<u>Event Contingent (EC) Entries:</u>

EC1) What kind of kratom or kratom product did you take? (Select all that apply)
___Prepared tea
___Kratom juice or smoothie
___Prepackaged extract beverage
___Prepackaged extract shot
___Prepared/pre-packaged capsules
___Kratom powder in parachute
___Loose kratom powder/pulverized plant matter (not in capsules or other vehicle for swallowing)
___Dried leaf
___Raw, fresh leaf
___Gummies/edibles
___Kratom vape
___Other

EC1.1) Please describe the other kratom or kratom product that you took:
(Open text box)

EC2) Are you finished with this use for the time being?
Yes
No, I'm still sipping my kratom beverage, or eating my kratom food, or taking my powder etc.
No, I took a portion of it and will save the rest for later.

EC3) How did you take the kratom? (Select the appropriate unit(s) of measurement. If you took more than one product, please respond based on the product you used more of.)
Because it is possible someone mixed and matched, I don't want this to be open to multiple responses, even though it's unlikely to be more than one.
___spoonful(s)
___tablespoon(s)
___grams
___cup(s)
___fl. ounces.
___milliliters
___capsules medium (standard)
___capsules large/jumbo
___pieces of edible product
___leaves
___I don't know
___I'm still in the process of consuming at my own pace

If the answer is "I don't know" or "I'm still in the process of consuming at my own pace", then EC4 will not display

© 2024 Smith KE et al. *JAMA Network Open.*

EC4) Approximately how many (spoonfuls, tablespoons, grams, etc.) of these did you take? (Please indicate the quantity you took based on your answer to the previous question. If you took more than one product, please respond based on the product you used more of.**)**
(Numerical dropdown in .25 increments from 0-10; .5 increments from 11-20; 1.0 increments from 21-100; last option listed is >100)
0
0.25
0.5
0.75
1.00
1.25
…
10
10.5
11
11.5
12
12.5
…
20
21
22
23
…
98
99
100
more than 100

EC5) Please select all of the reasons below that motivated your kratom use just now.
(Right now, there are 20, so we need to program this to run from 1-20. This is forced choice, meaning they must select and rank at least one item)
___Relieve pain
___Help me sleep
___ Feel less depressed or sad
___ Stop worrying
___ Calm me down
___ Relieve kratom withdrawal
___ Relieve other drug withdrawal
___ Stop kratom craving
___ Stop other drug craving
___ Escape boredom
___Increase energy
___ Increase focus/alertness
___ Increase productivity
___ Improve mood

© 2024 Smith KE et al. *JAMA Network Open.*

___ Relax and unwind
___ Feel good
___ Feel high
___ Enhance the effects of another drug
___ Other

EC6) If there are other reasons that motivated your kratom use, please describe:
(Open text box)

EC7) What were you doing, or preparing to do, when you used kratom just now? (Select all that apply)
___Sleeping
___Waking up
___Trying to rest/sleep
___Relaxing
___Socializing in person
___Socializing online (social media, video)
___Texting
___Having an unpleasant social interaction (arguing, etc.)
___Physical activity for health or pleasure (working out, walking, sports)
___Recreation requiring active participation (games, hobbies, creative activities)
___Recreation that I can enjoy more passively (reading, podcasts, internet, TV, music)
___Riding/travelling
___Thinking/planning
___Working
___School work
___Eating
___ Foreplay/Sex
___Household chores or personal hygiene
___Shopping/Errands
___Childcare/Elder care
___ Other

EC7.1) Please describe what you were doing, or preparing to do, when you used kratom just now:
(Open text box)

EC8) Where were you when you used? (Select one)
___Home
___Work
___School
___Vehicle (car, bus, train)
___Waiting for ride, bus, etc.
___Another's home
___Outdoors
___Restaurant/store

© 2024 Smith KE et al. *JAMA Network Open.*

___Bar/club
___Clinic / doctor
Other:__________

If "Other", then pipe to open text response on separate page (EC7_Other) with the following message:

EC8.1) Please describe where you were when you used kratom:
(Open text box)

EC9) What vendor did this kratom product come from?
(Open text box)

EC10) Thank you for your responses! Now you have the opportunity to upload a picture of your kratom product.
(Image submission)

© 2024 Smith KE et al. *JAMA Network Open.*

Event Contingent Follow-up (FU):

FU1) Have you used more kratom since you last reported using it?
___Yes
___No
___I'm not done using (e.g., I'm still sipping my kratom beverage).

FU2) Did you use more kratom than you initially meant to?
___Yes
___No

FU3) How much do you feel the effects of kratom right now?
(0-100 VAS)

Not at all=0 I feel them at their peak=100

FU4) What effects are you *currently* experiencing that you attribute to kratom? (Select all that apply)
___Itchiness
___Sleepiness/Lethargy
___Increased movement
___Restlessness
___Energized
___Sociable/Talkative
___Paranoia
___Euphoria
___Warmness/flushed
___Disoriented
___Focused/Alert
___Increased, faster heartbeat
___Decreased, slower heartbeat
___Slower, shallow breathing
___Eye tearing
___Eye puffiness
___Dilated pupils
___Constricted pupils
___Constipated
___Nauseated
___Abdominal discomfort/diarrhea
___Increased appetite
___Loss of appetite
___Decreased libido
___Increased libido

FU5) How anxious have you been since your last kratom use?
(0-100 VAS) Fully relaxed, no anxiety=0 Extremely anxious=100

© 2024 Smith KE et al. *JAMA Network Open.*

FU6) Describe how your mood is since your last kratom use.
(0-100 VAS) Very poor mood=0 Enhanced mood and sense of well-being=100

FU7) How sad have you felt since your last kratom use?
(0-100 VAS) Not at all sad=0 Extremely sad=100

FU8) Have you been in pain since your last kratom use?
(0-100 VAS) Completely pain free=0 Severe pain=100

FU9) Did you crave kratom since your last kratom use?
(0-100 VAS) No kratom craving=0 Intense kratom craving=100

FU10) Did you crave another drug since your last kratom use?
(0-100 VAS) No craving for another drug=0 Intense craving for another drug=100

FU11) Which other substances have you taken during or since your last use of kratom?
(Select all that apply)
___Nicotine products
___Liquor
___Beer
___Wine
___Coffee
___Caffeine (preworkout, energy drinks, caffeine pills)
___Tea
___Kava
___Grapefruit juice
___Cannabis
___CBD (cannabidiol)
___Prescription opioids
___Suboxone/Subutex prescribed to you
___Suboxone/Subutex *not* prescribed to you
___Methadone prescribed to you
___Heroin
___Fentanyl
___Ecstasy/MDMA
___Street meth
___Cocaine/crack cocaine
___Amphetamine pills (Adderall, Modafinil)
___Nootropics (Phenibut, Tianeptine)
___Anti-anxiety drugs (Xanax, Valium)
___Psychedelics
___Antidepressant medication (Zoloft, Prozac, Lexapro)
___Antipsychotic medication (Abilify, Haldol)
___Vitamins
___Over-the-counter pain relievers (Advil, Tylenol)
___I have not used any other substances during or since my last kratom use
___Other substance not listed above

© 2024 Smith KE et al. *JAMA Network Open.*

FU11.1) What other substance(s) have you taken during or since your last kratom use?
(Open text box)

FU12) How has the most recent kratom use affected your productivity? (If you had no intention of being productive, you can put the slider at the midpoint.) (0-100 VAS)
Extremely bad for my productivity=0 Extremely good for my productivity=100

FU13) How impaired do you feel as a result of the kratom? (0-100 VAS)
Not at all impaired=0 Unable to function=100

FU14) Based on how you feel from your last kratom use, how confident would you be driving a vehicle right now? (0-100 VAS)
Extremely unconfident=0 Extremely confident=100

FU15) Is the kratom working for you the way you wanted it to? (0-100 VAS)
Not at all=0 Completely=100

FU16) Are you going to bed soon (or already in bed, trying to sleep)?
Yes
No

<u>Random Prompts (RP):</u>

RP1) Have you used any kratom since the last time you reported use?
Yes
No
I hadn't stopped—I'm still in the middle of taking/sipping my kratom

RP2) How do you feel right now? (Select all that apply)
___Relaxed
___Contented
___Vigorous
___Confident
___Lively
___Happy
___Carefree
___Afraid
___Anxious
___On edge
___Uneasy
___Worn out
___Fatigued
___Exhausted
___Discouraged
___Hopeless

© 2024 Smith KE et al. *JAMA Network Open.*

___Angry
___Annoyed
___Resentful
___Bored
___Lonely

RP3) Do you feel anxious feel right now?
(0-100 VAS) Fully relaxed, no anxiety=0 Extremely anxious=100

RP4) How is your mood right now?
(0-100 VAS) Very poor mood=0 Enhanced mood and sense of well-being=100

RP5) Do you feel sad right now?
(0-100 VAS) Not at all sad=0 Extremely sad=100

RP6) Are you in pain right now?
(0-100 VAS) Completely pain free=0 Severe pain=100

RP7) Do you crave kratom right now?
(0-100 VAS) No kratom craving=0 Intense kratom craving=100

RP8) Do you crave another drug right now?
(0-100 VAS) No craving for another drug=0 Intense craving for another drug=100

© 2024 Smith KE et al. *JAMA Network Open.*

End of Day Prompt:

ED1) Were there any times that you used kratom during the past 24 hours and did not report it?
Yes
No

[*“What time” items are only for people who said yes to ED1, about unreported use.]*
ED1.1) It's very important that you report kratom use soon after it occurs. But if there were times you used kratom today but were unable to report it right after use, please let us know about it here: **During what times in the past 24 hours did you use kratom and not report it on the app?** (Select all that apply)
____6:00 a.m. to 11:00 a.m.
____11:00 a.m. to 4:00 p.m.
____4:00 p.m. to 10:00 p.m.
____10:00 p.m. to 12:00 a.m.
____12:00 a.m. to 6:00 a.m.
____I recorded all of my kratom use events for the day

Selected responses to prompt relevant/corresponding ED1.2 through ED1.6 if possible.

ED1.2) How many times did you use kratom and not report it between 6:00 a.m. and 11:00 a.m.? (Numerical dropdown up to 10)
1
2
3
…
10

ED1.3) How many times did you use kratom and not report it between 11:00 a.m. and 4:00 p.m.? (Numerical dropdown up to 10)
1
2
3
…
10

ED1.4) How many times did you use kratom and not report it between 4:00 p.m. and 10:00 p.m.? (Numerical dropdown up to 10)
1
2
3
…
10

ED1.5) How many times did you use kratom and not report it between 10:00 p.m. and 12:00 a.m.? (Numerical dropdown up to 10)
1

© 2024 Smith KE et al. *JAMA Network Open.*

2
3
…
10

ED1.6) How many times did you use kratom and not report it between 12:00 a.m. and 6:00 a.m.? (Numerical dropdown up to 10)
1
2
3
…
10

ED2) Did you use any of the below substances that you forgot or didn't report earlier?
Select only those that you used today but *didn't* report earlier.
___Nicotine products
___Liquor
___Beer
___Wine
___Coffee
___Caffeine (preworkout, energy drinks, caffeine pills)
___Tea
___Kava
___Grapefruit juice
___Cannabis
___CBD (cannabidiol)
___Prescription opioids
___Suboxone/Subutex *not* prescribed to you
___Suboxone/Subutex prescribed to you
___Methadone prescribed to you
___Heroin
___Fentanyl
___Ecstasy/MDMA
___Street meth
___Cocaine/crack cocaine
___Amphetamine pills (Adderall, Modafinil)
___Nootropics (Phenibut, Tianeptine)
___Anti-anxiety drugs (Xanax, Valium)
___Psychedelics
___Antidepressant medication
___Antipsychotic medication
___Vitamins
___Over-the-counter pain relivers (Advil, Tylenol)
___I have not used any of these
___Other

ED2.1) What other substance(s) did you take today, but forgot to report?

(Open text box)

ED3) Did you use kratom at all today?
Yes
No

ED4) [*Only for people who responded "no" to ED3.]*
Was there a specific reason that you did not use kratom today? Please describe...
(Open text box)

ED5) Overall, did your kratom use today have the results and effects you wanted?
Yes
No
Unsure

ED6) Overall, was kratom a help or hindrance in your being able to live out your day and do what you wanted and/or needed to do?
Help
Hindrance
A bit of both
Unsure

ED7) Overall, was kratom a help or hindrance to your *productivity* today?
Help
Hindrance
A bit of both
Unsure
Not applicable: I wasn't intending to be productive today

ED8) What was the #1 reason that motivated your kratom use today?
___Relieve pain
___Help me sleep
___ Feel less depressed or sad
___ Stop worrying
___ Calm me down
___ Relieve kratom withdrawal
___ Relieve other drug withdrawal
___ Stop kratom craving
___ Stop other drug craving
___ Escape boredom
___Increase energy
___ Increase focus/alertness
___ Increase productivity
___ Improve mood
___ Relax and unwind
___ Feel good
___ Feel high

© 2024 Smith KE et al. *JAMA Network Open.*

___ Enhance the effects of another drug
___ Other

ED8.1) Please describe the #1 reason that motivated your kratom use today:
(Open text box)

ED9) Overall, how would you characterize the kratom effects that you experienced today?
__Effects were compatible with my daily obligations and helped me achieve them
__Effects were compatible with my daily obligations, though not especially helpful for them
__Effects were not compatible with my daily obligations
__Effects were not compatible with my daily obligations, and they sometimes undermined my ability to meet my daily obligations
__I didn't take kratom enough today to know
__None of those are quite true for me

ED10) Do you plan to use any kratom tomorrow?
Yes
No
Not sure

ED11) Is there anything else you would like to tell us?
(Open text box)

(END OF ECOLOGICAL MOMENTARY ASSESSMENT QUESTIONS)

© 2024 Smith KE et al. *JAMA Network Open.*

eAppendix 2. Clustering Procedure

We used cluster analysis to classify the patterns of kratom use observed during the study. This clustering was based on self-reported use events from the ecological momentary assessment (EMA) data, specifically from the four types of EMA in which the time of use could be reported. These were:

1) event-contingent reports, initiated by the participant after a use event,
2) follow-up reports, prompted within 15-120 minutes after an event-contingent report 3) randomly prompted reports 4) end-of-day reports.

In all of these except event-contingent reports, the participant could report the times of any use events that were not already reported in the day. The figure below shows the times of all reported use events, aligned by each participant's self-scheduled beginning of day (BOD).

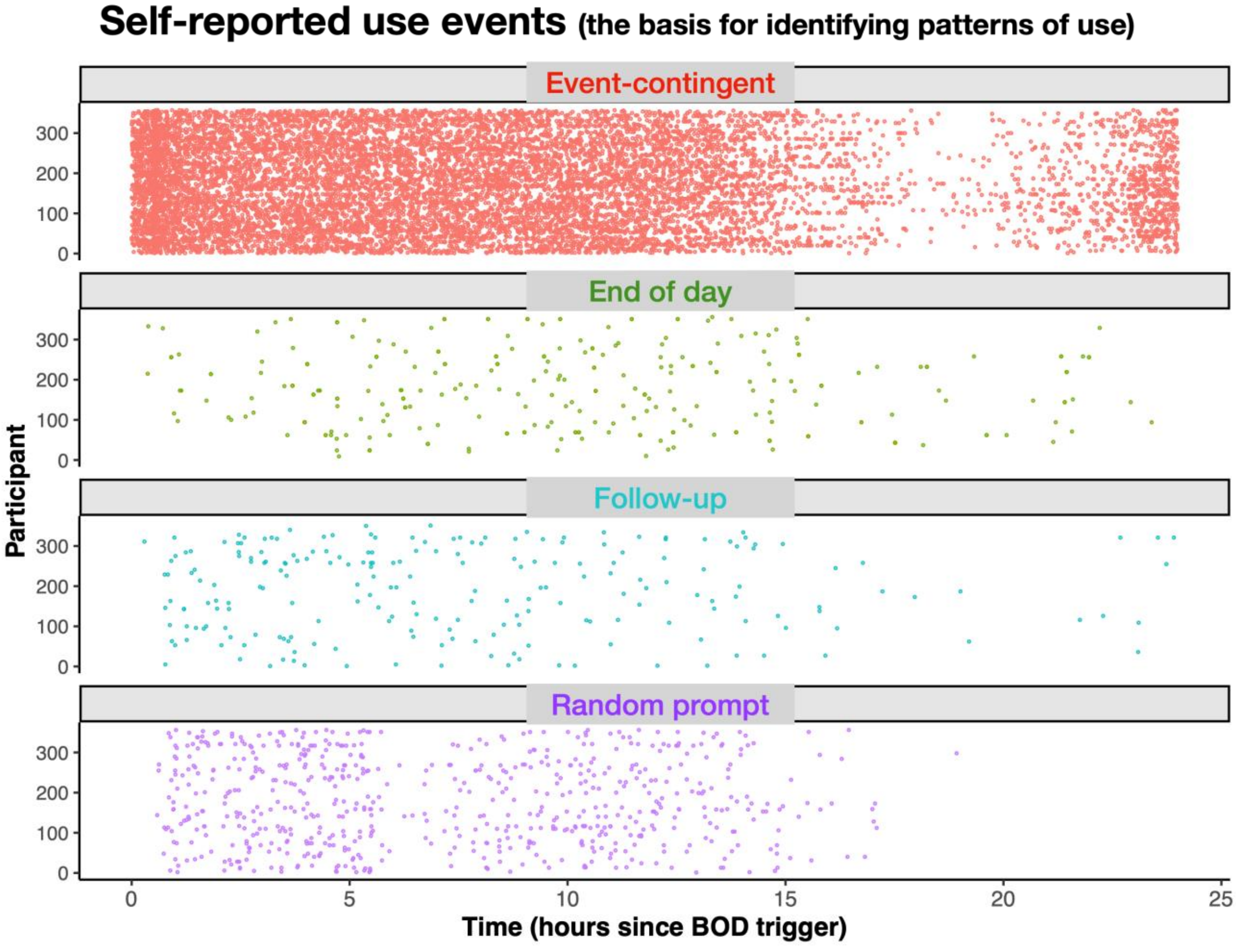

These data were compiled into 24 1-hour bins for each particpant (i.e., total number events in each hour of day collapsed across the participant's whole time in the study, relative to the time scheduled for the beginning of each day). Then, to explicitly consider both the frequency of use and the within-day temporal pattern of use, each participant's data were expressed in two independent forms:

1) centered and standardized *between* participants (personal *level* of use)
2) centered and standardized *within* participants (personal *pattern* of use)

© 2024 Smith KE et al. *JAMA Network Open.*

Clustering was conducted with the Flexible Mixture Modeling package (flexmix) (Leisch, 2004) based simultaneously on both forms of the the event-time data. The Bayesian Information Criterion indicated that the best fit was obtained with five clusters. We labeled these cluster A through E, with A having the frequent use and E having the lowest frequency of use. The plot below shows the means and 95% confidence intervals for the raw data (top row) and for the normalized data used for clustering (bottom row).

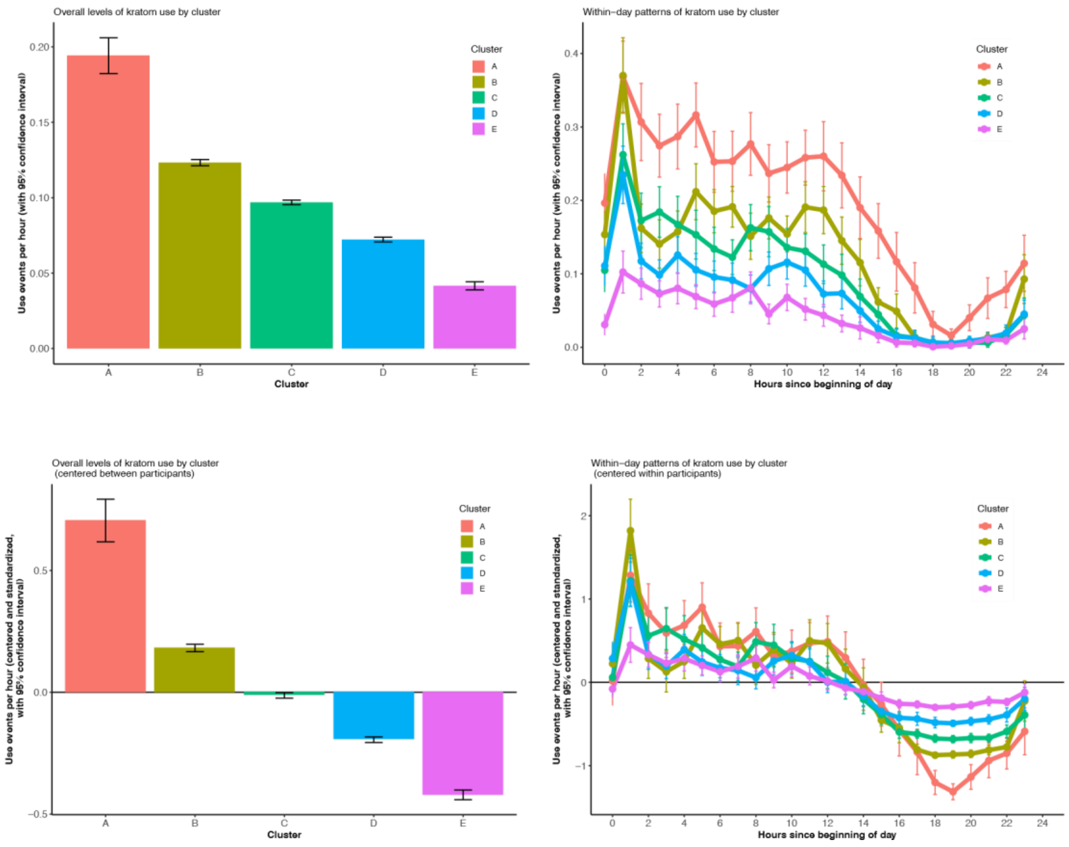

Comparing the overall levels of use in the clusters (bottom left panel), there was a clear step-wise decrease in the amount of use from clusters A through E. Comparing the within day patterns (bottom right panel), all clusters had their highest level of use early in the day, but this within-day patterns was more pronounced in the higher-use clusters.

eReference

Leisch, F. (2004). FlexMix: A General Framework for Finite Mixture Models and Latent Class Regression in R. *Journal of Statistical Software*, *11*(8), 1 - 18. https://doi.org/10.18637/jss.v011.i08

© 2024 Smith KE et al. *JAMA Network Open.*

eTable 1. Kratom Dosage Forms and Units

Form	Units	Mean Amount	Std. Dev.	Minimum	Maximum	N
LoosePowder	Teaspoons	1.8	1.1	0.2	6	97
LoosePowder	Grams	4.2	2.6	0.5	13	95
PrepCaps	CapsMed	6.5	5.2	0.5	26	73
LoosePowder	Tablespoons	1.9	1.1	0.5	4.8	48
LoosePowder	Spoonfuls	1.8	1	0.2	4	46
PrepCaps	Grams	3.8	1.7	1	8	26
PrepTea	Grams	4.4	3.2	1.5	12.8	26
PrepCaps	CapsJumbo	8.2	7	0.5	28.1	24
JuiceSmoothie	Grams	4	2.5	1.6	10.8	16
ExtractLiquid	Milliliters	7.7	4.3	2	15	14
Parachute	Grams	4.7	2.4	1.1	10	14
PrepTea	Teaspoons	1.6	0.9	0.3	3	13
JuiceSmoothie	Teaspoons	1.3	0.7	0.4	2	11
PrepTea	Spoonfuls	2.5	1.4	0.4	5	11
ExtractCapsules	CapsMed	2.6	2.6	1	8	9
ExtractCapsules	CapsJumbo	1.6	1	0.2	3	7
ExtractLiquid	FluidOz	5.1	6.3	0.5	15	7
PrepTea	Tablespoons	1.7	0.6	1	2.5	7
Parachute	Teaspoons	1.5	1	0.4	3	6
PrepCaps	Teaspoons	1.8	1.4	0.5	4.5	6
ExtractCapsules	Grams	1.6	0.8	0.6	2.6	5
Parachute	Spoonfuls	2.2	1.7	1	5	5
ExtractCapsules	Pieces	5.5	6.5	1	15	4
JuiceSmoothie	Cups	2.6	1.1	1.5	4	4
PrepTea	FluidOz	5.1	4	2	9.7	3
JuiceSmoothie	Spoonfuls	1.8	0.4	1.5	2	2
Parachute	Tablespoons	1.1	0.6	0.6	1.5	2
JuiceSmoothie	FluidOz	2.9	—	2.9	2.9	1

© 2024 Smith KE et al. *JAMA Network Open.*

eFigure 1. Proportion of Days With Kratom Use

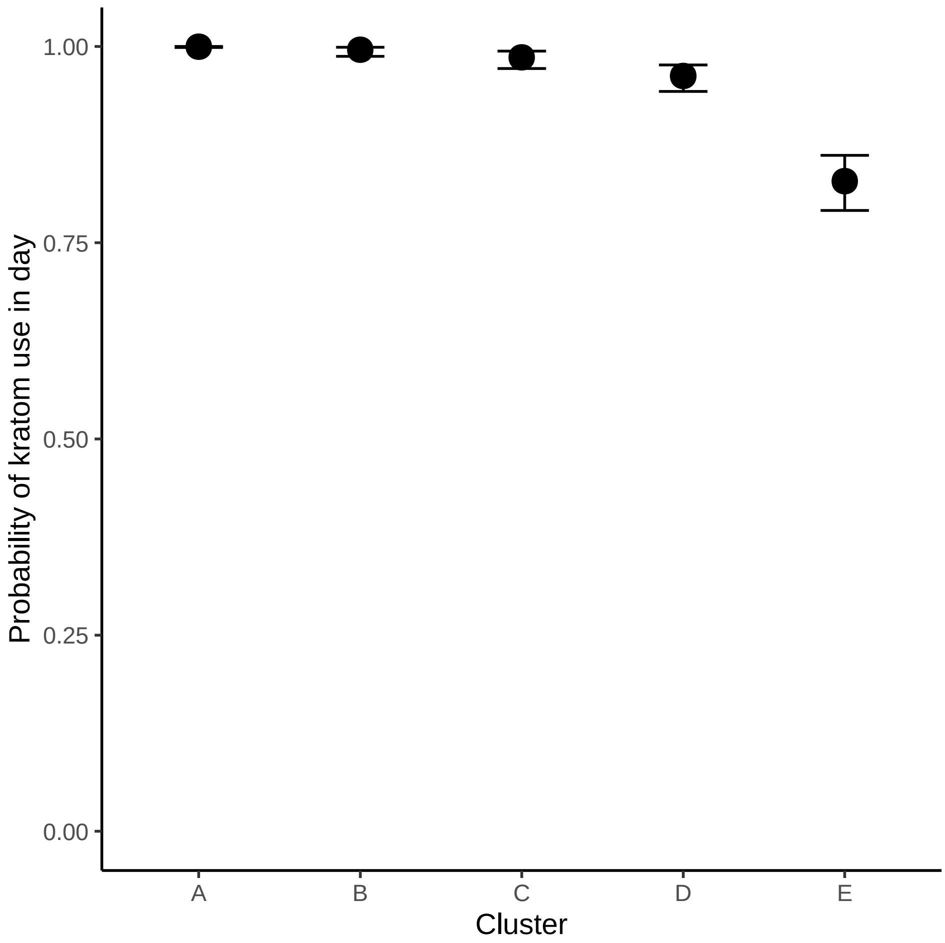

eFigure 1. Proportion of days with any kratom use, by cluster. Black points represent the mean proportion of days with use within the cluster, and error bars represent Bayesian 90% credible intervals. Blue points represent values for individual particpants.

© 2024 Smith KE et al. *JAMA Network Open.*

eFigure 2. Locations and Activities When Using Kratom

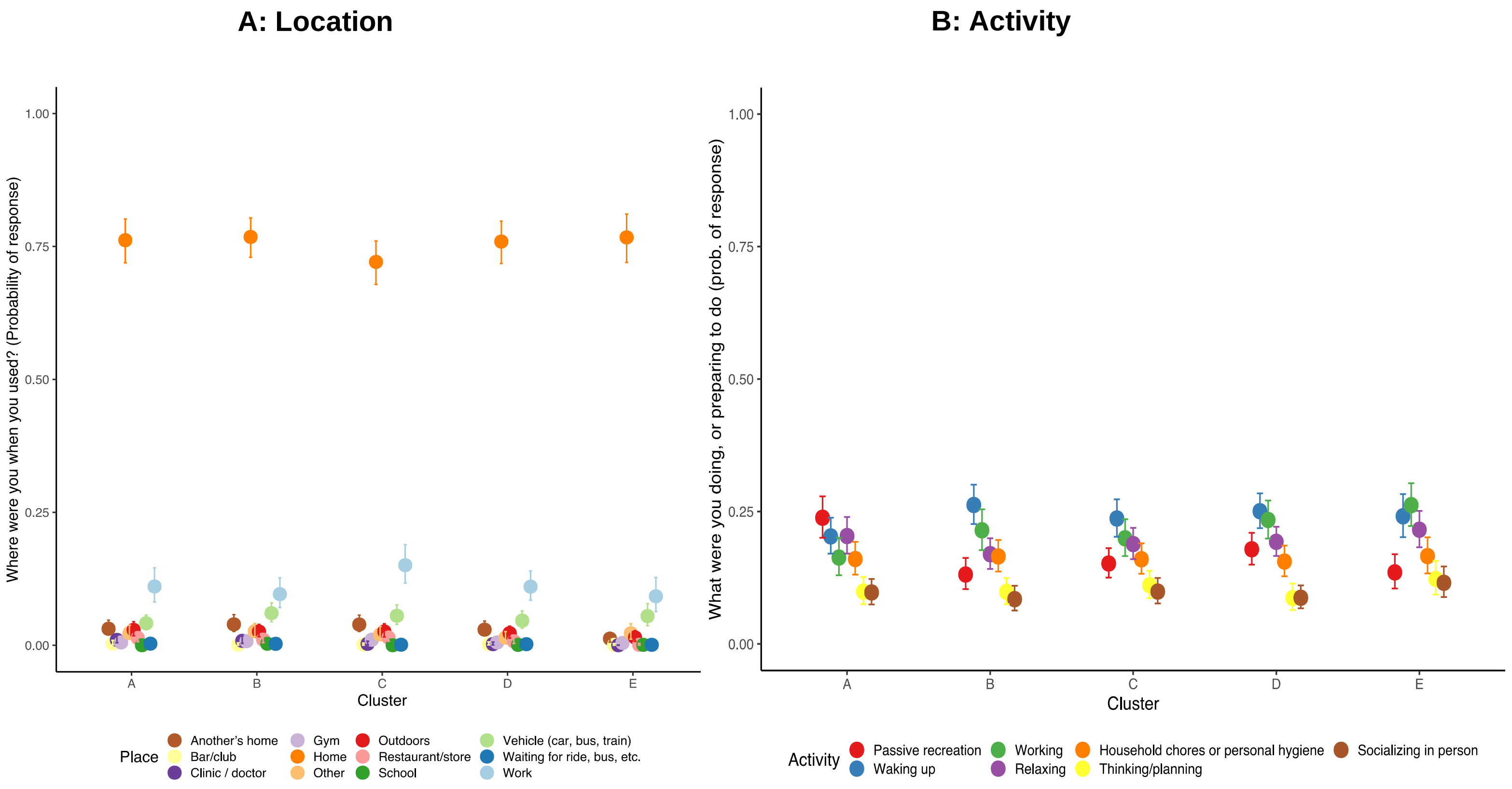

eFigure 2. Responses to the questions, "Where were you when you used?" (*panel A*) and "What were you doing, or preparing to do, when you used kratom just now?" (*panel B*) from ecological momentary assessment (EMA). Both questions were asked each time a participant made an event-contingent report of kratom use. Points represent mean probability of response, and error bars represent 90% credible intervals. Activities that were not endorsed in at least 10% of reports for at least one cluster were excluded from analysis. "Passive recreation" refers to "Recreation that I can enjoy more passively (reading, podcasts, internet, TV, music)."

© 2024 Smith KE et al. *JAMA Network Open.*

eTable 2. Demographic Characteristics by Kratom-Use Cluster (Clusters A-E)

**Kratom use cluster**[1]	**A (n = 56)**	**B (n = 65)**	**C (n = 71)**	**D (n = 83)**	**E (n = 80)**
**Age**					
Mean (SD)	42.4 (12.1)	37.3 (9.6)	39.8 (11.3)	36.6 (10.6)	35.2 (10.9)
**Sex/gender [n (%)]**					
Male	29 (51.8)	30 (46.2)	42 (59.2)	47 (56.6)	50 (62.5)
Female	26 (46.4)	33 (50.8)	27 (38.0)	33 (39.8)	28 (35.0)
Nonbinary	1 (1.8)	2 (3.1)	2 (2.8)	3 (3.6)	2 (2.5)
**Race/ethnicity**[2] **[n (%)]**					
White/European	54 (96.4)	59 (90.8)	61 (85.9)	77 (92.8)	73 (91.3)
Hispanic/Latino	3 (5.4)	4 (6.2)	5 (7.0)	3 (3.6)	7 (8.8)
Native American/Pacific Islander	2 (3.6)	4 (6.2)	1 (1.4)	4 (4.8)	2 (2.5)
Black/African American	1 (1.8)	3 (4.6)	1 (1.4)	2 (2.4)	3 (3.8)
Biracial/Multiracial	1 (1.8)	7 (10.8)	3 (4.2)	3 (3.6)	3 (3.8)
Asian	1 (1.8)	3 (4.6)	4 (5.6)	0	2 (2.5)
Middle Eastern	0	2 (3.1)	0	3 (3.6)	0
Indian	0	0	0	0	1 (1.3)
**Sexual orientation [n (%)]**					
Heterosexual	48 (85.7)	50 (76.9)	56 (78.9)	69 (83.1)	57 (71.3)
Gay/Lesbian	1 (1.8)	2 (3.1)	3 (4.2)	1 (1.2)	4 (5.0)
Bisexual	3 (5.4)	9 (13.8)	7 (9.9)	7 (8.4)	9 (11.3)
Asexual	1 (1.8)	0	0	1 (1.2)	3 (3.8)
Queer	2 (3.6)	2 (3.1)	1 (1.4)	1 (1.2)	2 (2.5)
Don’t know	0	0	1 (1.4)	1 (1.2)	0
Prefer not to say	0	1 (1.5)	2 (2.8)	1 (1.2)	4 (5.0)
Other	1 (1.8)	1 (1.5)	1 (1.4)	2 (2.4)	1 (1.3)

[1]Excluding 2 completers who were not assigned a cluster. [2]Choices are not mutually exclusive.

© 2024 Smith KE et al. *JAMA Network Open.*

eFigure 3. Broad Motivations for Use

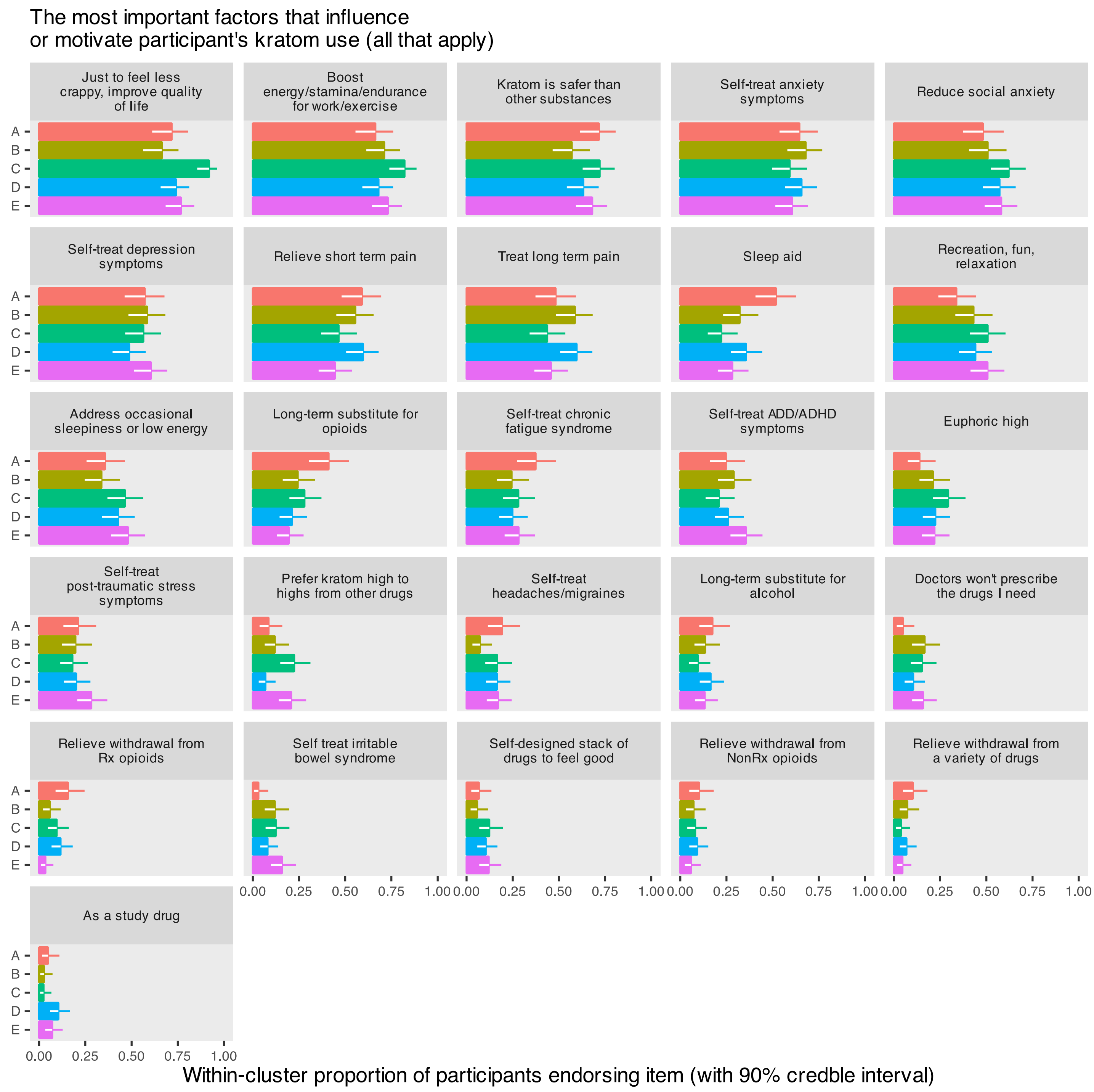

eFigure 3. Broad motivations for kratom use in each cluster (mean and 90% CI), reported (in survey) as being an important factor that influences or motivates the participant's kratom use. Data were obtained by providing the participant with a list from which all items that applied could be endorsed. Items that were not endorsed by at least 10% of at least one cluster were excluded from analysis.

© 2024 Smith KE et al. *JAMA Network Open.*

eFigure 4. Proximal Motivations for Kratom Use

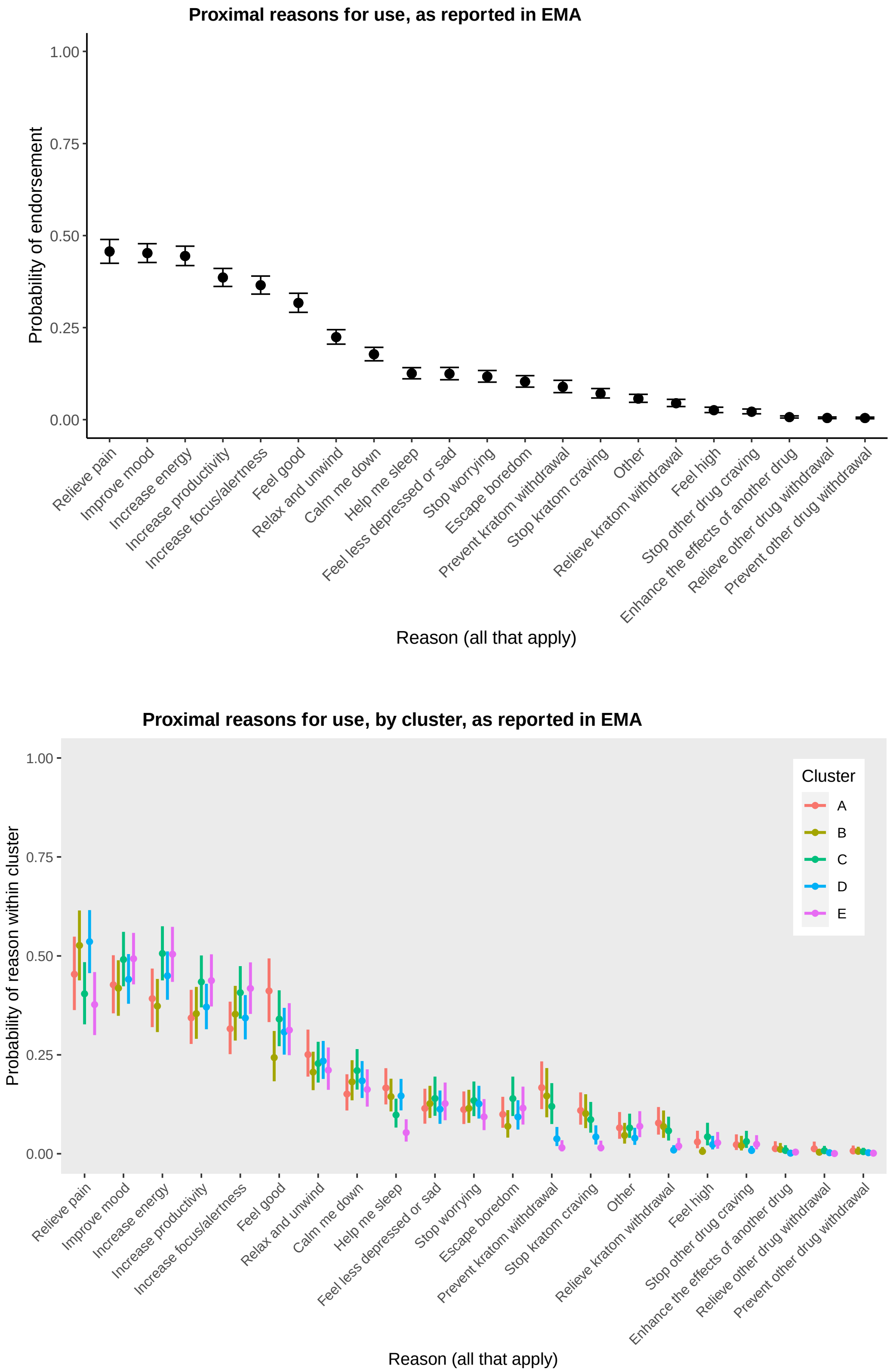

eFigure 4. Responses (mean and 90% CI) to the item, " Please select all of the reasons below that motivated your kratom use just now." *Upper panel*: Large black points represent the overall probability of the response, and error bars represent 90% credible intervals. Jittered blue points represent probability of each individual participant endorsing the item. *Lower panel*: Cluster means and credible intervals.

© 2024 Smith KE et al. *JAMA Network Open.*

eFigure 5. Anxiety, Pain, Mood, and Sadness

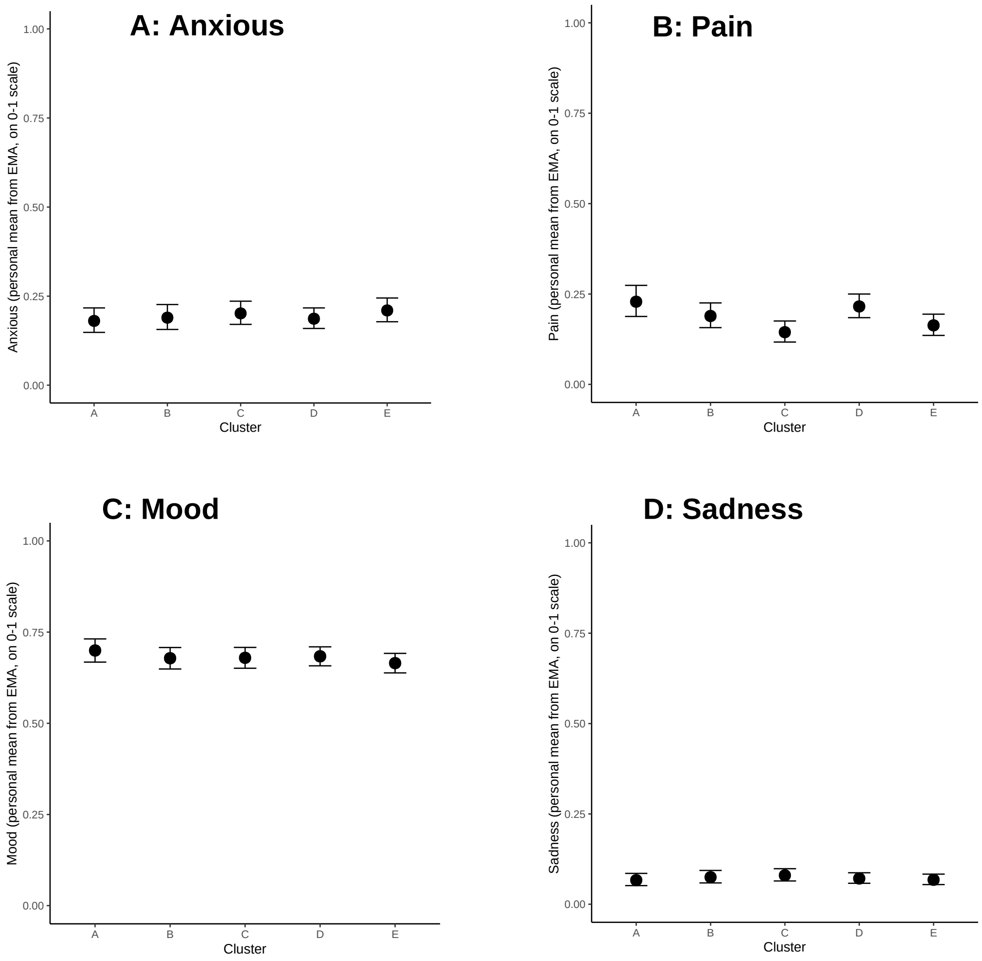

eFigure 5. Responses to the items, "How anxious have you been since your last kratom use?" (*panel A*), "Have you been in pain since your last kratom use?" (*panel B*), "Describe how your mood is since your last kratom use" (*panel C*), and " How sad have you felt since your last kratom use?" (panel D), from EMA. All items were presented in each follow-up report, some time *after* an event-contingent report of kratom use. Black points represent the mean, and error bars represent 90% credible intervals. Each blue point represents the mean for an individual participant. All items were answered on a visual analog scale of 0-100 and converted to 0-1 for analysis and plotting.

eFigure 6. Craving and More Kratom Use Than Intended

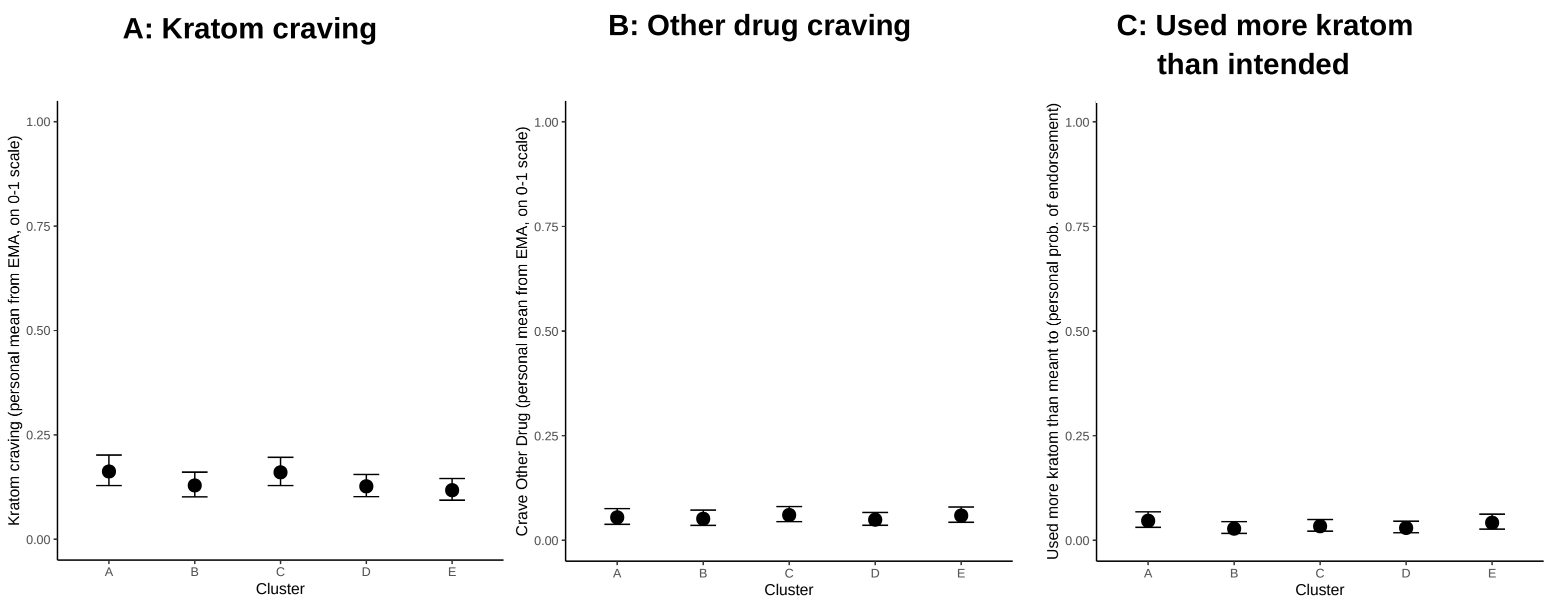

eFigure 6. Responses to the questions, " Do you crave kratom right now?" (*panel A*), " Do you crave another drug right now?" (*panel B*), and " Did you use more kratom than you initially meant to?" (*panel C*) from EMA. The questions in panels A and B were asked each time a participant made an event-contingent report of kratom use. The question in panel C was asked in each follow-up report, some time *after* an event-contingent report of kratom use. Black points represent the mean or probability, and error bars represent Bayesian 90% credible intervals. Each blue point represents the mean or probability for an individual participant. The craving questions were answered on a scale of 0-100 (visual analog scale) and converted to 0-1 for analysis and plotting. The scale for panel C represents the probability of saying "Yes."

© 2024 Smith KE et al. *JAMA Network Open.*

eFigure 7. Lifetime Medication for Opioid Use Disorder (Buprenorphine or Methadone)

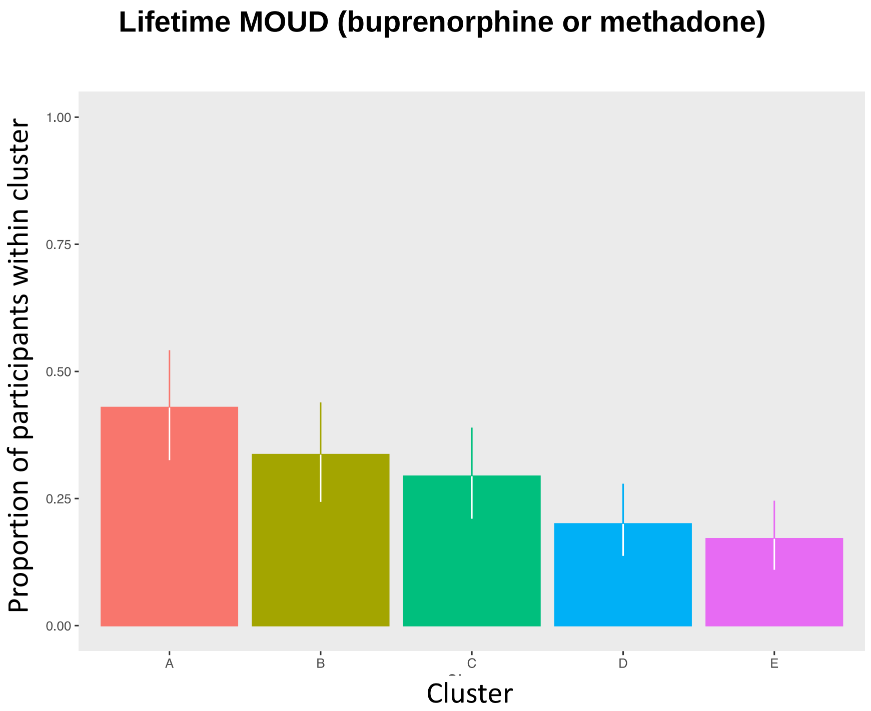

eFigure 7. Proportion (and 90% CI) of each cluster who had received medication for opioid use disorder (MOUD) in their lifetime, as reported in survey.

© 2024 Smith KE et al. *JAMA Network Open.*

eFigure 8. Kratom Use After Waking

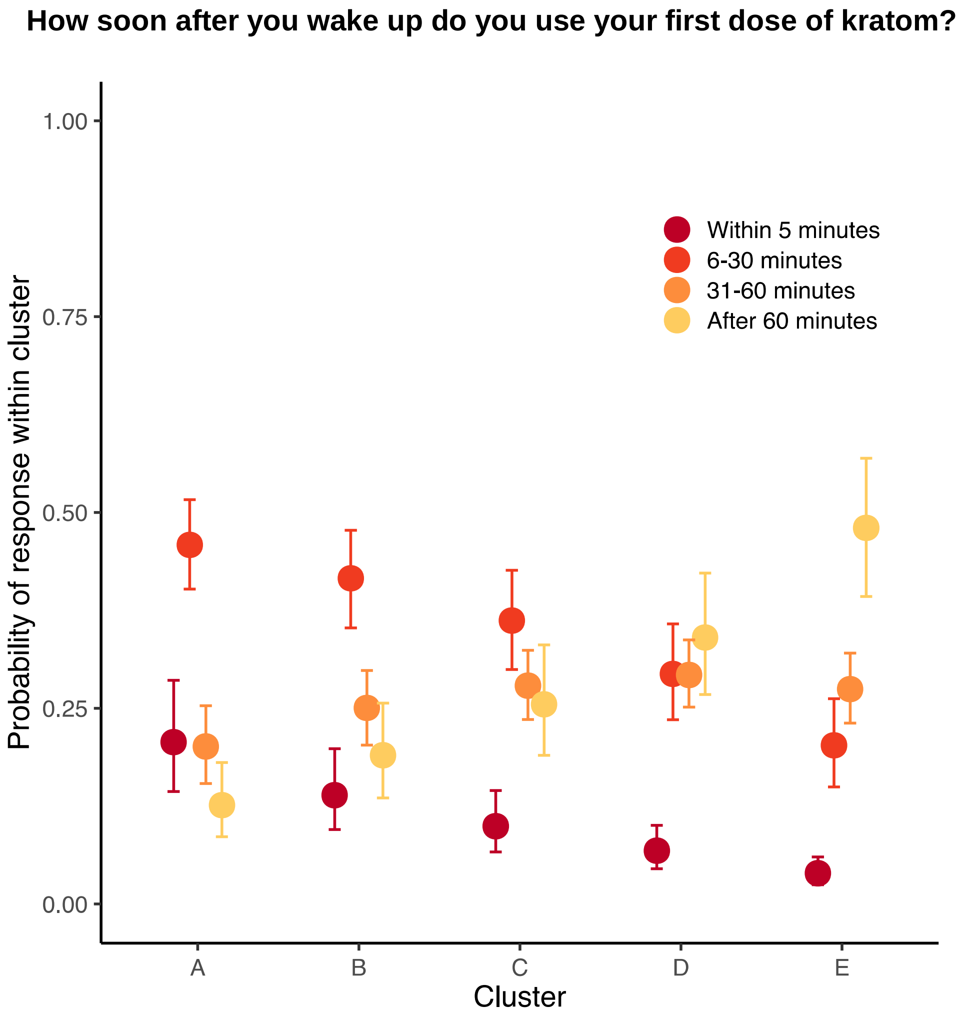

eFigure 8. Responses to the item, "How soon after you wake up do you use your first dose of kratom?", by each cluster. Points represent the mean probability of the response within the cluster, and error bars represent 90% credible intervals.

© 2024 Smith KE et al. *JAMA Network Open.*

eFigure 9. Kratom Effects on Sleep

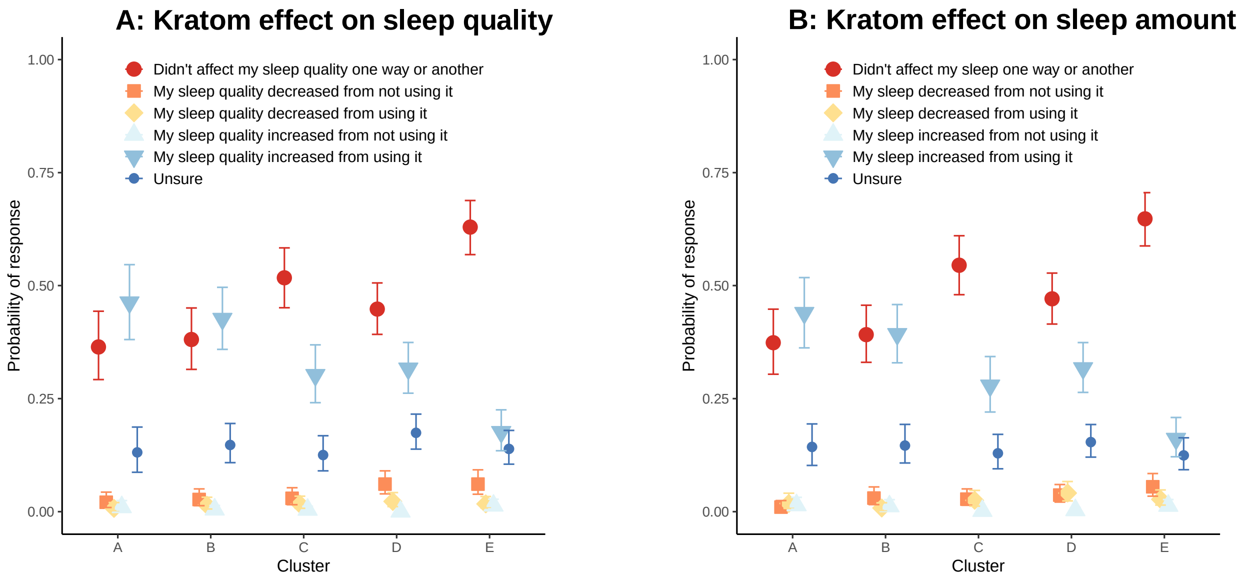

eFigure 9 . Assessments of how kratom taken yesterday affected (A) the quality and (B) the amount of sleep. Data are from beginning-of-day reports. These effects could be from the effects of kratom or from the effects of not using it. Other details of figure are as described for Fig. 1.

© 2024 Smith KE et al. *JAMA Network Open.*

eFigure 10. Conceptualizations of Kratom

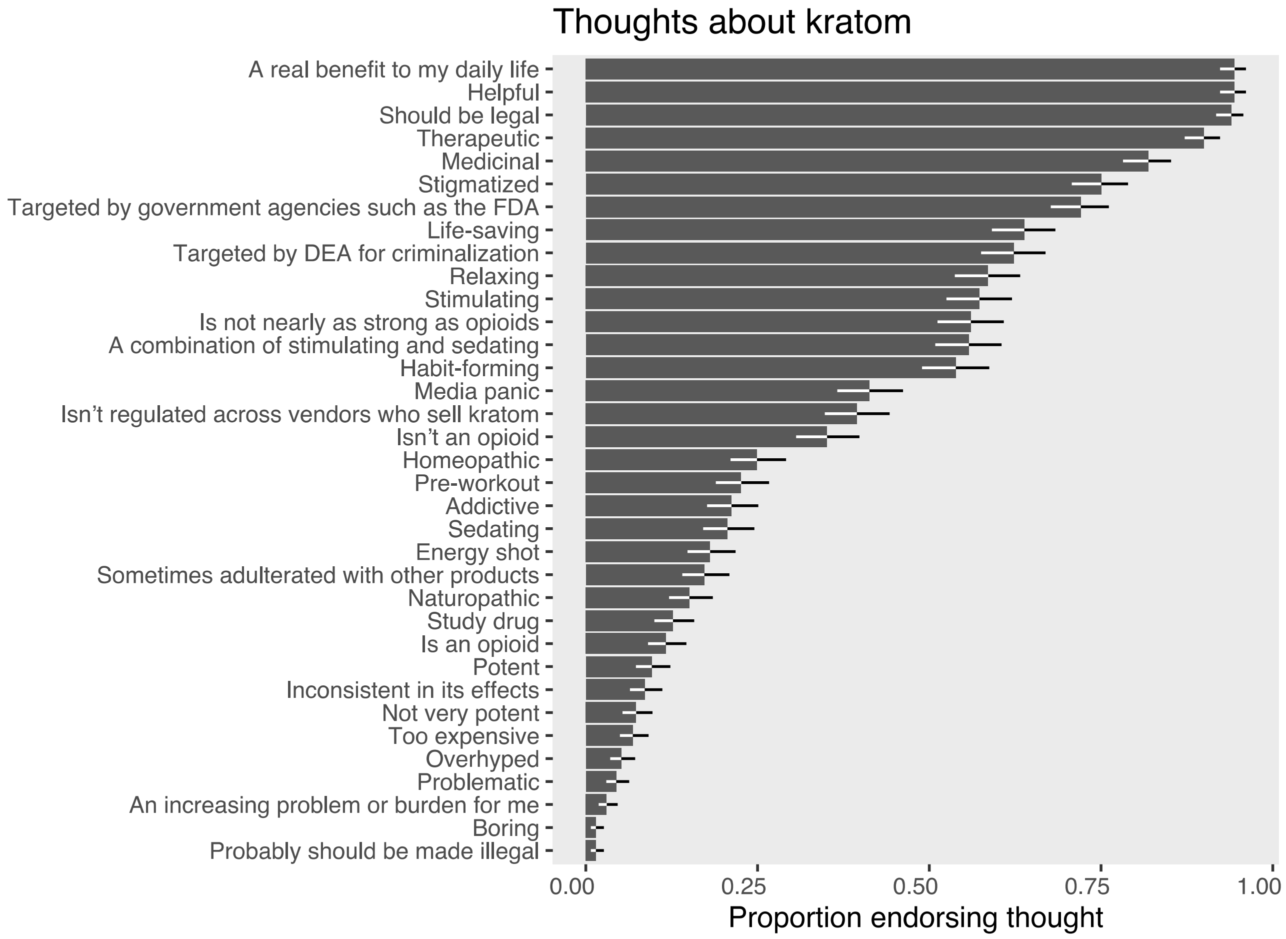

eFigure 10. Responses to the item, "How do you think of kratom? The list below has terms people could use to describe kratom or many other substances. Please select the terms that you would use to describe or conceptualize kratom. These can reflect both positive and negative aspects of kratom. There is no correct answer. Select all that apply." Mean proportions with 90% credible intervals.

© 2024 Smith KE et al. *JAMA Network Open.*
