# Supplementary material for: Ecological Momentary Assessment of Self-Reported Kratom Use, Effects, and Motivations Among US Adults
Source: JAMA Netw Open. 2024 Jan 26;7(1):e2353401. doi: 10.1001/jamanetworkopen.2023.53401 (PMC10818224; doi:10.1001/jamanetworkopen.2023.53401)
Supplement: Supplement 2. — Data Sharing Statement [file jamanetwopen-e2353401-s002.pdf]

Data Sharing Statement

Smith. Ecological Momentary Assessment of Self-Reported Kratom Use, Effects, and Motivations Among US Adults. *JAMA Netw Open*. Published January 26, 2024. doi:10.1001/jamanetworkopen.2023.53401

Data
Data available: No

Additional Information
Explanation for why data not available: As data were collected by NIDA IRP, a data sharing agreement will be needed. Requests for data collection instruments, datasets, and codebooks may be made to Drs. Kirsten Smith and David Epstein and will be reviewed on an individual basis.
